# Stereoselective Synthesis of Alkenyl Fluorides and Alkynes by Defluoro Coupling of Trifluoromethyl Arenes

**DOI:** 10.1002/anie.202515710

**Published:** 2025-10-20

**Authors:** José L. Rosario‐Collazo, Courtney Westlund, Hawa Keita, Simon J. Meek

**Affiliations:** ^1^ Department of Chemistry University of North Carolina at Chapel Hill Chapel Hill North Carolina 27599‐3290 USA

**Keywords:** Boron, C–F Activation, Defluorofunctionalization, Fluorine, Olefination

## Abstract

A practical method for the stereoselective synthesis of tri‐ and tetra‐substituted 1,2‐fluoro‐borylalkenes and alkynes by defluoro / C–C coupling of aryl trifluoromethyl groups is disclosed. Transformations convert simple abundant CF_3_–arenes and multifunctional organodiboron reagents in the presence of a Lewis base activator into coupled products in up to 94% yield and >98:2 Z/E selectivity. Synthetic utility is highlighted by several product transformations that access an array of diverse scaffolds.

## Graphical abstract

A practical, stereoselective method for synthesizing tri‐ and tetra‐substituted 1,2‐fluoro‐borylalkenes and alkynes via defluorinative C–C coupling of CF_3_–arenes with organodiboron reagents is reported. Enabled by a Lewis base activator, products are formed in up to 94% yield and >98:2 Z/E selectivity. The utility of the method is showcased through several product transformations.

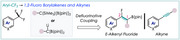

Stereodefined fluoroalkenes represent a valuable class of fluorinated compounds that have emerged as important structural motifs in organic synthesis, medicinal chemistry, and materials science.^[^ ^1^, ^2^, ^3^, ^4^, ^5^, ^6^, ^7^, ^8^, ^9^, ^10^, ^11^, ^12^ ^]^ Their distinct chemical and physical properties can enhance metabolic stability, modulate electronic characteristics, and influence molecular conformation.^[^ ^13^, ^14^, ^15^, ^16^, ^17^, ^18^, ^19^, ^20^, ^21^ ^]^ In particular, their resistance to enzymatic degradation coupled with their ability to serve as bioisosteres for amides and peptide bonds has garnered significance in the discovery of pharmaceutical compounds (Scheme 1a).^[^ ^22^, ^23^, ^24^, ^25^, ^26^ ^]^ Accordingly, several approaches for the stereoselective preparation of fluoroalkenes have been achieved.^[^ ^27^, ^28^, ^29^, ^30^, ^31^, ^32^, ^33^, ^34^, ^35^, ^36^, ^37^, ^38^ ^]^ In this regard, stereodefined fluoro‐borylalkenes have emerged as a valuable class of synthetic compounds that provide a versatile platform for the preparation of myriad stereodefined fluoroalkenes.^[^ ^39^ ^]^ Advances in synthetic approaches to these dual‐functional alkenes include transition‐metal‐catalyzed borylation,^[^ ^40^, ^41^, ^42^, ^43^, ^44^, ^45^, ^46^, ^47^, ^48^, ^49^ ^]^ regioselective hydroboration,^[^ ^50^ ^]^ and boron‐Wittig olefination (Scheme 1b).^[^ ^51^ ^]^ Despite these pioneering studies, all methods reported to‐date focus on 1,1‐fluoro‐borylalkenes, whereas methods to prepare the corresponding 1,2‐fluoro‐boryl isomers remain scarce.^[^ ^52^ ^]^ Due to the ubiquity of trifluoromethyl groups in bioactive compounds, the direct transformation of trifluoromethyl groups has become an attractive strategy for preparing fluorine‐containing molecules.^[^ ^53^ ^]^ Despite this potential, the defluorinative olefination of trifluoromethyl groups to prepare stereochemically defined fluoroalkenes or 1,2‐fluoro‐borylalkenes remains unexplored, though such a transformation would dramatically improve synthetic access to fluorinated molecular frameworks.

**Scheme 1:**
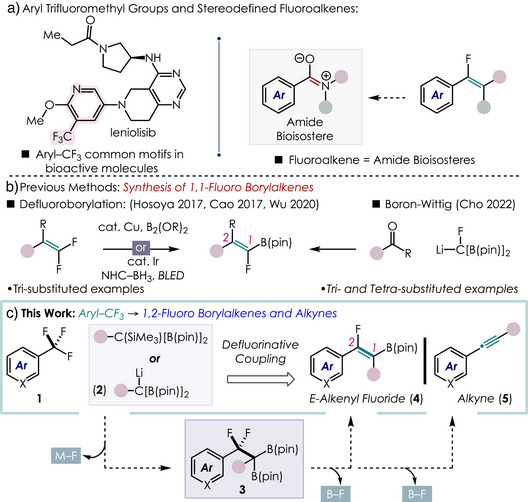
Synthesis of Fluoro Borylalkenes.

Advancing our studies into developing the unique transition metal‐free reactivity of organodiboron reagents for strategic transformations of value to organic synthesis,^[^ ^54^, ^55^, ^56^ ^]^ we hypothesized whether 1,2‐fluoro borylalkenes could be prepared by reaction of boron‐stabilized carbanions with aryl trifluoromethyl groups driven by B–F bond formation.^[^ ^51^, ^57^, ^58^, ^59^, ^60^, ^61^, ^62^, ^63^, ^64^, ^65^, ^66^, ^67^, ^68^, ^69^, ^70^, ^71^, ^72^, ^73^, ^74^, ^75^, ^76^, ^77^, ^78^, ^79^, ^80^, ^81^, ^82^, ^83^, ^84^ ^]^ Our strategy for such transition metal‐free aryl–CF_3_ coupling is outlined in Scheme 1c. Carbanion initiated reaction of a diboron‐stabilized nucleophile **2** with a CF_3_–substituted arene by SET reduction results in M–F loss and concurrent C–C bond formation.^[^ ^85^, ^86^, ^87^ ^]^ The resulting vicinal gem‐difluoro/gem‐diboryl intermediate **3** is primed to undergo facile substrate‐controlled B–F elimination to generate fluoro‐borylalkene **4**. Additionally, we considered whether this mechanistic pathway could be extended to produce alkynes (e.g., **5**) through a second B–F elimination, thereby achieving a complete defluorinative C–C coupling. This approach is supported by several anion initiated CF_3_‐arene couplings^[^ ^85^, ^86^, ^87^ ^]^ and the reactivity of boron‐stabilized carbanions.^[^ ^88^ ^]^ Herein, we present the first practical and effective method for the stereoselective synthesis of tri‐ and tetrasubstituted Z‐1,2‐fluoroalkenyl boronates by defluorinative olefination of trifluoromethyl arenes and gem‐diborylalkanes. Reactions proceed under mild conditions, delivering products in good yields with up to >98:2 Z/E selectivity. Furthermore, by modifying the Lewis base activation protocol, complete defluorinative coupling can be achieved to generate terminal and internal alkynes.

Toward the realization of this goal, we began by investigating the coupling of 2‐methoxy‐pyridine **6** with diboryl methane reagents (Table 1). Treatment of **6** with lithium carbanion **7a** (1.5 equiv.) in DME at 80 °C resulted in formation of **8** in up to 25% NMR yield (entry 1). We hypothesized that the low reactivity of **7a** might be due to coordinating nature of lithium versus larger, more dissociating ions such as K^+^, Cs^+^, or NR_4_^+^.^[^ ^89^ ^]^ Unfortunately, accessing these species proved challenging to achieve by deprotonation. Nonetheless, as an alternative approach we surmised that SiMe_3_–substituted reagent **7b** could be employed as a carbanion precursor by treatment with an appropriate fluoride source, which would promote loss of Me_3_Si–F and formation of the desired carbanion variants of **7a**. It was found that the coupling reaction between **6** and **7b** in the presence of CsF (2.0 equiv.) in DMF at 22 °C resulted in **8** in 19% NMR yield (entry 2). Application of other fluoride sources proved ineffectual (entries 3–5). However, conducting the reaction at 0.4 M and the addition of 18‐crown‐6 to increase the solubility of CsF generated desired Z‐fluoro‐boronate **8** in 52% NMR yield (entry 7). Combining this result with 3.0 equiv. of **7b** led to 79% **8** (98:2 *Z/E*) and optimal reaction conditions (entry 8).

**Table 1:** Reaction optimization^a)^, ^b)^

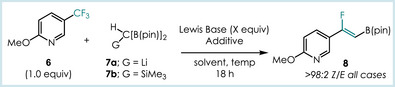						
Entry	**7a**–**b** (equiv.)	Lewis base (equiv.)	Additive (mol %)	Solvent (conc. M)	Temp (°C)	NMR Yield (%)^b)^
1	**7a**; (1.5)	–	–	DME (0.1 M)	80	25
2	**7b**; (2.0)	CsF (2.0)	–	DMF (0.1 M)	22	19
3	**7b**; (2.0)	KF (2.0)	–	DMF (0.1 M)	22	<2
4	**7b**; (2.0)	Me4NF (2.0)	–	DMF (0.1 M)	22	40
5	**7b**; (2.0)	TBAT (2.0)	–	DMF (0.1 M)	22	6
6	**7b**; (2.0)	CsF (2.0)	–	DMF (0.4 M)	22	33
7	**7b**; (2.0)	CsF (2.0)	18‐crown‐6 (40)	DMF (0.4 M)	22	52
8	**7b**; (3.0)	CsF (3.0)	18‐crown‐6 (40)	DMF (0.4 M)	22	79

^a)^ Reactions performed under N_2_ atmosphere.

^b)^ NMR yield, and Z/E ratios determined by analysis of crude reactions with mesitylene as internal standard. Reported yields are an average of at least two runs. See the Supporting Information for details.

We next examined the scope of the Z‐selective fluoroalkenyl boronate synthesis (Scheme 2). The transformation exhibits broad generality for a wide range of trifluoromethyl pyridines, accommodating diverse substituents at both the 2‐ and 3‐position. For example, bench‐stable Z‐fluoroalkenyl boronate products containing halogens (**8b**–**d**), amines (**8e** and **8j**), aryl and heteroaryl groups (**8f**, **8i**, and **8l**–**m**), alkynes (**8n**–**o**), and aryl ethers (**8g**–**h**) are obtained in high yields and with excellent Z‐selectivity (>98:2 *Z:E*). Notably, *N*‐allyl and *N*‐benzyl pyridones are tolerated, and undergo efficient coupling to deliver **8p** and **8q** in 57% and 50% yield, respectively. Trifluoromethyl arenes also participate in efficient, Z‐selective coupling reactions to afford *Z*‐fluoroalkenyl boronates. For instance, CsF‐mediated alkene formation from 3‐CF_3_‐ and 3‐CN‐substituted trifluoromethylbenzenes proceeds smoothly, furnishing compounds **8r** and **8s** in 77% and 96% yield, respectively, with >98:2 *Z/E*. Similar results are observed in the synthesis of substituted benzenes **8v–w**, which were isolated in moderate to good yields exclusively as the Z geometric isomers. Notably, 3,5‐bis(trifluoromethyl)anisole exclusively afforded double coupling product **8x**, and mono‐addition could not be achieved despite varying reaction conditions. The practicality of the method is showcased by the gram scale formation of **8k** (79% yield).

**Scheme 2:**
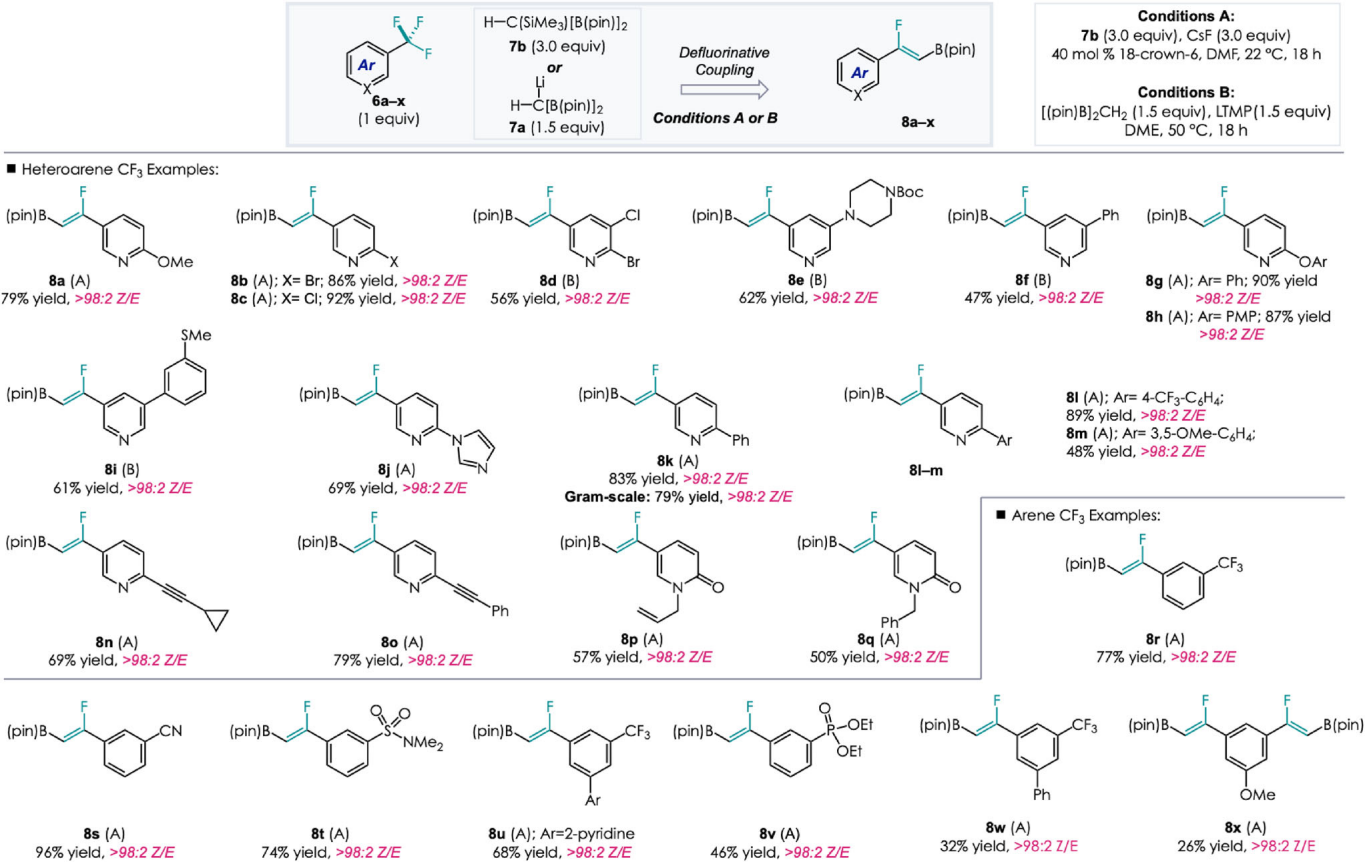
Stereoselective synthesis of *Z*‐Fluoroalkenyl Boronates. Reactions performed under N_2_ atmosphere. Z/E ratios determined by analysis of ^1^H NMR spectra of crude reactions with either mesitylene or DMF as internal standard. Reported yields are an average of at least two runs. See the Supporting Information for details.

To evaluate the influence of an additional carbon substituent on coupling efficiency, we set out to assess the protocol for the stereoselective transformation of aryl‐CF_3_ moieties into tetrasubstituted fluoroalkenes (Scheme 3). Treatment of 1,1,1‐ silyldiborylmethane with CsF in DMF at 22 °C results in effective formation of tetra‐substituted fluoroalkenes **9a**–**f** in good yields and up to 98:2 *E*/*Z* selectivity. In some cases, purification was complicated by co‐elution of starting material with the alkene E/Z isomers, yielding isomerically enriched products. As a result, crude NMR yields and E/Z ratios are also reported. Notably, the major E alkene stereoisomer arises from a stereoselective B–F elimination process that places the aryl and methyl groups in a cis configuration.^[^ ^90^ ^]^ The major *E* alkene isomer was assigned by nOe as well as confirmed through proto‐deboration and *J^2^*_F–H_ coupling (see Supporting Information for details). The observed *E*‐selectivity in the formation of the tetrasubstituted alkenes likely arises from a sterically controlled elimination pathway, governed by the size of B(pin).^[^ ^91^ ^]^ Extension to nucleophiles that incorporate a range of higher order carbon substituents also afforded the corresponding tetrasubstituted fluoroalkenes **9g**–**l** in synthetically useful yields, albeit with diminished E/Z ratios.

**Scheme 3:**
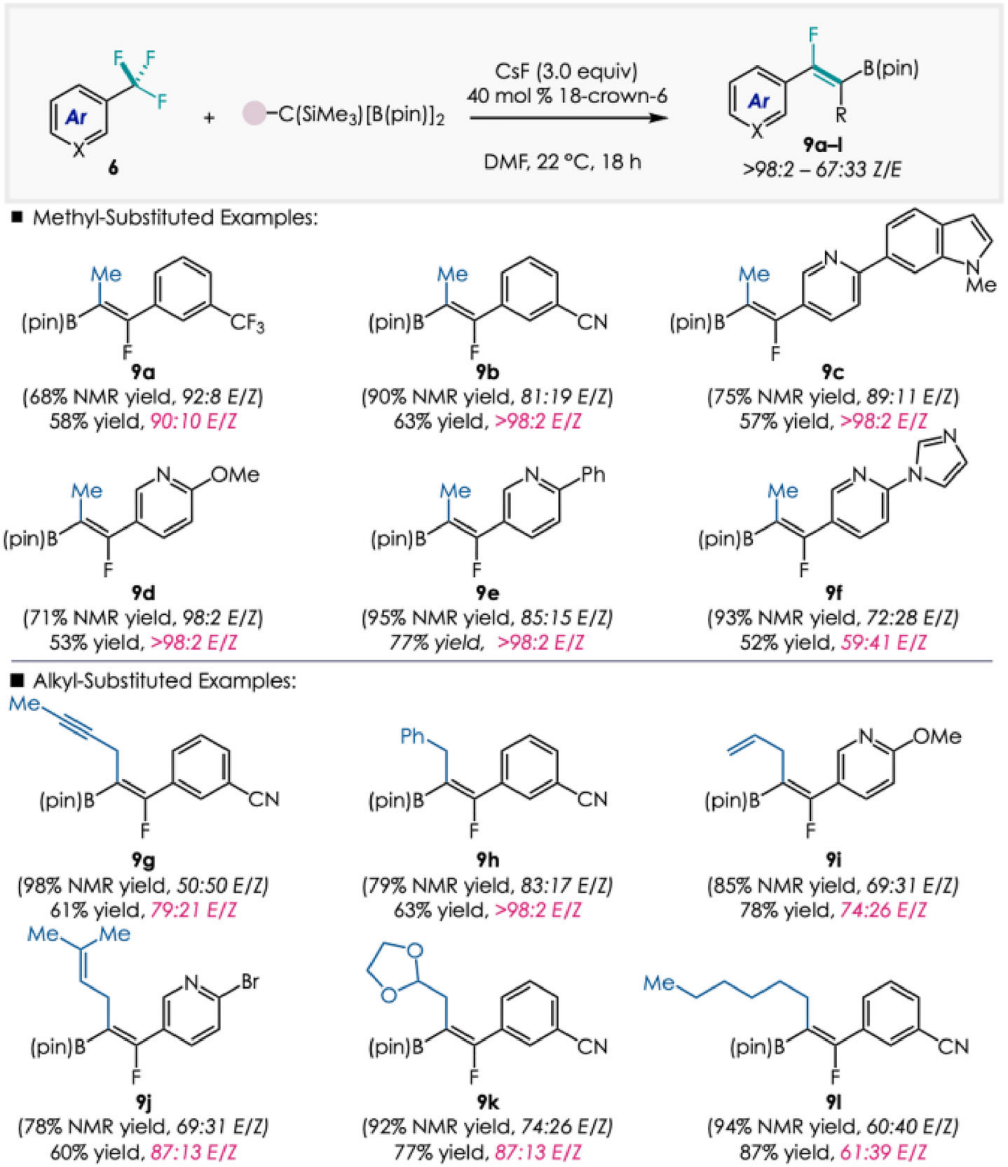
Stereoselective synthesis of Tetrasubstituted Fluoroalkenyl Boronates. Reactions performed under N_2_ atmosphere. NMR yield, and Z/E ratios determined by analysis of ^1^H NMR spectra of crude reactions with mesitylene as internal standard. Reported yields are an average of at least two runs. See the Supporting Information for details.

Although this strategy effectively yields tetrasubstituted fluoro‐borylalkenes with alkyl substituents, it fails to produce the corresponding 1,2‐diaryl variants owing to rapid B(pin) cleavage during both the reaction and subsequent purification. Unable to suppress this carbon–boron bond cleavage, we shifted our focus to optimizing the isolation of trisubstituted fluoroalkenes (Scheme 4). For instance, it was found reaction of [(pin)B]_2_HC–Ar with CsF in DME at 80 °C cleanly affords *E*‐trisubstituted fluoroalkenes in good yields and as single isomers (>98:2). The mild conditions prove similarly effective for arene variations in both the CF_3_ fragment as well as the diboron coupling partner (e.g., **11a**–**f**). In addition, the method is also amenable to derivatization of trifluoromethyl‐containing bioactive molecules; for example, fluoxetine‐derived **11g** is fashioned in 22% yield and >98:2 E/Z.

**Scheme 4:**
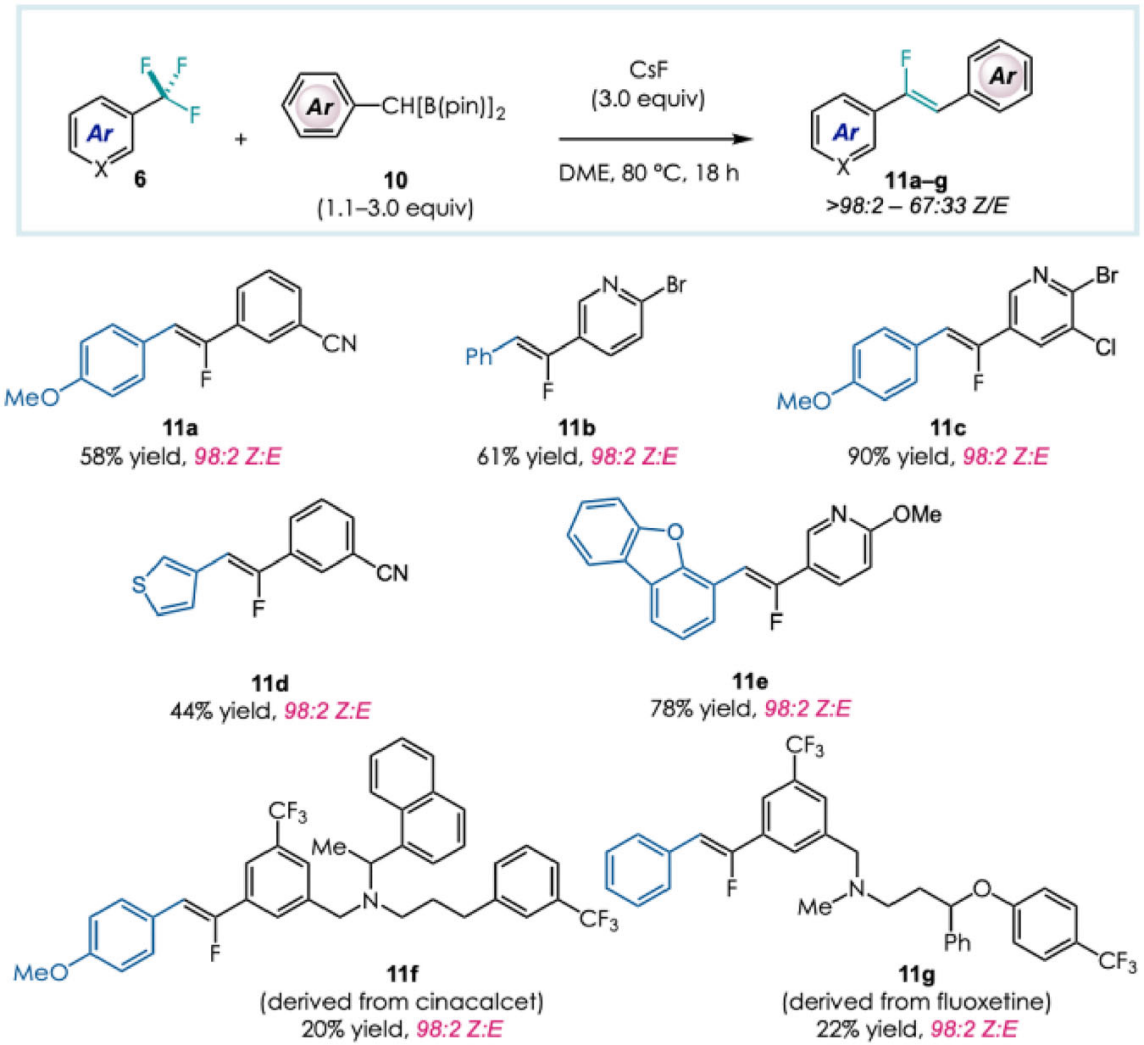
Stereoselective synthesis Z‐Fluoroalkenes. Reactions performed under N_2_ atmosphere. Z/E ratios determined by analysis of ^1^H NMR spectra of crude reactions with mesitylene as internal standard. Reported yields are an average of at least two runs. See the Supporting Information for details.

Based on the vicinal arrangement of the fluorine and B(pin) functionality in the alkenyl products, we postulated if the coupling approach could be expanded to enable the direct conversion of aryl–CF_3_ groups into alkynes. To achieve an exhaustive defluorinative transformation, conditions that facilitate concomitant elimination of the residual C(sp^2^)–B and C(sp^2^)–F groups is required. Accordingly, it was found that simply conducting the coupling processes with lithiated diborylalkane in the presence of lithium *tert*‐butoxide (1.5 equiv) in DME at 80 °C for 18 h results in an effective single‐step protocol for the direct synthesis of terminal and internal alkynes (Scheme 5). A range of trifluoromethyl‐substituted pyridines can be directly converted into terminal alkynes in good yields; for example, compounds **12a–g** are obtained in 50%–84% yield. Similarly, a variety of substituted diborylalkanes undergo C–C coupling followed by exhaustive defluorination with trifluoromethyl arenes to deliver internal alkynes (e.g., **12j–l**) in excellent yields. Conversion of CF_3_‐arenes is also achievable; however, due to the incompatibility between CsF and LiOtBu, a telescoped rather than one‐pot procedure is required. For example, CF_3_ coupling with **7b** and CsF followed by elimination with LiOtBu furnishes alkynes **12h**–**i** in good yields over the two‐step sequence. To determine whether terminal alkyne formation proceeds through a lithium acetylide intermediate, electrophile quenching experiments were performed. The complete absence of internal alkyne products supports a deborylative–fluorine elimination pathway.

**Scheme 5:**
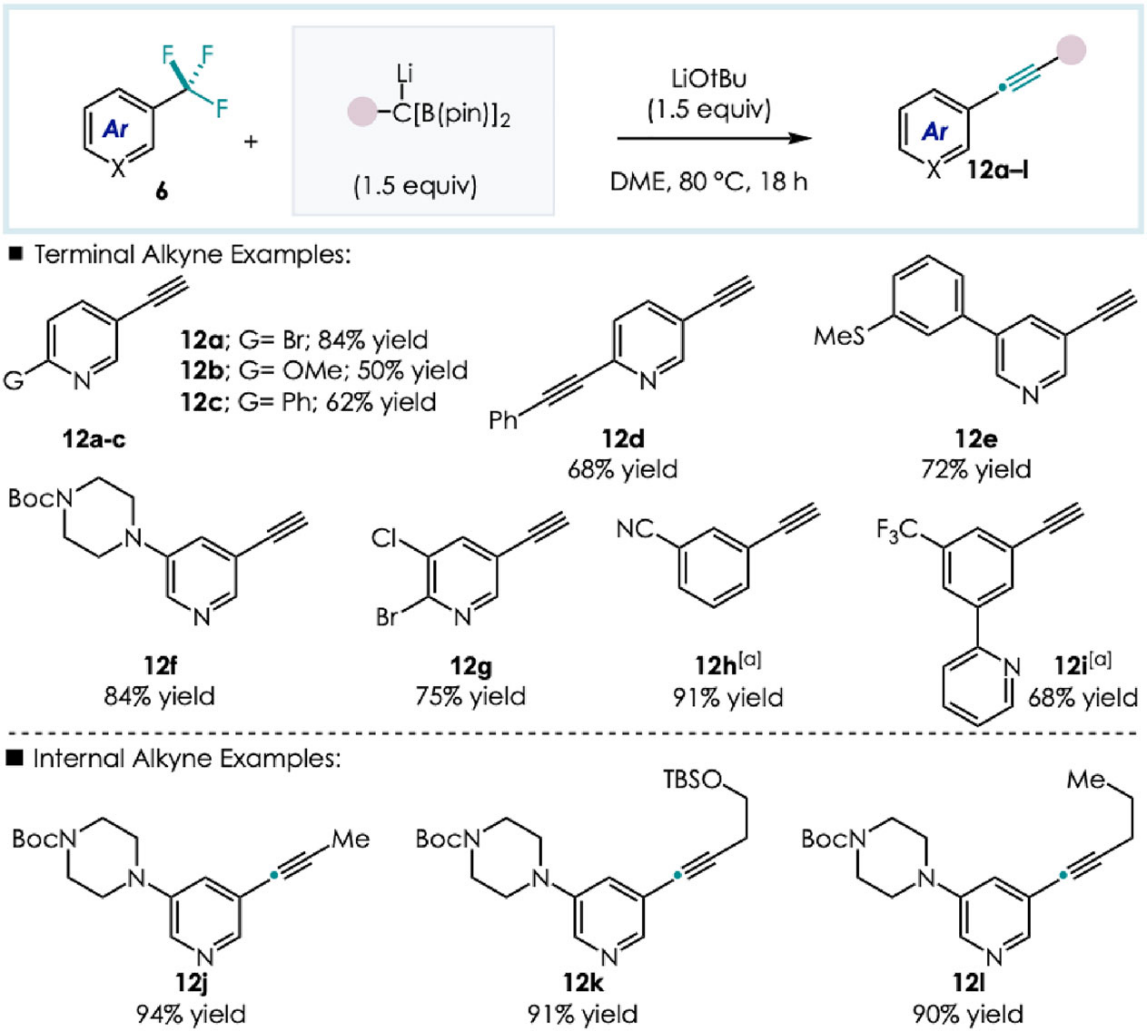
Synthesis of alkynes by tris‐defluorinative carbon–carbon bond coupling. Reactions performed under N_2_ atmosphere. Reported yields are an average of at least two runs. ^a)^ Two‐step process with **7b**, see the Supporting Information for details.

The alkenyl fluoride and alkyne products obtained from these coupling protocols enable diverse downstream transformations, as shown in Scheme 6. For example, the resulting fluoroalkenyl boronates can be oxidized directly to generate α‐fluoroketones, as demonstrated by compounds **13** and **14**, which were formed in 82% and 64% yield, respectively. The stereo‐defined *Z*‐ and *E*‐ fluoroalkenyl boronates also serve as valuable intermediates for the synthesis of tri‐ and tetrasubstituted fluoroalkenes through Pd‐catalyzed cross‐coupling reactions, as evidenced by the preparation of compounds **15–17**. Similarly, the terminal and internal alkyne products represent adaptable scaffolds that can be transformed in a variety of ways, including by the formation of triazole **18** via copper‐catalyzed azide‐alkyne cycloaddition, and the Pd‐catalyzed synthesis of indole **19** and benzofuran **20**. Collectively, these transformations provide valuable synthetic avenues for the late‐stage modification of aryl–trifluoromethyl groups in complex molecule synthesis.

**Scheme 6:**
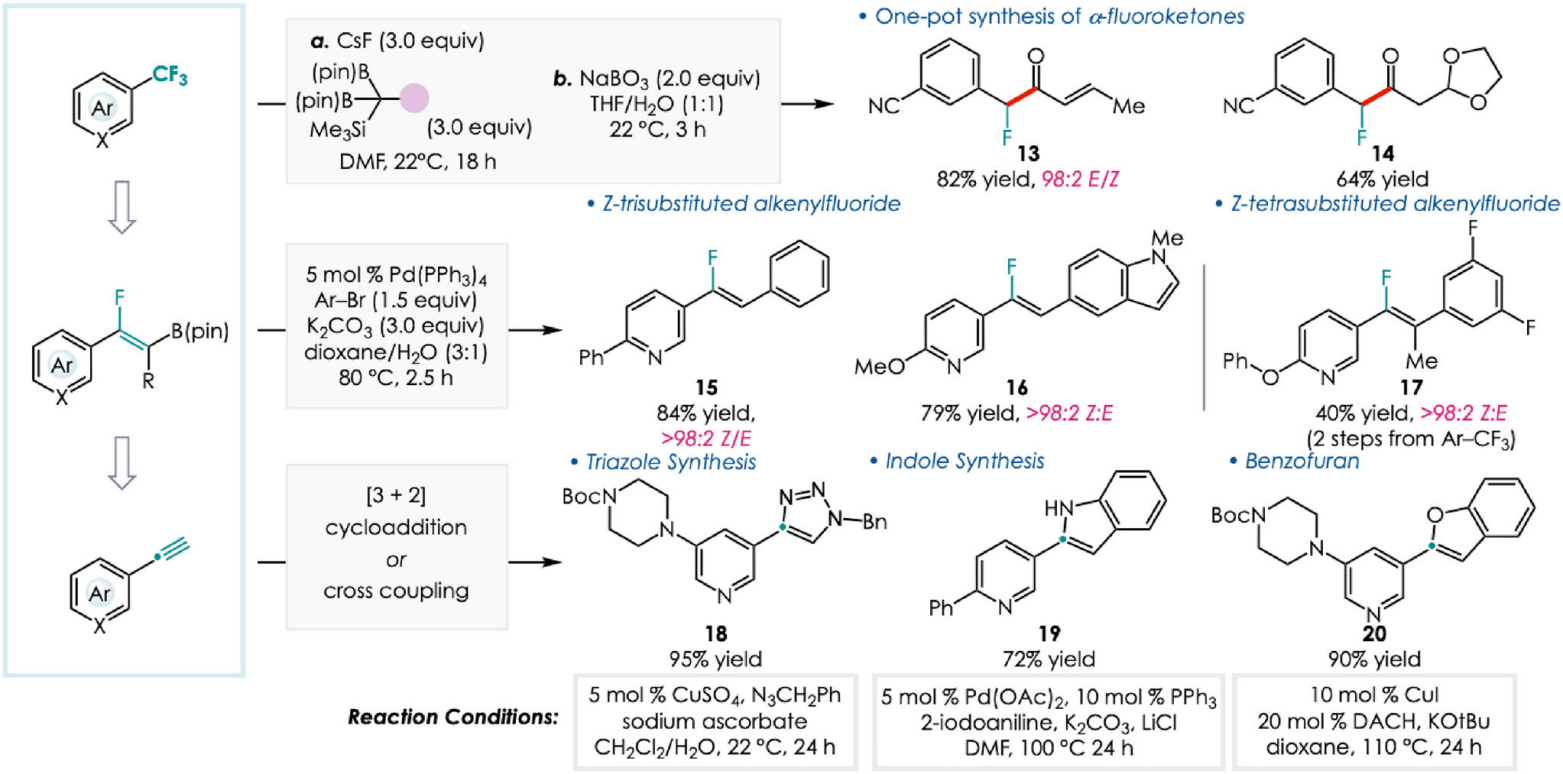
Synthetic utility of Ddeflurorinative couplings. Reactions performed under N_2_ atmosphere. See the Supporting Information for details.

Next, we set out to address whether activation of SiMe_3_–substituted reagent **7b** with CsF results in activation of B(pin) or SiMe_3_ and formation of a B(pin)/B(pin) or a SiMe_3_/B(pin) stabilized carbanion. Notably, reaction through a SiMe_3_/B(pin) stabilized carbanion could result in Si–F elimination to also afford the observed alkenylboronate products. To probe the nature of the reactive species generated in reactions with **7b**, we performed an electrophile trapping experiment. Subjecting **7b** and benzyl bromide to standard conditions (Scheme 2) results in exclusive formation of diboron product **22** (66% yield). To probe the mechanism by which fluorine interacts with **7b**, we treated **7b** (1.0 equiv.) with CsF (1.0 equiv.) and 10 mol % 18‐cr‐6 in CD_3_CN at 22 °C.^[^ ^92^ ^]^ Analysis of the mixture by ^19^F NMR at 30 min shows three distinct signals with the following relative abundance: Me_3_SiF (**28**, 58%),^[^ ^93^ ^]^ a fluoro‐boronate species (**27**, 6%), and an unknown intermediate tentatively assigned as a siliconate **26** (36%). After 4 h both **26** and **27** disappear and only Me_3_SiF is observed.^[^ ^94^ ^]^ Lastly, the addition of TEMPO (a radical scavenger) to the coupling reactions results in near‐complete suppression of product formation, confirming the involvement of radical intermediates; for example, the formation of **8a** is reduced from 80% to only 10% NMR yield. Together, these data indicate that 1) the defluorinative olefination/alkynylation proceeds by radical intermediates, 2) Si–C bonds are preferentially activated over B–C bonds, resulting in formation of a more stable diboron‐stabilized carbanion;^[^ ^95^ ^]^ and 3) fluoro‐boronate species exist in equilibrium with the Si–C bond activation pathway. Lastly, a proposed stereochemical rationale is depicted in Scheme 7d to explain the alkenyl fluoride selectivity observed in the B–F elimination step. For H and Me substituted nucleophiles, the larger B(pin) group occupies the position anti to Ar (e.g., **A**→**B**), whereas for aryl‐substituted nucleophiles the smaller B(pin) resides syn to Ar (e.g., **B**→**D**).^[^ ^91^ ^]^

**Scheme 7:**
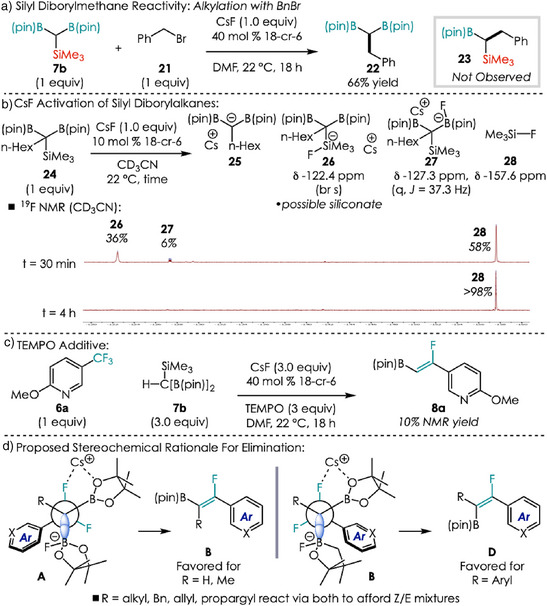
Mechanism experiments. See the Supporting Information for details.

In conclusion, an effective coupling method for the stereoselective synthesis of stereo‐defined fluoroalkenyl boronates and alkynes from trifluoromethyl arenes has been described. This method utilizes the reactivity of boron‐stabilized carbanions and is effective for a range of arene and heteroarene substrates delivering bench‐stable fluoroalkenes in up to >98:2 selectivity. Fluoride activation of *gem*‐diboryl‐silyl groups is employed as an efficient strategy for carbanion generation, which proceeds by selective C–Si bond activation versus C–B cleavage. When coupled with a lithium alkoxide, protocols for the direct conversion of trifluoromethyl groups to alkynes is realized through consecutive fluoride eliminations. The approach provides valuable avenues for derivatizing CF_3_ groups, functionality abundant in bioactive compounds. Ongoing efforts aim to expand the scope of boron‐mediated CF_3_ functionalizations.

## Conflict of Interests

The authors declare no conflict of interest.

## Supporting information



Supporting Information

## Data Availability

The data that support the findings of this study are available in the supplementary material of this article.
