# Supplementary material for: Stereoselective Synthesis of Alkenyl Fluorides and Alkynes by Defluoro Coupling of Trifluoromethyl Arenes
Source: Angew Chem Int Ed Engl. 2025 Oct 20;64(48):e202515710. doi: 10.1002/anie.202515710 (PMC12636270; doi:10.1002/anie.202515710)
Supplement: Supplementary file 1 — Supporting Information [file ANIE-64-e202515710-s001.docx]

**Stereoselective Synthesis of Alkenyl Fluorides and Alkynes By Defluoro Coupling of Trifluoromethyl Arenes**

José L. Rosario-Collazo, Courtney Westlund, Hawa Keita, Simon J. Meek*

*Department of Chemistry, University of North Carolina at Chapel Hill, North Carolina 27599-3290, United States*

**Supporting Information**

**Table of Contents**

**General**…………………………………………………………………………………………………..…………**S3**

**Solvents**…………………………………………………………………………………………...………...………**S3**

**Reagents**……………………………………………………………………………………………………………**S3**

**Synthesis of Alkyl Substituted 1,1-diborons** ……………………………….…………………………………**S4**

**Synthesis of Alkyl Substituted TMS 1,1-diborons** ……………………………….……………………………**S5**

**Synthesis of Radical Trap…………**……………………………….……………………………………..………**S7**

**Synthesis of Trifluoromethyl Arenes**…………………………….………………………………………………**S8**

**Synthesis of Aryl Substituted TMS 1,1-diborons**..………….…………………….…………………………..**S15**

**General procedure for Z-Fluoroalkenyl Boronates**……………………………………………..…………**S16**

**General Procedure for Synthesis of Z- and E-Tetrasubstituted Fluoroalkenyl Boronates**…….…….**S23**

**Identification of E/Z selectivity procedure**…………………………………………………………….……**S28**

**General procedure for Synthesis of Z-Fluoroalkenes**……………………………………………………..**S31**

**Synthesis of Alkyne by tris-defluorinative C-C bond coupling**……………………………………….…**S33**

**Synthesis of internal alkynes**…………………………………………………………………………………...**S36**

**Synthetic utility of alkenyl fluoride compounds**……………………………………………………………**S37**

**Mechanistic Investigation**………………………………………………………………………………...……**S42**

**Gram Scale Reaction…………………………………………………………………………………………...S44**

**References**…………………………..……………………………………………………………………………**S45**

**^1^H, ^13^C, ^19^F, and ^11^B NMR Spectra**………………………………………………………………………..……**S48**

**■** **General Analysis**: All compounds were characterized by ^1^H, ^13^C and ^19^F NMR spectroscopy, and mass spectroscopy using high-resolution mass spectrometry (Q Exactive HF-X, ThermoFisher, Bremen, Germany). NMR spectra were recorded on a Bruker AVANCE NEO600, NEO400 and B600 equipped with a CryoQNP probe. NMR data are reported as follows: chemical shift, integration, multiplicity (s = singlet, d = doublet, t = triplet, dd = doublet of doublets, td = triplet of doublets, dt = doublet of triplets, ddd = doublet of doublets of doublets, m = multiplet, bs = broad singlet), and coupling constants (Hz).

**■ General Reagent Information**: The diboron compounds were synthesized following the corresponding literature procedures. Similarly, the trifluoromethyl arenes were prepared using established literature methods. Anhydrous 1,2-dimethoxyethane was dried and distilled using sodium and benzophenone. DMF was dried and distilled using molecular sieves. THF and MeCN were used from a solvent system equipped with molecular sieves. Column chromatography was performed using silica gel from Sigma Aldrich.

**■ Reagents:**

**2-bromo-5-trifluoromethyl pyridine:** was purchased from Matrix Scientific, dried via azeotropic distillation, and stored in a nitrogen filled glovebox.

**2-chloro-5-trifluoromethyl pyridine:** was purchased from Sigma Aldrich, dried via azeotropic distillation, and stored in a nitrogen-filled glovebox.

**2-methoxy-5-trifluoromethyl pyridine:** was purchased from Sigma Aldrich, dried via azeotropic distillation, and stored in a nitrogen-filled glovebox.

**2-bromo-3-chloro-5-(trifluoromethyl)pyridine:** was purchased from Sigma Aldrich, dried via azeotropic distillation, and stored in a nitrogen-filled glovebox.

**1,3-Bis(trifluoromethyl)benzene:** was purchased from Matrix Scientific, dried via azeotropic distillation, and stored in a nitrogen-filled glovebox.

**3-(Trifluoromethyl)benzonitrile:** was purchased from Oakwood, dried via azeotropic distillation, and stored in a nitrogen-filled glovebox.

**18-crown-6:** was purchased from Strem, used as is, and stored in a nitrogen-filled glovebox.

**Chlorotrimethylsilane:** was purchased from Oakwood and distilled with quinoline.

**Cesium fluoride (extra pure 99.99%)**: was purchased from Across Organic, dried using a drying pistol, and stored in a nitrogen-filled glovebox.

**Tetrakis(triphenylphosphine)palladium(0):** was purchased from Strem Chemicals and stored at -20 °C in a nitrogen-filled glovebox.

**LiOMe (99.99% purity)**: was purchased from Strem Chemicals and sealed under an inert atmosphere.

**KOtBu:** was purchased from Strem Chemicals and sealed under an inert atmosphere

**LiOtBu:** was purchased from Strem Chemicals and sealed under an inert atmosphere

**Grubbs (II) catalyst**: was purchased from Sigma Aldrich and used without any purification method.

**Potassium carbonate:** was purchased from VWR Life Science and used without additional purification.

**N,N-Dimethylformamide (anhydrous)**: was purchased from Sigma Aldrich, dried via distillation with molecular sieves, and stored in a nitrogen-filled glovebox.

**Copper (I) Iodide:** was purchased from Strem Chemicals, used as is, and stored in a nitrogen-filled glovebox.

**Triethylamine**: used from solvent system dried under molecular sieve.

**Benzyl azide**: was purchased from Alpha Aesar and used as is.

**Sodium Perborate tetrahydrate:** was purchased from Sigma Aldrich and used as is.

**2-iodoaniline:** was purchased from Sigma Aldrich and used without further purification.

**2-iodophenol:** was purchased from Sigma Aldrich and used without further purification.

**Bromobenzene:** was purchased from Sigma Aldrich and used without further purification.

**1-bromo-3,5-difluorobenzene:** was purchased from Matrix Scientific and used without further purification.

**5-bromo-1- methylindole:** was purchased from Sigma Aldrich and used without further purification.

**(1R,2R)-(−)-1,2-Diaminocyclohexane:** was purchased from Sigma Aldrich and used without further purification.

**Mesitylene:** was purchased from Sigma Aldrich and used without further purification.

**■Synthesis of 1,1 Diboron compounds:**

**2,2'-(butane-1,1-diyl)bis(4,4,5,5-tetramethyl-1,3,2-dioxaborolane) (S2) (Procedure a):** In a nitrogen-filled glovebox, a flame-dried flask equipped with a magnetic stir bar was charged with LiDBM (1.46 mmol) and anhydrous THF (0.15M). The reaction mixture was cooled to 0 °C using an ice bath; at this temperature, the LiDBM did not fully dissolve. 1-Iodopropane (2.5) was added dropwise via syringe, upon which the contents fully dissolved. The reaction mixture was allowed to warm to room temperature (22 °C) and stirred for 18 hours.

Afterward, the reaction was quenched with 50 mL of saturated aqueous NH₄Cl. The resulting biphasic mixture was extracted with diethyl ether. The combined organic layers were dried over MgSO₄, filtered, and concentrated under reduced pressure. The crude product was purified by silica gel column chromatography (hexanes/ethyl acetate, 20:1)to afford the desired diboryl product **(S2)**. All the spectras matched the literature data.^1^

**(3,3-bis(4,4,5,5-tetramethyl-1,3,2-dioxaborolan-2-yl)propoxy)(tert-butyl)dimethylsilane (S4):** 1,1-diboron was synthesized according to procedure a. All the spectra matched the literature data.^1^

**■Synthesis of TMS 1,1-Diboron Compound:**

To an oven-dried 50 mL round-bottom flask equipped with a magnetic stir bar was added diisopropylamine (41.0 mmol, 1.1 equiv) and THF (10 mL). The solution was cooled to –23 °C (9:1 ethylene glycol:ethanol and dry ice bath), and n-BuLi [2.5 M in hexanes] (42.9 mmol, 1.1 equiv) was added dropwise at –23 °C. The resulting mixture was allowed to stir for 30 minutes at –23 °C to form LDA. In a separate oven-dried 100 mL round-bottom flask, bis[(pinacolato)boryl]methane (37.3 mmol, 1.0 equiv) was dissolved in THF (46 mL). The preformed LDA solution was quickly transferred into the DBM solution by syringe, which was subsequently cooled back to –23 °C and allowed to react for an additional 30 minutes. A white precipitate formed upon mixing. The reaction mixture (LiDBM) was then warmed to 0 °C, and TMSCl (41.0 mmol, 1.1 equiv) was added. The reaction was allowed to stir at 22 °C for 2 hours. Upon completion, the mixture was quenched with water (50 mL) and extracted three times with ethyl acetate. The combined organic layers were dried with Na₂SO₄, filtered, and concentrated under reduced pressure. The crude product was purified by column chromatography (hexanes/ethyl acetate, 10:1) to afford the desired product **(7b)**. Spectroscopic data matched previously reported values.^2^

**^1^H NMR (600 MHz, CDCl_3_)** δ 1.21 (s, 12H), 1.19 (s, 12H), 0.27 (s, 1H), 0.08 (s, 9H).

**■General Procedure b for synthesis of Alkyl Substituted TMS 1,1-diborons**:

To an oven-dried round-bottom flask, TMS-DBM (1.0 equiv) was added and dissolved in THF. A In a separate flask, LTMP (1.1 equiv) was dissolved in THF (0.15 M) and transferred to the TMS-DBM solution at 0 °C. The reaction mixture was allowed to stir at 0 °C for 30 minutes. In some cases, a white precipitate (LiTMS-DBM) formed within a few minutes. An aliphatic halide (check specific substrate for equivalent) was then added at 0 °C, and the reaction was allowed to stir for 18 hours at 22 °C. The reaction mixture was quenched with water, extracted with ethyl acetate, and the organic layer was dried over Na₂SO₄. After filtration and concentration, the crude product was purified by column chromatography to afford the desired product.

**(1,1-bis(4,4,5,5-tetramethyl-1,3,2-dioxaborolan-2-yl)ethyl)trimethylsilane (S6):**

General procedure b was followed using iodomethane (2.5 eq). The product was purified by column chromatography (hexane; ethyl acetate 10:1) to give the desired product in 83% as a white solid.

**^1^H NMR (600 MHz, CDCl_3_)** δ 1.20 (s, 12H), 1.19 (s, 12H), 1.12 (s, 3H), 0.07 (s, 9H).

**^13^C NMR (151 MHz, CDCl_3_)** δ 82.47, 24.95, 24.53, 11.77, -1.61.

**HRMS (ESI+) [M+Na]+** calcd for C_17_H_36_B_2_NaO_4_Si^+^: 377.2461; found: 377.2461

**(1,1-bis(4,4,5,5-tetramethyl-1,3,2-dioxaborolan-2-yl)pent-3-yn-1-yl)trimethylsilane (S7):**

General procedure S2 was followed using 1-bromo-2-Butyne (2.5 eq). The product was purified by column chromatography (hexane/ethyl acetate 10:1) to give the desired product in 87% as a liquid.

**^1^H NMR (600 MHz, CDCl_3_)** δ 2.43 (d, *J* = 2.5 Hz, 2H), 1.70 (t, *J* = 2.4 Hz, 3H), 1.21 (s, 12H), 1.21 (s, 12H), 0.12 (s, 9H).

**^13^C NMR (151 MHz, CDCl_3_)** δ 82.65, 81.96, 74.28, 24.83, 24.61, 17.10, 3.51, -0.79.

**HRMS (ESI+) [M+Na]+** calcd for C_20_H_38_B_2_NaO_4_Si^+^: 415.2618; found: 415.2618

**Trimethyl(2-phenyl-1,1-bis(4,4,5,5-tetramethyl-1,3,2-dioxaborolan-2-yl)ethyl)silane(S8):**

General procedure S2 was followed using benzyl bromide (2.5 eq). The product was purified by column chromatography (hexane/ethyl acetate 10:1) to give the desired product in 85% as a white solid. The spectrum matches the literature. ^3^

**^1^H NMR (600 MHz, CDCl_3_)** δ 7.35 (d, *J* = 7.4 Hz, 2H), 7.16 (t, *J* = 7.6 Hz, 2H), 7.08 (t, *J* = 7.3 Hz, 1H), 3.02 (s, 2H), 1.16 (s, 12H), 1.14 (s, 12H), 0.06 (s, 9H).

**(1,1-bis(4,4,5,5-tetramethyl-1,3,2-dioxaborolan-2-yl)heptyl)trimethylsilane (S9)**

General procedure S2 was followed using 1-bromohexane (2.5 eq). The product was purified by column chromatography (hexane/ethyl acetate 10:1) to give the desired product in 81% as a liquid.

**^1^H NMR (600 MHz, CDCl_3_)** δ 1.57 – 1.50 (m, 2H), 1.45 – 1.34 (m, 2H), 1.31 – 1.23 (m, 7H), 1.20 (s, 12H), 1.19 (s, 12H), 0.88 (t, *J* = 7.0 Hz, 3H), 0.07 (s, 9H).

**^13^C NMR (151 MHz, CDCl_3_)** δ 82.21, 31.89, 31.20, 30.39, 24.95, 24.73, 22.68, 14.15, -0.74.

**HRMS (ESI+) [M+Na]+** calcd for C_22_H_46_B_2_NaO_4_Si^+^: 447.3244; found: 447.3248.

**Trimethyl(4-methyl-1,1-bis(4,4,5,5-tetramethyl-1,3,2-dioxaborolan-2-yl)pent-3-en-1-yl)silane (S10):**

General procedure S2 was followed using 1-bromo-3-methylbut-2-ene (2.5 eq). The product was purified by column chromatography (hexane/ethyl acetate 10:1) to give the desired product in 83% as a colorless oil.

**^1^H NMR (600 MHz, CDCl_3_)** δ 5.20 (tt, *J* = 7.0, 1.3 Hz, 1H), 2.30 (d, *J* = 7.0 Hz, 2H), 2.02 – 1.54 (m, 6H), 1.19 (s, 12H), 1.18 (s, 12H), 0.07 (s, 9H).

**^13^C NMR (151 MHz, CDCl_3_)** δ 129.33, 127.64, 82.45, 26.52, 26.15, 25.07, 24.80, 18.14, -0.55.

**HRMS (ESI+) [M+Na]+** calcd for C_19_H_38_B_2_NaO_4_Si^+^: 431.2931; found: 431.2933

**(1,1-bis(4,4,5,5-tetramethyl-1,3,2-dioxaborolan-2-yl)but-3-en-1-yl)trimethylsilane (S11):**

General procedure S2 was followed using allyl bromide (2.5 eq). The product was purified by column chromatography (hexane/ethyl acetate 10:1) to give the desired product in 89% as a colorless oil.

**^1^H NMR (600 MHz, CDCl_3_)** δ 5.96 (ddt, *J* = 17.0, 10.0, 6.9 Hz, 1H), 4.99 (dd, *J* = 17.1, 2.2 Hz, 1H), 4.91 – 4.66 (m, 1H), 2.38 (d, *J* = 6.9 Hz, 2H), 1.20 (s, 12H), 1.19 (s, 12H), 0.09 (s, 9H).

**^13^C NMR (151 MHz, CDCl_3_)** δ 141.08, 113.83, 82.42, 32.48, 25.01, 24.71, -0.67.

**HRMS (ESI+) [M+Na]+** calcd for C_19_H_38_B_2_NaO_4_Si^+^: 403.2618; found: 403.2621

**(2-(1,3-dioxolan-2-yl)-1,1-bis(4,4,5,5-tetramethyl-1,3,2-dioxaborolan-2-yl)ethyl)trimethylsilane (S12):**

General procedure S1 was followed using 2-(bromomethyl)-1,3-dioxolane (1.5 eq). The product was purified by column chromatography (hexane/ethyl acetate 10:1) to give the desired product in 27% as a white solid.

**^1^H NMR (600 MHz, CDCl_3_)** δ 5.33 (t, *J* = 5.6 Hz, 1H), 4.16 – 3.90 (m, 2H), 3.86 – 3.73 (m, 2H), 1.86 (d, *J* = 5.6 Hz, 2H), 1.21 (s, 12H), 1.19 (s, 12H), 0.09 (s, 9H).

**^13^C NMR (101 MHz, CDCl_3_)** δ 105.52, 82.37, 64.37, 32.55, 25.14, 24.71, -1.04.

**HRMS (ESI+) [M+Na]+** calcd for C_20_H_40_B_2_NaO_6_Si^+^: 449.2672; found: 449.2678

**■Synthesis of Radical Trap Diboron**

**N'-(cyclopropylmethylene)-4-methylbenzenesulfonohydrazide**: A round-bottom flask was charged with formylcyclopropane (10 mmol) and tosylhydrazine (10 mmol). Then methanol (5 mL) was added. The mixture was allowed to stir at room temperature. N-Tosylhydrazone precipitated after 1 h and the reaction was monitored by TLC analysis. Once the reaction had completed, the precipitate was collected, washed with ether (5 mL × 3), dried in vacuum, and used without further purification.

**2,2'-(cyclopropylmethylene)bis(4,4,5,5-tetramethyl-1,3,2-dioxaborolane)**: A round-bottom flask was charged with N-tosylhydrazone (1 mmol) and 60% NaH (1.2 mmol, 48 mg). After degassed and filled with N2, the tube was charged with toluene (8 mL). The mixture was allowed to stir at room temperature for 1 h. Then, a solution of B_2_pin_2_ (1.2 mmol, 305 mg) in toluene (2 mL) was added via syringe. Then the tube was sealed and heated at 110 °C for 12 h. After, the reaction was allowed to cool to room temperature and then diluted with 10 mL of Et_2_O and 10 mL of H_2_O. The mixture was stirred vigorously for 10 minutes. After separation of organic layer, the aqueous layer was extracted with Et_2_O (5 mL×2). The combined organic solution was washed with brine (10 mL) and dried over anhydrous Na_2_SO_4_. After the solvent was evaporated, the crude product was purified by silica gel chromatography. Spectroscopic data matched previously reported values.^33^

**(cyclopropylbis(4,4,5,5-tetramethyl-1,3,2-dioxaborolan-2-yl)methyl)trimethylsilane**: To an oven-dried 50 mL round-bottom flask equipped with a magnetic stir bar was added diisopropylamine (41.0 mmol, 1.1 equiv) and THF (10 mL). The solution was cooled to –23 °C (9:1 ethylene glycol:ethanol and dry ice bath), and n-BuLi [2.5 M in hexanes] (42.9 mmol, 1.1 equiv) was added dropwise at –23 °C. The resulting mixture was allowed to stir for 30 minutes at –23 °C to form LDA. In a separate oven-dried 100 mL round-bottom flask, the cyclopropyl-substituted diboron (37.3 mmol, 1.0 equiv) was dissolved in THF (46 mL). The preformed LDA solution was quickly transferred into the DBM solution by syringe, which was subsequently cooled back to –23 °C and allowed to react for an additional 30 minutes. A white precipitate formed upon mixing. The reaction mixture was then warmed to 0 °C, and freshly distilled TMSCl (41.0 mmol, 1.1 equiv) was added. The reaction was allowed to stir at 22 °C for 2 hours. Upon completion, the mixture was quenched with water (50 mL) and extracted three times with ethyl acetate. The combined organic layers were dried with Na₂SO₄, filtered, and concentrated under reduced pressure. The crude product was purified by column chromatography (hexanes/ethyl acetate, 10:1) to afford the desired product as a white solid in 97% yield.

**^1^H NMR (600 MHz, CDCl_3_)** δ 1.18 (s, 12H), 1.17 (s, 12H), 0.81 (tt, J = 8.4, 5.5 Hz, 1H), 0.42 (td, J = 5.7, 3.9 Hz, 2H), 0.40 – 0.32 (m, 2H), 0.12 (s, 9H).

**^13^C NMR (151 MHz, CDCl_3_)** δ 82.19, 24.90, 24.65, 9.64, 4.83, -0.56.

**^11^B NMR (128 MHz, CDCl_3_)** δ 33.99.

**HRMS (ESI+) [M+H]+** N/A

**■Synthesis of Trifluoromethyl Arenes**

**2-(3,5-dimethoxyphenyl)-5-(trifluoromethyl)pyridine (S13): (Suzuki Cross Coupling reaction procedure A)**^4^ In air, a round-bottom flask was charged sequentially with phenylboronic acid (1.5 eq), potassium carbonate (0.5 mmol), palladium acetate (2 mol %), and 2-bromo-5-(trifluoromethyl)pyridine (1.33 mmol, 1.0 equiv). An ethanol–water mixture (3:1 v/v, 4 mL total) was then added. The reaction mixture was allowed to stir at 80 °C for 60 minutes. Upon completion, the reaction was quenched with saturated brine and extracted with ethyl acetate. The combined organic layers were dried over Na₂SO₄, filtered, and concentrated under reduced pressure. The crude residue was purified by column chromatography to afford the desired product **S13** as a white solid in 88% yield.

**^1^H NMR (600 MHz, CDCl_3_)** δ 8.96 (s, 1H), 7.99 (dd, *J* = 8.3, 1.9 Hz, 1H), 7.83 (d, *J* = 8.3 Hz, 1H), 7.22 (d, *J* = 2.3 Hz, 2H), 6.61 (t, *J* = 2.3 Hz, 1H), 3.91 (s, 6H).

**^13^C NMR (151 MHz, CDCl_3_)** δ 161.29, 160.36, 146.48 (d, *J* = 4.0 Hz), 139.99, 133.91 (d, *J* = 3.5 Hz), 125.91 (d, *J* = 155.9 Hz), 125.18, 123.78 (t, *J* = 288.9 Hz), 120.61 (d, *J* = 274.9 Hz), 102.27, 55.57.

**^19^F NMR (376 MHz, CDCl_3_)** δ -62.24.

**HRMS (ESI+) [M+H]+** calcd for C_14_H_13_F_3_NO_2_^+^: 284.0893; found: 284.0889

**2-(allyloxy)-5-(trifluoromethyl)pyridine (x): (S_n_2 reaction procedure B)** To a stirred suspension of potassium carbonate (2.50 equiv) in acetone (0.2 M), allyl bromide (2.00 equiv) and 5-(trifluoromethyl)pyridin-2-ol (1.84 mmol, 1.00 equiv) were added at room temperature. The reaction mixture was then allowed to stir under reflux for 12 hours, with progress monitored by TLC. Upon complete conversion of the starting material, the mixture was filtered through Celite and concentrated under reduced pressure. The crude residue was purified by flash column chromatography (hexane/ethyl acetate 10:1 to 5:1) to afford the desired aryl allyl ether **S14** in 98% yield.

**^1^H NMR (600 MHz, CDCl_3_)** δ 7.64 (s, 1H), 7.44 (dd, *J* = 9.6, 2.6 Hz, 1H), 6.64 (d, *J* = 9.6 Hz, 1H), 6.14 – 5.81 (m, 1H), 5.34 (d, *J* = 10.3 Hz, 1H), 5.26 (d, *J* = 17.1 Hz, 1H), 4.58 (d, *J* = 6.0 Hz, 2H).

**^13^C NMR (151 MHz, CDCl_3_)** δ 161.63, 136.57 (q, *J* = 5.3 Hz), 135.05 (d, *J* = 2.3 Hz), 131.45, 123.30 (q, *J* = 269.7 Hz), 121.56, 119.85, 109.79 (q, *J* = 34.9 Hz), 51.49.

**^19^F NMR (376 MHz, CDCl_3_)** δ -62.40.

**HRMS (ESI+) [M+H]+** calcd for C_9_H_9_F_3_NO^+^: 204.0631; found: 204.0630

**2-(cyclopropylethynyl)-5-(trifluoromethyl)pyridine (S15): (Sonogashira reaction procedure C)**^5^ To a solution of 2-bromo-5-trifluoromethyl pyridine (1 eq), PdCl_2_(PPh_3_)_2_ (5 mol %) and CuI (10 mol %) in Et_3_N (0.5 eq) and MeCN (0.4M) was added alkyne (1.2 eq) under inert atmosphere. The mixture was allowed to stir for 3 h at room temperature, then concentrated in vacuo. The residue was diluted with Et_2_O, and insoluble materials were removed by filtration. The filtrate was concentrated in vacuo, and the crude was purified by silica gel flash column chromatography (hexane/ethyl acetate = 10/1) to give **S15** in 74% yield as a white powder.

**^1^H NMR (600 MHz, CDCl_3_)** δ 8.79 (s, 1H), 7.87 – 7.79 (m, 1H), 7.46 (d, *J* = 8.2 Hz, 1H), 1.57 – 1.48 (m, 1H), 1.07 – 0.84 (m, 4H).

**^13^C NMR (151 MHz, CDCl_3_)** δ 147.26, 146.65 (q, *J* = 3.9 Hz), 133.20 (q, *J* = 3.7 Hz), 126.25, 126.13, 125.09 – 124.23 (m), 121.62 (t, *J* = 272.2 Hz), 97.60, 74.98, 9.05, 0.17.

**^19^F NMR (377 MHz, CDCl_3_)** δ -62.47.

**HRMS (ESI+) [M+H]+** calcd for C_11_H_9_F_3_N^+^: 212.0682; found: 212.0680

**1-methyl-5-(5-(trifluoromethyl) pyridine-2-yl)-1H-indole (S16):** CF_3_ arene was synthesized according to the cross-coupling procedure A to give the title compound as a yellow solid in 85% yield.

**^1^H NMR (600 MHz, CDCl_3_)** δ 8.95 (s, 1H), 8.42 – 8.29 (m, 1H), 7.97 (ddd, *J* = 13.0, 8.6, 1.8 Hz, 2H), 7.91 (d, *J* = 8.4 Hz, 1H), 7.45 (d, *J* = 8.6 Hz, 1H), 7.14 (d, *J* = 3.0 Hz, 1H), 6.62 (d, *J* = 2.9 Hz, 1H), 3.87 (s, 3H).

**^13^C NMR (151 MHz, CDCl_3_**) δ 162.02, 146.43 (d, *J* = 4.1 Hz), 137.82, 135.06 – 133.32 (m), 130.02, 129.52, 128.90, 124.94 (q, *J* = 270.1 Hz), 123.65 (q, *J* = 32.9 Hz), 121.04, 120.45, 119.57, 109.69, 102.17, 33.03. Peaks overlapping.

**^19^F NMR (565 MHz, CDCl_3_)** δ -62.08.

**HRMS (ESI+) [M+H]+** calcd for C_15_H_12_F_3_N_2_^+^: 277.0947; found: 277.0952

**3-(3-(methylthio)phenyl)-5-(trifluoromethyl)pyridine (S17):** CF_3_ arene was synthesized according to the cross-coupling procedure A.

**^1^H NMR (400 MHz, CDCl_3_)** δ 8.99 (d, *J* = 2.1 Hz, 1H), 8.87 (s, 1H), 8.07 (s, 1H), 7.50 – 7.37 (m, 2H), 7.37 – 7.29 (m, 2H), 2.54 (s, 3H).

**^13^C NMR (101 MHz, CDCl_3_)** δ 151.40 (d, *J* = 1.7 Hz), 145.34 (q, *J* = 4.0 Hz), 140.22, 136.90, 136.41, 131.37 (q, *J* = 3.6 Hz), 129.73, 126.78 (q, *J* = 32.8 Hz), 126.64, 125.12, 123.93, 123.49 (q, *J* = 272.7 Hz), 15.72.

**^19^F NMR (377 MHz, CDCl_3_)** δ -62.36.

**HRMS (ESI+) [M+H]+** calcd for C_13_H_11_F_3_NS^+^: 270.0559; found: 270.0558

**tert-butyl 4-(5-(trifluoromethyl)pyridin-3-yl)piperazine-1-carboxylate (S18): (procedure D):** In a round bottom flask, sodium tert-butoxide (1.5), tert-butyl piperazine-1-carboxylate (1.2 eq), xanthphos (3 mol %) and Pd(dba)_2_ (1 mol %) were added into a suspension of 3-bromo-5-trifluoromethyl-pyridine (1 eq) in toluene (0.11M). The resulting mixture was allowed to stir at 90 °C for 16 hours. The reaction mixture was concentrated under vacuum. After purification by flash chromatography (hexane/ethyl acetate 1:1) the title compound was obtained as a white solid in 51% yield.

**^1^H NMR (400 MHz, CDCl_3_)** δ 8.41 (d, *J* = 4.7 Hz, 1H), 8.30 (d, *J* = 4.6 Hz, 1H), 7.27 (s, 1H), 3.57 (t, *J* = 5.2 Hz, 4H), 3.21 (t, *J* = 5.2 Hz, 4H),1.44 (s, 9H).

**^13^C NMR (101 MHz, CDCl_3_)** δ 154.49, 146.39, 141.54, 136.85 (q, *J* = 4.2 Hz), 126.63 (q, *J* = 32.2 Hz), 123.60 (q, *J* = 272.8 Hz), 118.49, 118.47, 80.22, 47.97, 28.33.

**^19^F NMR (377 MHz, CDCl_3_)** δ -62.53 (d, *J* = 2.1 Hz).

**HRMS (ESI+) [M+H]+** calcd for C_15_H_21_F_3_N_3_O_2_^+^: 332.1580; found: 332.1578

**3,5-bis(trifluoromethyl)phenol (S19): (Procedure E)**^6^**:** To an oven-dried 25 mL round-bottom flask was added 3,5-bis (trifluoromethyl)phenol (2.2 mmol, 1.0 equiv), anhydrous potassium carbonate (4.0 equiv), acetone (0.1 M) and iodomethane (3.0 equiv). The mixture was stirred under reflux with a condenser for 15 h. After cooling to rt, the solvent was removed by rotary evaporator to a crude residue that was dissolved in water and extracted with dichloromethane. The combined organic layer was dried over anhydrous Na_2_SO_4_, filtered and concentrated. The product was purified by flash column chromatography (ethyl acetate: hexane = 1: 10) as a colorless oil (90% yield). All the spectra matched the literature data.^6^

**5-(trifluoromethyl)-2-(4-(trifluoromethyl)phenyl)pyridine (S20):** CF_3_ arene was synthesized according to the Cross Coupling procedure A. All spectra matched the reported data^4^.

**2-(benzyloxy)-5-(trifluoromethyl)pyridine (S21):** CF_3_ arene was synthetized according to the S_n_2 procedure B. All spectra matched the reported data^7^.

**2-(1H-imidazol-1-yl)-5-(trifluoromethyl)pyridine (S22):** **(S_N_Ar reaction procedure F)** In air, a round-bottom flask was charged with 2-fluoro-5-trifluoromethylpyridine (1 eq), potassium carbonate (3 eq), and pyrazole (1 eq). DMSO (0.2M) was then added at room temperature. The SnAr SNAr reaction was carried out at 110 °C for 18 hours with magnetic stirring. After completion of the reaction, saturated LiCl solution (15 mL) was added to remove the DMSO. The reaction mixture was extracted with ethyl acetate, and the organic phases were combined. The combined organic layers were concentrated, and the desired product was obtained in 85% yield after purification by column chromatography(chromatography (hexane: ethyl acetate 1:1). All spectra matched the reported data^8^.

**2-phenyl-5-(trifluoromethyl)pyridine (S23):** CF_3_ arene was synthesized according to the Cross-Coupling procedure A (used 1,4 dioxane/H_2_O( 3/1 v)). All spectra matched the reported data. ^9^

**2-(3,5-bis(trifluoromethyl)phenyl)pyridine (S24)**: CF_3_ arene was synthesized according to the Cross-Coupling procedure A. All spectra matched the reported data.^10^

**3,5-bis(trifluoromethyl)-1,1'-biphenyl (S25):** CF_3_ arene was synthesized according to the Cross-Coupling procedure A. All spectra matched the reported data.^11^

**2-(phenylethynyl)-5-(trifluoromethyl)pyridine (S26):** CF_3_ arene was synthesized according to the Sonogashira cross-coupling procedure C.^5^ All spectra matched the reported data^12^.

**2-phenoxy-5-(trifluoromethyl)pyridine (S27):** CF_3_ arene was synthesized according to the S_N_Ar procedure F. All spectra matched the reported data^13^.

**2-(4-methoxyphenoxy)-5-(trifluoromethyl)pyridine (S28):** CF_3_ arene was synthetized according to the S_N_Ar procedure F. All spectra matched the reported data.^14^

**3-phenyl-5-(trifluoromethyl)pyridine (S29):** CF_3_ arene was synthesized according to the cross-coupling procedure A. All spectra matched the reported data. ^15^

**1-allyl-5-(trifluoromethyl)pyridin-2(1H)-one (S30): (Procedure G):** Synthesis of pyridines via an O to N alkyl migration)^16^: In an 8 mL vial, allyloxy-5-(trifluoromethyl)pyridine(1.84 mmol, 1.0 eq) and lithium iodide (0.5 eq) were added. The vial was sealed, and the reaction mixture was allowed to stir at 100 °C for 20 hours. Upon completion, the crude mixture was filtered through a Celite plug using ethyl acetate, and the filtrate was dried over Na₂SO₄. The solvent was removed under reduced pressure. The crude residue was purified by column chromatography on silica gel to afford the desired product **S30** as a colorless oil in 79% yield. All spectra matched the reported data.^17^

**1-benzyl-5-(trifluoromethyl)pyridin-2(1H)-one (S31):** CF_3_ arene was synthesized according to the procedure G. All spectra matched the data^18^

**diethyl (3-(trifluoromethyl)phenyl)phosphonate (S32) (Procedure H):** A flame-dried nitrogen-filled Schlenk flask was charged with palladium acetate (2 mol %) and triphenylphosphine (2 mol %). Aryl bromide (1 eq), diisopropylethylamine (6 eq), diethyl phosphite (5 eq), and dry ethanol (0.1 M) were then added, and the reaction mixture was allowed to stir under reflux for 48h. The reaction mixture was then allowed to cool to room temperature and diluted by the addition of diethyl ether. The reaction mixture was washed with hydrochloric acid (1M). The resulting aqueous layer was extracted with diethyl ether, and the diethyl ether extracts were washed with aqueous sodium hydroxide (2.5M), brine. The combined organic layers were then dried with MgSO_4_, filtered, and concentrated under reduced pressure to afford a yellow oil. The crude product was purified by column chromatography (ethyl acetate: petrol 2:1). All the spectra matched the literature data. ^19^

**N,N-dimethyl-3-(trifluoromethyl)benzenesulfonamide (S33): (Procedure L)**^6^ Dimethylamine (2M in THF, 1.5 equiv) was slowly added to a solution of 3-(trifluoromethyl)benzenesulfonyl chloride (5.0 mmol, 1.0 equiv) and triethylamine (1.8 equiv) in  DCM (0.3M) at 0 °C. After allowing the mixture to stir for 15 min, the reaction flask was removed from the ice bath and allowed to stir for another 1 h at room temperature. The reaction mixture was poured into water (20 mL) and extracted with DCM. The combined organic layer was dried over anhydrous Na_2_SO_4_ filtered and concentrated. The product was isolated by flash column chromatography (ethyl acetate:hexane = 1:5) as a white solid in 97% yield. All the spectra matched the literature data.^6^

**N-(3,5-bis(trifluoromethyl)benzyl)-N-methyl-3-phenyl-3-(4-(trifluoromethyl)phenoxy)propan-1-amine (S34): (procedure J)**^20^ To an 8 ml vial, N-methyl-3-phenyl-3-(4-(trifluoromethyl)phenoxy)propan-1-amine (fluoxetine, 1.45 mmol, 1.0 equiv), and anhydrous potassium carbonate (2.2 equiv) were added and dissolved in anhydrous acetonitrile (8 mL). Aryl bromide (1-(bromomethyl)-3,5-bis(trifluoromethyl)benzene) was then added to the reaction mixture via syringe (1.1 equiv) at rt. The vial was sealed with a hard cap and allowed to stir for 12 h at 70°C. The reaction mixture was then allowed to cool to room temperature and quenched with water, and extracted with diethyl ether. The combined organic phase was dried over anhydrous Na_2_SO_4_, filtered, and concentrated. The product was isolated by flash column chromatography (diethyl ether/hexane= 1:6) as a yellow oil (40% yield). All spectra matched the literature data.^21^

**N-(3,5-bis(trifluoromethyl)benzyl)-N-(1-(naphthalen-1-yl)ethyl)-3-(3-(trifluoromethyl)phenyl)propan-1-amine (S35):** The CF3 arene was synthesized according to procedure J. All spectra matched the literature data. ^20^

**■General Procedure for Synthesis of Aryl Substituted TMS 1,1-diborons**:

Bis[(pinacolato)boryl]methylzinc halide (1-ZnBr) was prepared according to a reported procedure.⁷ In a nitrogen-filled glovebox, bis[(pinacolato)boryl]methyllithium (1-Li, 1.0 equiv), anhydrous zinc halide (1.0 equiv), and THF (1.0 M) were added to an oven-dried 4-dram vial equipped with a Teflon-coated magnetic stir bar. The vial was sealed with a PTFE/silicone-lined septum cap and allowed to stir at room temperature for 30 minutes. The resulting 1-ZnBr solution was used directly in subsequent cross-coupling reactions without further purification.

Prepared according to the procedure.^22^ In a nitrogen-filled glove box, aryl bromide (1.0 equiv), Pd_2_(dba)_3_ (1.0 mol%), P(o-tolyl)_3_ (2.0 mol%), and THF (0.25 M) were added to an 8 ml vial equipped with a Teflon-coated magnetic stir bar. To this mixture, a solution of bis[(pinacolato) boryl] methylzinc bromide (1-ZnBr, 1.5 equiv) in THF (1.0 M) prepared by S3 was sequentially added. The vial was removed from the glove box and allowed to stir at 60 °C for 3 h. The reaction mixture was filtered through Celite and concentrated under reduced pressure. The crude mixture was purified by column chromatography on silica gel or recrystallization to give the corresponding product. These proton and carbon NMRs matched with the literature data.^22^

- **General procedure for Z-Fluoroalkenyl Boronates**

**Procedure A:** In a nitrogen-filled glovebox, an 8 mL vial equipped with a magnetic stir bar was charged with cesium fluoride (CsF, 3.0 equiv), 18-crown-6 (40 mol % ), and TMS-DBM (3.0 equiv). DMF (0.4M) was then added, followed by the addition of the trifluoromethyl arene (1.0 equiv, 0.300 mmol; actual amount varied as noted for specific substrates), either as a solid or liquid. The vial was sealed with a hard cap and secured with electrical tape before being removed from the glovebox. The reaction mixture was allowed to stir at 22 °C for 24 h. Upon completion, the reaction was quenched with water and extracted with ethyl acetate. The organic layers were then washed with saturated aqueous LiCl to remove DMF. The combined organic layers were dried over Na₂SO₄, filtered, and concentrated under reduced pressure. The crude product was analyzed by NMR using mesitylene (5 µL) as an internal standard to determine yield. The product was then purified by column chromatography using the appropriate mobile phase.

**Procedure B:** In a nitrogen-filled glovebox, an 8 mL vial equipped with a magnetic stir bar was charged with lithium (LTMP, 3.0 equiv) and Bis[(pinacolato)boryl]methane (DBM, 3.0 equiv). Anhydrous DME (0.5 mL) was then added, followed by the trifluoromethyl arene (1.0 equiv, 0.100 mmol), added as either a solid or liquid. The vial was sealed and removed from the glovebox. The reaction mixture was allowed to stir at 50 °C for 24 hours. Upon completion, the reaction was allowed to cool to room temperature, quenched with water, and extracted with ethyl acetate. The combined organic layers were dried with Na₂SO₄, filtered, and concentrated under reduced pressure. The crude product was analyzed by ¹H NMR using mesitylene (5 µL) as an internal standard to determine yield. The product was then purified by column chromatography using the appropriate mobile phase.

**(Z)-5-(1-fluoro-2-(4,4,5,5-tetramethyl-1,3,2-dioxaborolan-2-yl)vinyl)-2-methoxypyridine (8a)**

General procedure A was followed using 2-methoxy-5-trifluoromethylpyridine (0.30 mmol). The product was purified by silica gel column chromatography (hexane/ethyl acetate 30:1 to 3:1). Any excess pinacol was removed by azeotropic distillation with methanol, yielding the compound **8a** as a colorless oil (66 mg, 79% yield) with a 98:2 Z:E.

**^1^H NMR (600 MHz, CDCl_3_)** δ 8.45 (d, *J* = 2.5, 1H), 7.75 (dd, *J* = 8.4, 2.6 Hz, 1H), 6.74 (d, *J* = 8.7, 1H), 5.17 (d, *J* = 54.2 Hz, 1H), 3.96 (s, 3H), 1.32 (s, 12H).

**^13^C NMR (151 MHz, CDCl_3_**) δ 168.16 (d, *J* = 262.4 Hz), 165.15, 144.81 (d, *J* = 8.3 Hz), 135.63 (d, *J* = 7.0 Hz), 121.99, 110.93 (d, *J* = 2.1 Hz), 83.45, 53.80, 24.73.

**^19^F NMR (377 MHz, CDCl_3_)** δ -90.68 (d, *J* = 54.1 Hz).

**HRMS (ESI+) [M+H]+** calcd for C_14_H_20_BFNO_3_^+^; 280.1515 found: 280.1510

**(Z)-2-bromo-5-(1-fluoro-2-(4,4,5,5-tetramethyl-1,3,2-dioxaborolan-2-yl)vinyl)pyridine (8b)**

General procedure A was followed using 2-bromo-5-trifluoromethylpyridine (0.30 mmol). The product was purified by silica gel column chromatography (hexane/ethyl acetate 30:1 to 10:1). Any excess pinacol was removed by azeotropic distillation with methanol, yielding the compound **8b** as a colorless oil (85 mg, 86% yield) with a 98:2 Z:E.

**^1^H NMR (600 MHz, CDCl_3_)** δ 8.61 (d, *J* = 2.5 Hz, 1H), 7.71 (dd, *J* = 8.4, 2.6 Hz, 1H), 7.51 (d, *J* = 8.4 Hz, 1H), 5.37 (d, *J* = 53.4 Hz, 1H), 1.32 (s, 12H).

**^13^C NMR (151 MHz, CDCl_3_)** δ 166.25 (d, *J* = 263.1 Hz), 147.19 (d, *J* = 7.6 Hz), 143.58, 134.85 (d, *J* = 6.5 Hz), 127.98 (d, *J* = 1.9 Hz), 127.71 (d, *J* = 31.9 Hz), 83.78, 24.81.

**^19^F NMR (377 MHz, CDCl_3_)** δ -91.82 (d, *J* = 53.4 Hz).

**^11^B NMR (128 MHz, CDCl_3_)** δ 29.86.

**HRMS (ESI+) [M+H]+** calcd for C_13_H_17_BBrFNO_2_^+^: 328.0514; found: 328.0509

**(Z)-2-chloro-5-(1-fluoro-2-(4,4,5,5-tetramethyl-1,3,2-dioxaborolan-2-yl)vinyl)pyridine(8c)**

General procedure A was followed using 2-chloro-5-trifluoromethylpyridine (0.30 mmol). The product was purified by silica gel column chromatography (hexane/ethyl acetate 30:1 to 3:1). Any excess pinacol was removed by azeotropic distillation with methanol, yielding the compound **8c** as a colorless oil (78 mg, 92% yield) with a 98:2 Z:E

**^1^H NMR (600 MHz, CDCl_3_**) δ 8.63 (s, 1H), 7.82 (d, *J* = 8.4 Hz, 1H), 7.35 (d, *J* = 8.4 Hz, 1H), 5.36 (d, *J* = 53.4, 1H), 1.32 (s, 12H).

**^13^C NMR (151 MHz, CDCl_3_)** δ 166.21 (d, *J* = 262.9 Hz), 152.80, 146.87 (d, *J* = 7.6 Hz), 135.22 (d, *J* = 6.5 Hz), 127.38 (d, *J* = 31.8 Hz), 124.17 (d, *J* = 2.0 Hz), 83.77, 24.80.

**^19^F NMR (377 MHz, CDCl_3_)** δ -91.65 (d, *J* = 53.4).

**HRMS (ESI+) [M+H]+** calcd for C_13_H_17_BClFNO_2_^+^; 284.1019; found: 284.1029

**(Z)-2-(2-(4-bromo-3-chlorophenyl)-2-fluorovinyl)-4,4,5,5-tetramethyl-1,3,2-dioxaborolane (8d)**

General procedure B was followed using 2-bromo-3-chloro-5-trifluoromethylpyridine (0.30 mmol). The product was purified by silica gel column chromatography (hexane/ethyl acetate 30:1 to 3:1). Any excess pinacol was removed by azeotropic distillation with methanol, yielding the compound **8d** as a colorless oil (61 mg, 56% yield) with a 98:2 Z:E

**^1^H NMR (600 MHz, CDCl_3_)** δ 8.49 (s, 1H), 7.87 (s, 1H), 5.40 (d, *J* = 53.1 Hz, 1H), 1.31 (s, 12H).

**^13^C NMR (151 MHz, CDCl_3_)** δ 164.73 (d, *J* = 263.5 Hz), 144.31 (d, *J* = 7.3 Hz), 142.87, 134.26 (d, *J* = 6.9 Hz), 133.62 (d, *J* = 2.2 Hz), 128.94 (d, *J* = 32.1 Hz), 83.89, 24.78.

**^19^F NMR (377 MHz, CDCl_3_)** δ -91.85 (d, *J* = 53.3 Hz).

**HRMS (ESI+) [M+H]+** calcd for C_14_H_17_BBrClFO_2_^+^; 361.0172; found: 361.0161

**tert-butyl (Z)-4-(5-(1-fluoro-2-(4,4,5,5-tetramethyl-1,3,2-dioxaborolan-2-yl)vinyl)pyridin-3-yl) piperazine-1-carboxylate (8e)**

General procedure B was followed using tert-butyl 4-(5-(trifluoromethyl)pyridin-3-yl)piperazine-1-carboxylate (0.30 mmol). The product was purified by silica gel column chromatography (hexane/ethyl acetate 30:1 to 3:1). Any excess pinacol was removed by azeotropic distillation with methanol, yielding the compound **8e** as a colorless oil (81 mg, 62% yield) with a 98:2 Z:E

**^1^H NMR (600 MHz, CDCl_3_)** δ 8.36 (br s, 2H), 7.33 (s, 1H), 5.35 (d, *J* = 53.9 Hz, 1H), 3.59 (t, *J* = 5.4 Hz, 1H), 3.19 (t, *J* = 5.4 Hz, 1H).1.48 (s, 9H), 1.33 (s, 12H).

**^13^C NMR (151 MHz, CDCl_3_)** δ 167.65 (d, *J* = 264.0 Hz), 154.58, 146.51 (d, *J* = 1.7 Hz), 139.92, 138.15 (d, *J* = 7.2 Hz), 128.45 (d, *J* = 30.4 Hz), 128.33, 118.82 (d, *J* = 7.1 Hz), 83.62, 48.36, 28.41, 24.81.

**^19^F NMR (377 MHz, CDCl_3_)** δ -90.70 (d, *J* = 53.9 Hz).

**HRMS (ESI+) [M+H]+** calcd for C_22_H_34_BFN_3_O_4_^+^; 434.2621; found: 434.2632

**(Z)-3-(1-fluoro-2-(4,4,5,5-tetramethyl-1,3,2-dioxaborolan-2-yl)vinyl)-5-phenylpyridine (8f)**

General procedure B was followed using 3-phenyl-5-trifluoromethylpyridine (0.30 mmol). The product was purified by silica gel column chromatography (hexane/ethyl acetate 30:1 to 3:1). Any excess pinacol was removed by azeotropic distillation with methanol, yielding the compound **(8f)** as a colorless oil (46 mg, 47% yield) with a 98:2 Z:E

**^1^H NMR (600 MHz, CDCl_3_)** δ 8.82 (t, *J* = 2.6 Hz, 2H), 8.04 (t, *J* = 2.2 Hz, 1H), 7.58 – 7.53 (m, 2H), 7.51 – 7.45 (m, 2H), 7.45 – 7.39 (m, 1H), 5.45 (d, *J* = 53.7 Hz, 1H), 1.33 (s, 12H).

**^13^C NMR (151 MHz, CDCl_3_)** δ 167.26 (d, *J* = 263.9 Hz), 149.24, 145.39 (d, *J* = 7.5 Hz), 136.97, 130.94 (d, *J* = 6.7 Hz), 129.24, 128.55, 127.17, 83.69, 75.01, 24.86.

**^19^F NMR (377 MHz, CDCl_3_)** δ -91.29 (d, *J* = 53.6 Hz).

**HRMS (ESI+) [M+H]+** calcd for C_19_H_22_BFNO_2_^+^; 326.1722; found: 326.1724

**(Z)-5-(1-fluoro-2-(4,4,5,5-tetramethyl-1,3,2-dioxaborolan-2-yl)vinyl)-2-phenoxypyridine (8g)**

General procedure A was followed using 2-phenoxy-5-trifluoromethylpyridine (0.30 mmol). The product was purified by silica gel column chromatography (hexane/ethyl acetate 30:1 to 3:1). Any excess pinacol was removed by azeotropic distillation with methanol, yielding the compound **8g** as a colorless oil (92 mg, 90% yield) with a 98:2 Z:E

**^1^H NMR (600 MHz, CDCl_3_)** δ 8.46 (d, *J* = 2.5 Hz, 1H), 7.87 (dd, *J* = 8.7, 2.5 Hz, 1H), 7.45 – 7.38 (m, 2H), 7.25 – 7.21 (m, 1H), 7.18 – 7.12 (m, 2H), 6.92 (dd, *J* = 8.7, 0.8 Hz, 1H), 5.22 (d, *J* = 54.0 Hz, 1H), 1.32 (s, 12H).

**^13^C NMR (151 MHz, CDCl_3_)** δ 167.57 (d, *J* = 262.6 Hz), 164.77, 153.52, 145.48 (d, *J* = 8.2 Hz), 136.45 (d, *J* = 6.9 Hz), 129.78, 125.24, 123.46 (d, *J* = 31.5 Hz), 121.43, 111.12 (d, *J* = 2.0 Hz), 83.54, 24.82.

**^19^F NMR (377 MHz, CDCl_3_)** δ -90.73 (d, *J* = 54.1 Hz).

**HRMS (ESI+) [M+H]+** calcd for C_19_H_22_BFNO_3_^+^; 342.1671; found: 342.1672

**(Z)-5-(1-fluoro-2-(4,4,5,5-tetramethyl-1,3,2-dioxaborolan-2-yl)vinyl)-2-(4-methoxyphenoxy) pyridine (8h)**

General procedure A was followed using 2-(4-methoxyphenoxy)-5-(trifluoromethyl)pyridine (0.30 mmol). The product was purified by silica gel column chromatography (hexane/ethyl acetate 30:1 to 3:1). Any excess pinacol was removed by azeotropic distillation with methanol, yielding the compound **8h** as a colorless oil (97 mg, 87% yield) with a 98:2 Z:E.

**^1^H NMR (600 MHz, CDCl_3_)** δ 8.44 (d, *J* = 2.5 Hz, 1H), 7.84 (dd, *J* = 8.7, 2.5 Hz, 1H), 7.06 (d, *J* = 9.0 Hz, 2H), 6.92 (d, *J* = 9.0 Hz, 2H), 6.88 (d, *J* = 8.7 Hz, 1H), 5.20 (d, *J* = 54.0 Hz, 1H), 3.80 (s, 3H), 1.31 (s, 11H).

**^13^C NMR (151 MHz, CDCl_3_)** δ 167.64 (d, *J* = 262.5 Hz), 165.20, 156.89, 146.80, 145.43 (d, *J* = 8.1 Hz), 136.36 (d, *J* = 6.9 Hz), 123.18 (d, *J* = 31.2 Hz), 122.45, 114.81, 110.82 (d, *J* = 1.8 Hz), 83.51, 55.57, 24.81.

**^19^F NMR (377 MHz, CDCl_3_)** δ -90.74 (d, *J* = 54.1 Hz).

**HRMS (ESI+) [M+H]+** calcd for C_20_H_24_BFNO_4_^+^; 372.1777; found: 372.1774

**(Z)-3-(1-fluoro-2-(4,4,5,5-tetramethyl-1,3,2-dioxaborolan-2-yl)vinyl)-5-(3-(methylthio) phenyl) pyridine (8i)**

General procedure B was followed using 3-(3-(methylthio)phenyl)-5-(trifluoromethyl)pyridine (0.30 mmol). The product was purified by silica gel column chromatography (hexane/ethyl acetate 30:1 to 3:1). Any excess pinacol was removed by azeotropic distillation with methanol, yielding the compound **8i** as a colorless oil (68 mg, 61% yield) with a 98:2 Z:E

**^1^H NMR (600 MHz, CDCl_3_)** δ 8.88 (s, 1H), 8.84 (s, 1H), 8.05 (t, *J* = 2.2 Hz, 1H), 7.46 – 7.40 (m, 2H), 7.37 – 7.31 (m, 2H), 5.48 (d, *J* = 53.6 Hz, 1H), 1.36 (s, 12H).

**^13^C NMR (151 MHz, CDCl_3_)** δ 167.20 (d, *J* = 263.8 Hz), 149.28, 145.70 (d, *J* = 7.3 Hz), 139.95, 137.74, 136.10, 130.94 (d, *J* = 6.9 Hz), 129.60, 128.43 (d, *J* = 31.0 Hz), 126.41, 125.00, 123.87, 83.72, 83.03, 24.73.

**^19^F NMR (377 MHz, CDCl_3_)** δ -91.32 (d, *J* = 53.6 Hz).

**HRMS (ESI+) [M+H]+** calcd for C_20_H_24_BFNO_2_S^+^; 372.1599; found: 372.1610

**(Z)-5-(1-fluoro-2-(4,4,5,5-tetramethyl-1,3,2-dioxaborolan-2-yl)vinyl)-2-(1H-imidazol-1-yl)pyridine (8j)**

General procedure A was followed using 2-imidazole-5-trifluoromethylpyridine (0.30 mmol). The product was purified by silica gel column chromatography (hexane/acetone 30:1 to 3:1). Any excess pinacol was removed by azeotropic distillation with methanol, yielding the compound **8j** as a colorless oil in 69% (65 mg) 98:2 Z:E.

**^1^H NMR (400 MHz, CDCl_3_)** δ 8.75 (d, *J* = 2.2 Hz, 1H), 8.41 (s, 1H), 8.03 (dd, *J* = 8.6, 2.3 Hz, 1H), 7.68 (s, 1H), 7.40 (d, *J* = 8.6 Hz, 1H), 7.28 (s, 1H), 5.40 (d, *J* = 53.7 Hz, 1H), 1.36 (s, 12H).

**^13^C NMR (151 MHz, CDCl_3_)** δ 166.43 (d, *J* = 262.8 Hz), 149.65, 146.51 (d, *J* = 8.0 Hz), 142.06, 135.78 (d, *J* = 6.9 Hz), 135.08, 131.09, 126.79 (d, *J* = 32.2 Hz), 116.06, 111.71, 83.77, 24.83.

**^19^F NMR (377 MHz, CDCl_3_)** δ -91.72 (d, *J* = 53.6 Hz).

**HRMS (ESI+) [M+H]+** calcd for C_16_H_20_BFN_3_O_2_^+^; 316.1627; found: 316.1627

**(Z)-5-(1-fluoro-2-(4,4,5,5-tetramethyl-1,3,2-dioxaborolan-2-yl)vinyl)-2-phenylpyridine (8k)**

General procedure A was followed using 2-phenyl-5-trifluoromethylpyridine (0.30 mmol). The product was purified by silica gel column chromatography (hexane/ethyl acetate 30:1 to 3:1). Any excess pinacol was removed by azeotropic distillation with methanol, yielding the compound **8k** as a colorless oil (81 mg, 83% yield) with a 98:2 Z:E.

**^1^H NMR (600 MHz, CDCl_3_)** δ 8.94 (d, *J* = 2.3 Hz, 1H), 8.03 – 7.98 (m, 2H), 7.93 (dd, *J* = 8.4, 2.1 Hz, 1H), 7.75 (d, *J* = 8.4 Hz, 1H), 7.50 – 7.45 (m, 2H), 7.45 – 7.41 (m, 1H), 5.41 (d, *J* = 53.9 Hz, 1H), 1.33 (s, 12H).

**^13^C NMR (151 MHz, CDCl_3_)** δ 167.61 (d, *J* = 263.1 Hz), 158.42, 146.86 (d, *J* = 7.6 Hz), 138.37, 133.42 (d, *J* = 6.7 Hz), 129.61, 128.87, 127.02, 126.59 (d, *J* = 31.1 Hz), 119.94 (d, *J* = 1.8 Hz), 83.63, 24.85 (d, *J* = 4.6 Hz).

**^19^F NMR (377 MHz, CDCl_3_)** δ -91.57 (d, *J* = 54.0 Hz).

**HRMS (ESI+) [M+H]+** calcd for C_19_H_22_BFNO_2_^+^; 326.1722found: 326.1724

**(Z)-5-(1-fluoro-2-(4,4,5,5-tetramethyl-1,3,2-dioxaborolan-2-yl)vinyl)-2-(4-(trifluoromethyl)phenyl) pyridine (8l)**

General procedure A was followed using 2-(4-trifluoromethyl(phenyl)-5-trifluoromethylpyridine (0.30 mmol). The product was purified by silica gel column chromatography (hexane/ethyl acetate 30:1 to 3:1). Any excess pinacol was removed by azeotropic distillation with methanol, yielding the compound **8l** as a colorless oil (100 mg, 89% yield) with a 98:2 Z:E.

**^1^H NMR (400 MHz, CDCl_3_**) δ 8.51 (s, 1H), 7.67 (d, *J* = 8.4 Hz, 2H), 7.51 (d, *J* = 8.4 Hz, 1H), 7.33 (d, *J* = 8.4 Hz, 1H), 7.27 (d, *J* = 8.4 Hz, 2H), 4.99 (d, *J* = 53.7 Hz, 1H), 0.89 (s, 12H).

**^13^C NMR (151 MHz, CDCl_3_)** δ 167.15 (d, *J* = 262.9 Hz), 156.68, 147.02 (d, *J* = 7.6 Hz), 141.62, 133.57 (d, *J* = 6.8 Hz), 131.29 (q, *J* = 32.5 Hz), 127.45 (d, *J* = 31.3 Hz), 127.27, 125.79 (q, *J* = 3.7 Hz), 120.92, 120.26, 83.70, 24.81.

**^19^F NMR (377 MHz, CDCl_3_)** δ -62.67, -91.81 (d, *J* = 53.6 Hz).

**HRMS (ESI+) [M+H]+** calcd for C_20_H_21_BF_4_NO_2_^+^; 394.1596; found: 394.1609

**(Z)-2-(3,5-dimethoxyphenyl)-5-(1-fluoro-2-(4,4,5,5-tetramethyl-1,3,2-dioxaborolan-2-yl) vinyl) pyridine (8m)**

General procedure A was followed using 2-(3,5-dimethoxyphenyl)-5-(trifluoromethyl)pyridine (0.30 mmol). The product was purified by silica gel column chromatography (hexane/ethyl acetate 30:1 to 3:1). Any excess pinacol was removed by azeotropic distillation with methanol, yielding the compound **8m** as a white solid in 48% (55 mg) 98:2 Z:E.

**^1^H NMR (600 MHz, CDCl_3_)** δ 8.94 (d, *J* = 1.8 Hz, 1H), 7.93 (dd, *J* = 8.4, 2.3 Hz, 1H), 7.73 (d, *J* = 8.4 Hz, 1H), 7.19 (d, *J* = 2.2 Hz, 2H), 6.55 (t, *J* = 2.2 Hz, 1H), 5.41 (d, *J* = 54.0 Hz, 1H), 3.88 (s, 6H), 1.34 (s, 12H).

**^13^C NMR (151 MHz, CDCl_3_)** δ 168.44, 166.70, 161.21, 158.12, 146.71 (d, *J* = 7.7 Hz), 140.48, 133.40 (d, *J* = 6.8 Hz), 126.84 (d, *J* = 31.1 Hz), 120.17, 104.99, 102.06, 83.65, 55.55, 24.84.

**^19^F NMR (377 MHz, CDCl_3_**) δ -91.61 (d, *J* = 53.8 Hz).

**HRMS (ESI+) [M+H]+** calcd for C_21_H_26_BFNO_4_^+^; 386.1933; found: 386.1946

**(Z)-2-(cyclopropylethynyl)-5-(1-fluoro-2-(4,4,5,5-tetramethyl-1,3,2-dioxaborolan-2-yl)vinyl)pyridine (8n)**

General procedure A was followed using 2-(cyclopropylethynyl)-5-(trifluoromethyl)pyridine (0.30 mmol). The product was purified by silica gel column chromatography (hexane/ethyl acetate 30:1 to 3:1). Any excess pinacol was removed by azeotropic distillation with methanol, yielding the compound **8n** as a colorless oil (65 mg, 69% yield) with a 98:2 Z:E

**^1^H NMR (400 MHz, CDCl_3_)** δ 8.74 (s, 1H), 7.75 (d, *J* = 8.4 Hz, 1H), 7.31 (d, *J* = 8.2 Hz, 1H), 5.34 (d, *J* = 53.7 Hz, 1H), 1.42 – 1.49 (m, 1H), 1.29 (s, 12H), 0.91 – 0.84 (m, 4H).

**^13^C NMR (101 MHz, CDCl_3_)** δ 167.00 (d, *J* = 263.0 Hz), 146.85 (d, *J* = 7.7 Hz), 144.84, 132.49 (d, *J* = 6.8 Hz), 126.47 (d, *J* = 31.3 Hz), 126.23 (d, *J* = 2.0 Hz), 96.83, 83.64, 75.44, 60.40, 53.44, 24.77.

**^19^F NMR (377 MHz, CDCl_3_)** δ -92.09 (d, *J* = 53.7 Hz).

**HRMS (ESI+) [M+H]+** calcd for C_18_H_22_BFNO_2_^+^; 314.1722; found: 314.1721

**(Z)-5-(1-fluoro-2-(4,4,5,5-tetramethyl-1,3,2-dioxaborolan-2-yl)vinyl)-2-(phenylethynyl)pyridine (8o)**

General procedure A was followed using 2-(phenylethynyl)-5-(trifluoromethyl)pyridine (0.30 mmol). The product was purified by silica gel column chromatography (hexane/ethyl acetate 30:1 to 3:1). Any excess pinacol was removed by azeotropic distillation with methanol, yielding the compound **8o** as a colorless oil (83 mg, 79% yield) with a 98:2 Z:E

**^1^H NMR (600 MHz, CDCl_3_)** δ 8.87 (d, *J* = 2.3 Hz, 1H), 7.86 (dd, *J* = 8.2, 2.3 Hz, 1H), 7.63 – 7.57 (m, 2H), 7.55 – 7.51 (m, 1H), 7.41 – 7.33 (m, 3H), 5.42 (d, *J* = 53.6 Hz, 1H), 1.33 (s, 12H).

**^13^C NMR (151 MHz, CDCl_3_)** δ 166.86 (d, *J* = 263.2 Hz), 147.19 (d, *J* = 7.6 Hz), 144.41, 132.60 (d, *J* = 6.6 Hz), 132.16, 129.30, 128.47, 127.07 (d, *J* = 31.3 Hz), 126.67 (d, *J* = 1.9 Hz), 121.95, 91.37, 88.40, 83.72, 24.82.

**^19^F NMR (377 MHz, CDCl_3_)** δ -94.73 (d, *J* = 53.7 Hz).

**HRMS (ESI+) [M+H]+** calcd for C_21_H_22_BFNO_2_^+^; 350.1722; found: 350.1724

**(Z)-1-allyl-5-(1-fluoro-2-(4,4,5,5-tetramethyl-1,3,2-dioxaborolan-2-yl)vinyl)pyridin-2(1H)-one (8p)**

General procedure A was followed using 1-allyl-5-(trifluoromethyl)pyridin-2(1H)-one (0.10 mmol) and 0.2M as final concentration. The product was purified by silica gel column chromatography (DCM/MeOH 30:1 to 10:1). Any excess pinacol was removed by azeotropic distillation with methanol, yielding the compound **8p** as a while solid in 57% (17.5 mg) 98:2 Z:E.

**^1^H NMR (600 MHz, CDCl_3_)** δ 7.61 (d, *J* = 2.6 Hz, 1H), 7.47 (d, *J* = 9.6 Hz, 1H), 6.59 (d, *J* = 9.6 Hz, 1H), 6.03 – 5.82 (m, 1H), 5.30 (d, *J* = 10.3 Hz, 1H), 5.22 (d, *J* = 17.1 Hz, 1H), 4.97 (d, *J* = 54.9 Hz, 1H), 4.57 (d, *J* = 5.9 Hz, 2H), 1.31 (s, 12H).

**^13^C NMR (151 MHz, CDCl_3_)** δ 166.45 (d, *J* = 259.9 Hz), 161.70, 136.07 (d, *J* = 6.5 Hz), 135.29 (d, *J* = 9.7 Hz), 131.85, 121.12 (d, *J* = 2.3 Hz), 120.60 (d, *J* = 23.4 Hz), 119.37, 83.47, 51.38, 24.80.

**^19^F NMR (377 MHz, CDCl_3_)** δ -70.61 (d, *J* = 30.9 Hz).

**HRMS (ESI+) [M+H]+** calcd for C_16_H_22_BFNO_3_^+^; 306.1671; found: 306.1671

**(Z)-1-benzyl-5-(1-fluoro-2-(4,4,5,5-tetramethyl-1,3,2-dioxaborolan-2-yl)vinyl)pyridin-2(1H)-one (8q)**

General procedure A was followed using 1-benzyl-5-(trifluoromethyl)pyridin-2(1H)-one (0.10 mmol) and 0.2M as final concentration. The product was purified by silica gel column chromatography (DCM/MeOH 30:1 to 10:1). Any excess pinacol was removed by azeotropic distillation with methanol, yielding the compound 8q as a while solid in 50% (17.7 mg), 98:2 Z:E.

**^1^H NMR (600 MHz, CDCl_3_)** δ 7.61 (d, *J* = 2.6 Hz, 1H), 7.51 – 7.42 (m, 1H), 7.40 – 7.27 (m, 5H), 6.61 (dd, *J* = 9.6, 1.3 Hz, 1H), 5.13 (s, 2H), 4.93 (d, *J* = 54.7 Hz, 1H), 1.29 (s, 12H).

**^13^C NMR (151 MHz, CDCl_3_)** δ 166.42 (d, *J* = 259.9 Hz), 161.96, 135.97 (d, *J* = 6.5 Hz), 135.52, 135.37 (d, *J* = 9.8 Hz), 129.08, 128.42, 121.24, 111.97, 83.45, 52.37, 24.78.

**^19^F NMR (564 MHz, CDCl_3_)** δ -93.99. (decoupling)

**HRMS (ESI+) [M+H]+** calcd for C_20_H_24_BFNO_3_^+^; 356.1828; found: 356.1830

**(Z)-2-(2-fluoro-2-(3-(trifluoromethyl)phenyl)vinyl)-4,4,5,5-tetramethyl-1,3,2-dioxaborolane (8r)**

General procedure A was followed using 1,2-bis-(trifluoromethyl)benzene (0.30 mmol). The product was purified by silica gel column chromatography (hexane/ethyl acetate 30:1 to 3:1). Any excess pinacol was removed by azeotropic distillation with methanol, yielding the compound **8r** as a colorless oil (73 mg, 77% yield) with a 98:2 Z:E.

**^1^H NMR (600 MHz, CDCl_3_**) δ 7.89 (d, *J* = 2.1 Hz, 1H), 7.83 – 7.79 (m, 1H), 7.67 (ddt, *J* = 7.8, 1.8, 0.9 Hz, 1H), 7.57 – 7.51 (m, 1H), 5.42 (d, *J* = 53.4 Hz, 1H), 1.36 (s, 12H).

**^13^C NMR (151 MHz, CDCl_3_)** δ 168.12 (d, *J* = 263.4 Hz), 133.25 (d, *J* = 31.8 Hz), 131.60 – 130.74 (m), 129.20 (d, *J* = 2.0 Hz), 128.46 (d, *J* = 7.1 Hz), 126.70 (q, *J* = 3.7 Hz), 123.75 (d, *J* = 272.4 Hz), 122.27 (dq, *J* = 8.0, 3.9 Hz), 83.63, 24.82.

**^19^F NMR (377 MHz, CDCl_3_)** δ -62.97, -90.86 (d, *J* = 53.5 Hz).

**HRMS (ESI+) [M+Na]+** calcd for C_15_H_17_BF_4_NaO_2_^+^; 339.1150; found: 339.1161

**(Z)-3-(1-fluoro-2-(4,4,5,5-tetramethyl-1,3,2-dioxaborolan-2-yl)vinyl)benzonitrile (8s)**

General procedure A was followed using 2-(4-trifluoromethyl(phenyl)-5-trifluoromethylpyridine (0.30 mmol). The product was purified by silica gel column chromatography (hexane/ethyl acetate 30:1 to 3:1). Any excess pinacol was removed by azeotropic distillation with methanol, yielding the compound **8s** as a colorless oil (79 mg, 96% yield) with a 98:2 Z:E

**^1^H NMR (600 MHz, CDCl_3_)** δ 7.88 (t, *J* = 1.8 Hz, 1H), 7.82 (dt, *J* = 8.1, 1.5 Hz, 1H), 7.67 (dt, *J* = 7.7, 1.4 Hz, 1H), 7.51 (t, *J* = 7.8, 1H), 5.38 (d, *J* = 53.1 Hz, 1H), 1.33 (s, 12H).

**^13^C NMR (151 MHz, CDCl_3_)** δ 167.15 (d, *J* = 263.3 Hz), 133.74 (d, *J* = 32.2 Hz), 133.30, 129.55 (d, *J* = 2.0 Hz), 129.39 (d, *J* = 7.1 Hz), 128.88 (d, *J* = 7.6 Hz), 118.19, 113.14 (d, *J* = 2.2 Hz), 83.75, 24.81.

**^19^F NMR (377 MHz, CDCl_3_)** δ -91.52 (d, *J* = 53.0 Hz).

**HRMS (ESI+) [M+Na]+** calcd for C_15_H_17_BFNNaO_2_^+^; 296.1229; found: 296.1237

**(Z)-3-(1-fluoro-2-(4,4,5,5-tetramethyl-1,3,2-dioxaborolan-2-yl)vinyl)-N,N-dimethylbenzene sulfonamide (8t)**

General procedure A was followed using N,N-dimethyl-3-(trifluoromethyl)benzenesulfonamide (0.30 mmol). The product was purified by silica gel column chromatography (hexane/ethyl acetate 30:1 to 3:1). Any excess pinacol was removed by azeotropic distillation with methanol, yielding the compound **8t** as a colorless oil (79 mg, 74% yield) with a 98:2 Z:E.

**^1^H NMR (600 MHz, CDCl_3_**) δ 7.95 (s, 1H), 7.81 – 7.70 (m, 2H), 7.58 – 7.47 (m, 1H), 5.39 (d, *J* = 53.3 Hz, 1H), 2.66 (s, 6H), 1.28 (s, 12H).

**^13^C NMR (151 MHz, CDCl_3_)** δ 172.67 (d, *J* = 263.8 Hz), 141.33, 138.55 (d, *J* = 31.8 Hz), 134.47, 134.19 (d, *J* = 7.0 Hz), 133.92, 129.40 (d, *J* = 5.9 Hz), 88.68, 80.02, 42.85, 29.79.

**^19^F NMR (377 MHz, CDCl_3_)** δ -90.80 (d, *J* = 53.0 Hz).

**HRMS (ESI+) [M+H]+** calcd for C_16_H_24_BFNO_4_S^+^; 356.1498; found: 356.1498

**Z)-2-(3-(1-fluoro-2-(4,4,5,5-tetramethyl-1,3,2-dioxaborolan-2-yl)vinyl)-5-(trifluoromethyl) phenyl) pyridine (8u)**

General procedure A was followed using 2-(3,5-bis(trifluoromethyl)phenyl)pyridine (0.30 mmol). The product was purified by silica gel column chromatography (hexane/ethyl acetate 30:1 to 3:1). Any excess pinacol was removed by azeotropic distillation with methanol, yielding the compound **8u** as white solid (80 mg, 68% yield) 98:2 Z:E.

**^1^H NMR (600 MHz, CDCl_3_)** δ 8.69 (d, *J* = 4.7 Hz, 1H), 8.39 (s, 1H), 8.29 (s, 1H), 7.86 (s, 1H), 7.79 – 7.75 (m, 1H), 7.75 – 7.71 (m, 1H), 7.30 – 7.27 (m, 1H), 5.49 (d, *J* = 53.4 Hz, 1H), 1.31 (s, 12H).

**^13^C NMR (151 MHz, CDCl_3_)** δ 167.92 (d, *J* = 263.7 Hz), 154.98, 149.96, 140.68 (d, *J* = 2.0 Hz), 137.14, 133.84 (d, *J* = 31.7 Hz), 131.76 (q, *J* = 32.7 Hz), 126.83 (d, *J* = 7.1 Hz), 125.21 (d, *J* = 3.8 Hz), 123.21, 122.84, 122.35 (p, *J* = 3.8 Hz), 120.57, 83.62, 24.82.

**^19^F NMR (377 MHz, CDCl_3_)** δ -62.82, -90.63 (d, *J* = 53.4 Hz).

**HRMS (ESI+) [M+H]+** calcd for C_20_H_21_BF_4_NO_2_^+^; 394.1596; found: 394.1599

**(Z)-(3-(1-fluoro-2-(4,4,5,5-tetramethyl-1,3,2-dioxaborolan-2-diethyl)vinyl)phenyl)phosphonate (8v)**

General procedure A was followed using diethyl (3-(trifluoromethyl)phenyl)phosphonate (0.30 mmol). The product was purified by silica gel column chromatography (hexane/ethyl acetate 30:1 to 3:1). Any excess pinacol was removed by azeotropic distillation with methanol, yielding the compound **8v** as a colorless oil (50 mg, 46% yield) with a 98:2 Z:E

**^1^H NMR (600 MHz, CDCl_3_)** δ 8.03 (d, *J* = 14.1 Hz, 1H), 7.85 (dd, *J* = 13.0, 7.5 Hz, 1H), 7.78 (d, *J* = 7.9 Hz, 1H), 7.49 (td, *J* = 7.7, 4.1 Hz, 1H), 5.39 (d, *J* = 53.5 Hz, 1H), 4.18 – 4.06 (m, 2H), 1.33 (s, 12H), 1.33 – 1.27 (m, 6H).

**^13^C NMR (151 MHz, CDCl_3_)** δ 168.61 (d, *J* = 263.4 Hz), 133.32 (d, *J* = 9.9 Hz), 132.77 (dd, *J* = 31.4, 15.2 Hz), 129.93, 128.98 (dd, *J* = 7.2, 3.2 Hz), 128.84 (dd, *J* = 15.2, 1.9 Hz), 128.61 (dd, *J* = 11.0, 7.6 Hz), 83.56, 62.33 (d, *J* = 5.5 Hz), 24.82, 16.34 (d, *J* = 6.5 Hz).

**^19^F NMR (377 MHz, CDCl_3_)** δ -90.66 (d, *J* = 53.6 Hz).

**HRMS (ESI+) [M+H]+** calcd for C_18_H_28_BFO_5_P^+^; 385.1746; found: 385.1726

**(Z)-2-(2-fluoro-2-(5-(trifluoromethyl)-[1,1'-biphenyl]-3-yl)vinyl)-4,4,5,5-tetramethyl-1,3,2-dioxaborolane (8w)**

General procedure A was followed using 3,5-bis(trifluoromethyl)-1,1'-biphenyl (0.30 mmol). The product was purified by silica gel column chromatography (hexane/ethyl acetate 30:1 to 3:1). Any excess pinacol was removed by azeotropic distillation with methanol, yielding the compound **(8w)** as white solid (31 mg, 32% yield) 98:2 Z:E.

**^1^H NMR (400 MHz, CDCl_3_)** δ 8.02 (s, 1H), 7.91 – 7.83 (m, 2H), 7.63 – 7.58 (m, 2H), 7.50 (ddd, *J* = 8.4, 6.9, 1.1 Hz, 2H), 7.47 – 7.41 (m, 1H), 5.49 (d, *J* = 53.3 Hz, 1H), 1.36 (s, 12H).

**^13^C NMR (101 MHz, CDCl_3_)** δ 168.09 (d, *J* = 263.7 Hz), 142.65 (d, *J* = 1.9 Hz), 139.03, 133.85 (d, *J* = 31.7 Hz), 131.74 (qd, *J* = 32.6, 2.1 Hz), 129.14, 128.45, 127.16, 123.77 (q, *J* = 272.5 Hz), 120.93 (dt, *J* = 7.6, 3.9 Hz), 83.64, 82.40 (d, *J* = 50.5 Hz), 53.41, 24.97.

**^19^F NMR (377 MHz, CDCl_3_)** δ -62.85, -90.56 (d, *J* = 53.3 Hz).

**HRMS (ESI+):** Mass spec couldn’t be obtained.

**2,2'-((1Z,1'Z)-(5-methoxy-1,3-phenylene)bis(2-fluoroethene-2,1-diyl))bis(4,4,5,5-tetramethyl-1,3,2-dioxaborolane) (8x)**

General procedure A was followed using 3,5-bis(trifluoromethyl)-1,1'-biphenyl (0.30 mmol). The product was purified by silica gel column chromatography (hexane/ethyl acetate 30:1 to 3:1). Any excess pinacol was removed by azeotropic distillation with methanol, yielding the compound **8x** as white solid (35 mg, 26% yield) 98:2 Z:E.

**^1^H NMR (400 MHz, CDCl_3_**) δ 7.47 (d, *J* = 1.5 Hz, 1H), 7.15 (d, *J* = 1.5 Hz, 2H), 5.31 (d, *J* = 53.5 Hz, 2H), 3.81 (s, 3H), 1.32 (s, 24H).

**^13^C NMR (101 MHz, CDCl_3_)** δ 168.86 (d, *J* = 263.9 Hz), 159.75, 134.10 (dd, *J* = 31.2, 1.9 Hz), 115.08 (t, *J* = 7.7 Hz), 112.61 (d, *J* = 7.6 Hz), 83.53, 55.44, 24.81.

**^19^F NMR (377 MHz, CDCl_3_)** δ -89.68 (d, *J* = 53.4 Hz).

**HRMS (ESI+) [M+Na]+** calcd for C_23_H_32_B_2_F_2_NaO_5_^+^; 471.2296; found: 471.2296

- **General Procedure for Synthesis of Z- and E-Tetrasubstituted Fluoroalkenyl Boronates**

**Procedure A**: In a nitrogen-filled glovebox, an 8 mL vial equipped with a magnetic stir bar was charged with cesium fluoride (3.0 equiv), 18-crown-6 (40 mol %), and alkyl-substituted TMS-DBM (3.0 equiv). Anhydrous DMF (0.5 mL, (0.2M)) was added, followed by the trifluoromethyl arene (1.0 equiv, 0.10 mmol), introduced as either a solid or liquid. The vial was sealed with a hard cap before being removed from the glovebox. The reaction mixture was allowed to stir at 22 °C for 24 hours. Upon completion, the reaction was quenched with water and extracted with ethyl acetate. The organic layers were then washed with saturated aqueous LiCl to remove DMF. The combined organic layers were dried over Na₂SO₄, filtered, and concentrated under reduced pressure. The crude product was analyzed by ¹H NMR using mesitylene (5 µL) as an internal standard to determine yield. The product was then purified by column chromatography using the appropriate mobile phase.

**(E)-2-(1-fluoro-1-(3-(trifluoromethyl)phenyl)prop-1-en-2-yl)-4,4,5,5-tetramethyl-1,3,2-dioxaborolane (9a)**

General procedure A was followed using Bis-(1,3-trifluoromethyl)benzene. The product was purified by silica gel column chromatography (hexane/ethyl acetate 30:1 to 10:1). Any excess pinacol was removed by azeotropic distillation with methanol, yielding the compound **9a** as a colorless oil in 58% yield (19.2 mg) 98:2 E:Z. The NMR yield of the crude mixture was 68% yield in 98:2 E:Z

**^1^H NMR (600 MHz, CDCl3):** δ 7.76 (s, 1H), 7.67 (d, *J* = 8.1 Hz, 1H), 7.63 (d, *J* = 7.8 Hz, 1H), 7.38 (t, *J* = 8.6 Hz, 1H), 1.84 (d, *J* = 2.2 Hz, 3H), 1.34 (s, 12H).

**^13^C NMR (MHz, CDCl3)**: δ 162.11 (d, *J* = 250.7 Hz), 133.48 (d, *J* = 33.1 Hz), 132.16, 131.57 (d, *J* = 3.7 Hz), 130.67 (q, *J* = 32.6 Hz), 128.68, 127.51, 125.82, 123.96, 83.65, 24.83, 14.40 (d, *J* = 9.1 Hz).

**^19^F NMR (565 MHz, CDCl3):** δ -62.85, -82.97.

**HRMS (ESI+) [M+Na]+** calcd for C_16_H_19_BF_4_NaO_2_^+^; 353.1306, found: 353.1317

**(E)-3-(1-fluoro-2-(4,4,5,5-tetramethyl-1,3,2-dioxaborolan-2-yl)prop-1-en-1-yl)benzonitrile (9b)**

General procedure A was followed using 3-trifluoromethyl benzonitrile. The product was purified by silica gel column chromatography (hexane/ethyl acetate 30:1 to 10:1). Any excess pinacol was removed by azeotropic distillation with methanol, yielding the compound **9b** as a colorless oil in 63% yield (18.2 mg) as a single isomer E(>98/2 E/Z). The NMR yield of the crude mixture was 90% in 81:19 E:Z.

**Major isomer: ^1^H NMR (400 MHz, CDCl_3_)** δ 7.77 (s, 1H), 7.72 (d, *J* = 7.9 Hz, 1H), 7.68 – 7.62 (m, 1H), 7.52 (t, *J* = 7.8 Hz, 1H), 1.83 (d, *J* = 2.3 Hz, 3H), 1.34 (s, 12H).

**Major isomer: ^13^C NMR (101 MHz, CDCl_3_)** δ 161.10 (d, *J* = 250.6 Hz), 133.98 (d, *J* = 33.5 Hz), 132.55, 132.52, 131.93 (d, *J* = 5.1 Hz), 129.14, 118.35, 112.58, 83.77, 24.83, 14.48 (d, *J* = 9.0 Hz).

**Major isomer: ^19^F NMR (377 MHz, CDCl_3_)** δ -84.02.

**Minor isomer: ^1^H NMR (600 MHz, CDCl_3_):** δ 7.82 (s, 1H), 7.72 (d, *J* = 7.9 Hz, 1H), 7.63 (d, *J* = 7.7 Hz, 1H), 7.45 (t, *J* = 7.8 Hz, 1H), 1.88 (d, *J* = 2.8 Hz, 3H), 1.23 (s, 12H).

**Minor isomer: ^13^C NMR (MHz, CDCl_3_):** δ 161.28 (d, *J* = 261.5 Hz), 134.79 (d, *J* = 32.6 Hz), 132.45 (d, *J* = 5.2 Hz), 132.40, 132.30 (d, *J* = 4.1 Hz), 128.54, 118.43, 111.80, 83.90, 24.64, 12.58 (d, *J* = 12.2 Hz).

**Minor isomer: ^19^F NMR (565 MHz, CDCl_3_)**: δ -81.61.

**HRMS (ESI+) [M+Na]+** calcd for C_16_H_19_BFNNaO_2_^+^; 310.1385, found: 310.1395

**(E)-6-(5-(1-fluoro-2-(4,4,5,5-tetramethyl-1,3,2-dioxaborolan-2-yl)prop-1-en-1-yl)pyridin-2-yl)-1-methyl-1H-indole (9c)**

General Procedure A was followed using 2-indolyl-5-(trifluoromethyl)pyridine as the substrate. The crude product was purified by column chromatography on silica gel (hexanes/ethyl acetate, gradient from 30:1 to 10:1). Excess pinacol was removed by azeotropic distillation with methanol, affording compound **9c** as a colorless oil in 57% isolated yield (22.3 mg) as a single isomer E (>98/2 E/Z). The NMR yield of the crude mixture was 75% in 89:11 E:Z.

**^1^H NMR (600 MHz, CDCl_3_):** δ 8.99 – 8.72 (m, 1H), 8.31 (d, *J* = 1.3 Hz, 1H), 7.95 (dd, *J* = 8.6, 1.7 Hz, 1H), 7.91 – 7.77 (m, 2H), 7.41 (d, *J* = 8.6 Hz, 1H), 7.09 (d, *J* = 3.1 Hz, 1H), 6.58 (d, *J* = 3.0 Hz, 1H), 3.83 (s, 3H), 1.92 (d, *J* = 2.2 Hz, 3H), 1.35 (s, 12H).

**^13^C NMR (MHz, CDCl_3_)**: δ 161.83 (d, *J* = 250.0 Hz), 158.84, 149.21 (d, *J* = 5.9 Hz), 137.57, 136.07 (d, *J* = 4.4 Hz), 130.18, 129.78, 128.89, 126.02 (d, *J* = 32.6 Hz), 120.94, 120.05, 119.36, 109.56, 102.05, 83.60, 33.01, 24.86, 14.52 (d, *J* = 9.2 Hz).

**^19^F NMR (565 MHz, CDCl_3_)** δ -84.97.

**HRMS (ESI+) [M+H]+** calcd for C_23_H_27_BFN_2_O_2_^+^; 393.2144, found: 393.2148

**(E)-5-(1-fluoro-2-(4,4,5,5-tetramethyl-1,3,2-dioxaborolan-2-yl)prop-1-en-1-yl)-2-methoxypyridine (9d)**

General procedure A was followed using 2-methoxy-5-trifluoromethyl pyridine. The product was purified by silica gel column chromatography (hexane/ethyl acetate 30:1 to 10:1). Any excess pinacol was removed by azeotropic distillation with methanol, yielding the compound **9d** as a colorless oil in 53% yield(15.5 mg) as a single isomer E(>98/2 E/Z). The NMR yield of the crude product was 71%, and >98:2 E:Z.

**^1^H NMR (600 MHz, CDCl_3_)** δ 8.33 (d, *J* = 1.1 Hz, 1H), 7.69 (dd, *J* = 8.7, 2.4 Hz, 1H), 6.76 (d, *J* = 8.7 Hz, 1H), 3.96 (s, 3H), 1.83 (d, *J* = 2.2 Hz, 3H), 1.33 (s, 12H).

**^13^C NMR (151 MHz, CDCl_3_)** δ 169.13, 166.84 (d, *J* = 250.1 Hz), 152.34 (d, *J* = 6.5 Hz), 143.25, 127.34 (d, *J* = 34.3 Hz), 115.52, 87.91, 58.65, 29.83, 19.46 (d, *J* = 9.2 Hz).

**^19^F NMR (377 MHz, CDCl_3_)** δ -83.33.

**HRMS (ESI+) [M+H]+** calcd for C_15_H_22_BFNO_3_^+^; 294.1671, found: 294.1673

**(E)-5-(1-fluoro-2-(4,4,5,5-tetramethyl-1,3,2-dioxaborolan-2-yl)prop-1-en-1-yl)-2-phenylpyridine (9e)**

General procedure A was followed using 2-phenyl-5-trifluoromethyl pyridine. The product was purified by silica gel column chromatography (hexane/ethyl acetate 30:1 to 10:1). Any excess pinacol was removed by azeotropic distillation with methanol, yielding the compound **9e** as a colorless oil in 77% yield (20.2 mg) as a single isomer E. The NMR yield of the crude mixture was 95% and 85:15 E:Z.

**^1^H NMR (600 MHz, CDCl_3_):** δ 8.85 (d, *J* = 1.8 Hz, 1H), 8.03 (d, *J* = 7.4 Hz, 2H), 7.88 (dd, *J* = 8.3, 2.2 Hz, 1H), 7.78 (d, *J* = 8.3 Hz, 1H), 7.49 (t, *J* = 7.5 Hz, 2H), 7.46 – 7.39 (m, 1H), 1.91 (d, *J* = 2.2 Hz, 3H), 1.35 (s, 9H).

**^13^C NMR (MHz, CDCl_3_)**: δ 161.33 (d, *J* = 249.9 Hz), 157.46, 149.35 (d, *J* = 5.8 Hz), 138.60, 136.26 (d, *J* = 4.4 Hz), 129.46, 128.86, 127.02, 119.72, 83.67, 24.85, 14.49 (d, *J* = 9.2 Hz).

**^19^F NMR (565 MHz, CDCl_3_)**: δ -85.19.

**HRMS (ESI+) [M+H]+** calcd for C_20_H_24_BFNO_2_^+^; 340.1879, found: 340.1863

**(E)-5-(1-fluoro-2-(4,4,5,5-tetramethyl-1,3,2-dioxaborolan-2-yl)prop-1-en-1-yl)-2-(1H-imidazol-1-yl)pyridine (9f)**

General procedure A was followed using 2-imidazol-5-trifluoromethyl pyridine. The product was purified by silica gel column chromatography (hexane/ethyl acetate 10:1 to 0:1). Any excess pinacol was removed by azeotropic distillation with methanol, yielding the compound **9f** as a colorless oil in 52% yield in 59:41 E:Z. The NMR yield of the crude mixture was 93% yield and 72:28 E:Z.

**Major isomer^1^H NMR (400 MHz, CDCl_3_)** δ 8.63 (s, 1H), 8.52 (d, *J* = 37.1 Hz, 1H), 8.39 (s, 1H), 7.95 (d, *J* = 8.4 Hz, 1H), 7.66 (s, 1H), 7.22 (s, 1H), 1.89 (s, 3H), 1.34 (s, 12H).

**Major isomer ^13^C NMR (101 MHz, CDCl_3_)** δ 158.89 (d, *J* = 19.7 Hz), 151.67, 149.00 (d, *J* = 5.8 Hz), 141.36, 138.59 (d, *J* = 4.4 Hz), 135.08, 130.89 (d, *J* = 18.5 Hz), 127.25 (d, *J* = 33.4 Hz), 116.10, 111.57, 83.94, 83.81, 24.86 (d, *J* = 3.9 Hz), 14.47 (d, *J* = 9.4 Hz).

**Minor isomer: ^19^F NMR (377 MHz, CDCl_3_)** δ -80.11.

**Major isomer: ^19^F NMR (377 MHz, CDCl_3_)** δ -85.45.

**HRMS (ESI+) [M+H]+** calcd for C_17_H_22_BFN_3_O_2_^+^; 330.1784, found: 330.1785

**(E)-3-(1-fluoro-2-(4,4,5,5-tetramethyl-1,3,2-dioxaborolan-2-yl)hex-1-en-4-yn-1-yl)benzonitrile (9g)**

General procedure A was followed using 3-(trifluoromethyl)benzonitrile. The product was purified by silica gel column chromatography (hexane/ethyl acetate 30:1 to 10:1). Any excess pinacol was removed by azeotropic distillation with methanol, yielding the compound **9g** as a colorless oil in 61% yield (19.8 mg) with a 79:21 Z:E. The NMR yield of the crude mixture was 98% NMR yield in 50:50 E:Z

**^1^H NMR (600 MHz, CDCl_3_)** δ 7.89 (s, 1H), 7.82 (d, *J* = 7.9 Hz, 1H), 7.69 (d, *J* = 7.8 Hz, 1H), 7.54 (t, *J* = 7.8 Hz, 1H), 2.95 (q, *J* = 2.4 Hz, 2H), 1.79 (t, *J* = 2.5 Hz, 3H), 1.35 (s, 12H).

**^13^C NMR (151 MHz, CDCl_3_)** δ 162.34 (d, *J* = 254.4 Hz), 133.39 (d, *J* = 32.6 Hz), 133.03, 132.77, 132.47 (d, *J* = 4.5 Hz), 131.87 (d, *J* = 4.8 Hz), 129.26, 128.69, 118.25, 112.77, 84.00, 24.74, 19.05 (d, *J* = 10.8 Hz), 3.59.

**Major isomer: ^19^F NMR (565 MHz, CDCl_3_)** δ -83.89.

**Minor isomer: ^19^F NMR (565 MHz, CDCl_3_)** δ -83.47.

**HRMS (ESI+):** Mass spec couldn’t be obtained

**(E)-3-(1-fluoro-3-phenyl-2-(4,4,5,5-tetramethyl-1,3,2-dioxaborolan-2-yl)prop-1-en-1-yl) benzonitrile (9h)**

General procedure A was followed using 3-(trifluoromethyl)benzonitrile. The product was purified by silica gel column chromatography (hexane/ethyl acetate 30:1 to 10:1). Any excess pinacol was removed by azeotropic distillation with methanol, yielding the compound **9h** as a colorless oil in 63% yield (23 mg) as a single isomer E. The NMR yield of the crude mixture was 79% in 83:17 E:Z.

**^1^H NMR (600 MHz, CDCl3)** δ 7.75 (s, 1H), 7.67 (t, *J* = 9.2 Hz, 2H), 7.49 (t, *J* = 7.8 Hz, 1H), 7.28 (d, *J* = 7.4 Hz, 2H), 7.20 – 7.12 (m, 3H), 3.56 (s, 2H), 1.17 (s, 12H).

**^13^C NMR (151 MHz, CDCl_3_)** δ 161.12 (d, *J* = 262.7 Hz), 140.34, 134.59, 132.76, 132.56 (d, *J* = 5.3 Hz), 132.49 (d, *J* = 4.3 Hz), 129.06, 128.77, 128.43, 126.23, 118.45, 112.06, 84.13, 33.42 (d, *J* = 10.5 Hz), 24.66.

**Major isomer: ^19^F NMR (377 MHz, CDCl_3_)** δ -82.03.

**Minor isomer: ^19^F NMR (377 MHz, CDCl_3_)** δ -79.43.

**HRMS (ESI+):** Mass spec couldn't be obtained

**(E)-5-(1-fluoro-2-(4,4,5,5-tetramethyl-1,3,2-dioxaborolan-2-yl)penta-1,4-dien-1-yl)-2-methoxypyridine (9i)**

General procedure A was followed using 2-methoxy-5-(trifluoromethyl)pyridine. The product was purified by silica gel column chromatography (hexane/ethyl acetate 30:1 to 10:1). Any excess pinacol was removed by azeotropic distillation with methanol, yielding the compound **9i** as a colorless oil in 78% yield (25 mg) in 74:26 E:Z. The NMR yield of the crude mixture was 85% in 69:31 E:Z.

**^1^H NMR (600 MHz, CDCl_3_)** δ 8.31 (s, 1H), 7.67 (dd, *J* = 8.7, 2.4 Hz, 1H), 6.75 (d, *J* = 8.6 Hz, 1H), 6.01 – 5.92 (m, 1H), 5.14 – 5.03 (m, 2H), 3.96 (s, 3H), 2.93 (d, *J* = 5.4 Hz, 2H), 1.31 (s, 12H).

**^13^C NMR (151 MHz, CDCl_3_)** δ 164.46, 162.58 (d, *J* = 251.9 Hz), 146.91 (d, *J* = 5.8 Hz), 138.12 (d, *J* = 3.7 Hz), 137.13 (d, *J* = 3.1 Hz), 121.90 (d, *J* = 33.0 Hz), 115.36, 110.58, 83.58, 53.68, 32.88 (d, *J* = 9.1 Hz), 24.75.

**Major isomer: ^19^F NMR (377 MHz, CDCl_3_)** δ -80.90.

**Minor isomer: ^19^F NMR (377 MHz, CDCl_3_)** δ -81.29.

**HRMS (ESI+) [M+H]+** calcd for C_17_H_24_BFNO_3_^+^; 320.1828, found: 320.1837

**(E)-2-bromo-5-(1-fluoro-5-methyl-2-(4,4,5,5-tetramethyl-1,3,2-dioxaborolan-2-yl)hexa-1,4-dien-1-yl)pyridine (9j)**

General procedure A was followed using 2-bromo-5-(trifluoromethyl) pyridine. The product was purified by silica gel column chromatography (hexane/ethyl acetate 30:1 to 10:1). Any excess pinacol was removed by azeotropic distillation with methanol, yielding the compound **9j** as a colorless oil in 60% yield (21 mg) in 87:13 E:Z. The NMR yield of the crude mixture was 78% and 69:31 E:Z.

**^1^H NMR (600 MHz, CDCl_3_)** δ 8.47 (d, *J* = 2.4 Hz, 1H), 7.62 (dd, *J* = 8.3, 2.4 Hz, 1H), 7.52 (d, *J* = 8.3 Hz, 1H), 5.08 (t, *J* = 6.7 Hz, 1H), 2.85 (d, *J* = 6.8 Hz, 2H), 1.86 – 1.63 (s, 3H), 1.57 (s, 3H), 1.32 (s, 12H).

**^13^C NMR (151 MHz, CDCl_3_)** δ 159.50 (d, *J* = 249.7 Hz), 149.67 (d, *J* = 5.2 Hz), 142.66, 137.77 (d, *J* = 3.6 Hz), 133.14, 127.95 (d, *J* = 33.3 Hz), 127.74, 121.99 (d, *J* = 2.7 Hz), 83.84, 27.68 (d, *J* = 8.8 Hz), 25.73, 24.74, 18.06.

**Major isomer: ^19^F NMR (377 MHz, CDCl_3_)** δ -85.80.

**Minor isomer: ^19^F NMR (377 MHz, CDCl_3_)** δ -84.90.

**HRMS (ESI+) [M+H]+** calcd for C_18_H_25_BBrFNO_2_^+^; 396.1140, found: 396.1151

**(E)-3-(3-(1,3-dioxolan-2-yl)-1-fluoro-2-(4,4,5,5-tetramethyl-1,3,2-dioxaborolan-2-yl)prop-1-en-1-yl)benzonitrile (9k)**

General procedure A was followed using 3-(trifluoromethyl)benzonitrile. The product was purified by silica gel column chromatography (hexane/ethyl acetate 30:1 to 10:1). Any excess pinacol was removed by azeotropic distillation with methanol, yielding the compound **9k** as a colorless oil in 77% yield (28 mg) in 87:13. The NMR yield of the crude mixture was 92% and 74:26 E:Z.

**^1^H NMR (400 MHz, CDCl_3_)** δ 7.90 (s, 1H), 7.79 (d, *J* = 7.9 Hz, 1H), 7.67 (d, *J* = 7.5 Hz, 1H), 7.48 (t, *J* = 7.8 Hz, 1H), 5.11 (s, 1H), 4.00 (s, 2H), 3.89 (s, 2H), 2.80 (s, 2H), 1.26 (s, 12H).

**^13^C NMR (101 MHz, CDCl_3_)** δ 161.54 (d, *J* = 263.8 Hz), 134.48 (d, *J* = 33.1 Hz), 132.67, 132.49 (d, *J* = 5.5 Hz), 132.42, 128.65, 118.33, 111.89, 103.47, 84.02, 64.98, 32.40 (d, *J* = 8.6 Hz), 24.69.

**Major isomer ^19^F NMR (377 MHz, CDCl_3_)** δ -82.20.

**Minor isomer ^19^F NMR (377 MHz, CDCl_3_)** δ -80.16.

**HRMS (ESI+):** Mass spec couldn’t be obtained

**(E)-3-(1-fluoro-2-(4,4,5,5-tetramethyl-1,3,2-dioxaborolan-2-yl)hept-1-en-1-yl)benzonitrile (9l)**

General procedure A was followed using 3-(trifluoromethyl)benzonitrile. The product was purified by silica gel column chromatography (hexane/ethyl acetate 30:1 to 10:1). Any excess pinacol was removed by azeotropic distillation with methanol, yielding the compound **9l** as a colorless oil in 87% yield (30 mg)with a 61:39 E:Z. The NMR yield of the crude mixture was 94% and 60:40 E:Z.

**^1^H NMR (600 MHz, CDCl_3_)** δ 7.84 (s, 1H), 7.75 (d, *J* = 6.8 Hz, 1H), 7.64 (d, *J* = 7.7 Hz, 1H), 7.46 (t, *J* = 7.8 Hz, 1H), 2.37 (t, *J* = 7.5 Hz, 2H), 1.52 – 1.42 (m, 2H), 1.32 (dd, *J* = 8.8, 4.9 Hz, 2H), 1.31 – 1.27 (m, 2H), 1.24 (s, 12H), 0.87 (t, *J* = 7.2 Hz, 3H).

**^13^C NMR (MHz, CDCl_3_)** ^13^C NMR (151 MHz, CDCl_3_) δ 160.45 (d, *J* = 249.7 Hz), 135.00 (d, *J* = 33.1 Hz), 132.53 (d, *J* = 2.1 Hz), 132.26, 128.59, 118.40, 112.63, 83.85, 32.56, 31.69, 29.51 (d, *J* = 1.3 Hz), 29.17, 27.46 (d, *J* = 9.5 Hz), 24.64, 22.61, 14.11.

**Major isomer:** **^19^F NMR (377 MHz, CDCl_3_)** δ -85.44

**Minor isomer: ^19^F NMR (377 MHz, CDCl_3_)** δ -83.08.

**HRMS (ESI+) [M+Na]+** calcd for C_21_H_29_BFNNaO_2_^+^; 380.2168, found: 380.2181.

- **Stereochemical Assignment by ¹⁹F NMR**

An amber 8 mL reaction vial equipped with a magnetic stir bar was charged with silver(I) fluoride (2.0 equiv). Methanol and water were added (5:4), followed by a solution of the alkenylboronate (1.0 equiv) in THF (Final 0.1 M). The vial was capped, sealed, and allowed to stir at 60 °C for 1.5 hours. After completion, the reaction mixture was allowed to cool to room temperature, diluted with diethyl ether, and passed through a short plug of silica gel. The filtrate was collected and concentrated under reduced pressure.^23^ The crude product was analyzed by ¹⁹F-NMR spectroscopy to determine the isomeric outcome, based on fluorine–hydrogen coupling constants.

**3-(1-fluoroprop-1-en-1-yl)benzonitrile (S40 and S41)**

**Major isomer E (S40): ^19^F NMR (565 MHz, CDCl_3_)** δ -104.85 (d, *J* = 21.7 Hz).

**Minor isomer Z (S41): ^19^F NMR (377 MHz, CDCl_3_)** δ -122.49 (d, *J* = 34.0 Hz).

Figure S1: ^19^F-NMR of Major isomer E (S40)

Figure S2: ^19^F-NMR of Minor isomer E in CDCl_3_ (S41)

**(E)-5-(1-fluoroprop-1-en-1-yl)-2-methoxypyridine (S42)**

**Single isomer E (S42): ^19^F NMR (377 MHz, CDCl_3_**) δ -103.92 (d, *J* = 21.9 Hz).

Figure S3: ^19^F-NMR of Major isomer E in CDCl_3_ (S42)

**3-(1-fluoro-2-(4,4,5,5-tetramethyl-1,3,2-dioxaborolan-2-yl)oct-1-en-1-yl)benzonitrile (S43 and S44)**

**Major isomer E (S43): ^19^F NMR (377 MHz, CDCl_3_)** δ -104.73 (d, *J* = 22.7 Hz)

**Minor isomer Z (S44): ^19^F NMR (377 MHz, CDCl_3_)** δ -122.25 (d, *J* = 36.9 Hz).

Figure S4: ^19^F-NMR of mixture of isomers in CDCl_3_ (S43 and S44)

- **General procedure for Synthesis of Z-Fluoroalkenes**

**Procedure A:** In a nitrogen-filled glovebox, an 8 mL vial equipped with a magnetic stir bar was charged with cesium fluoride (3.0 equiv) and aryl-substituted DBM (3.0 equiv; if different equivalents were used, they are noted under the specific substrate). Anhydrous DME (0.5 mL, (0.2M)) was added, followed by the trifluoromethyl arene (1.0 equiv, 0.100 mmol), introduced as either a solid or liquid. The vial was sealed before being removed from the glovebox. The reaction mixture was allowed to stir at 80 °C for 24 hours. After completion, the reaction was allowed to cool to room temperature, quenched with water, and extracted with water and ethyl acetate. The combined organic layers were dried over Na₂SO₄, filtered, and concentrated under reduced pressure. The crude product was purified by column chromatography using the appropriate mobile phase.

**(Z)-3-(1-fluoro-2-(4-methoxyphenyl)vinyl)benzonitrile (11a)**

General Procedure A was followed using 3-(trifluoromethyl)benzonitrile (0.10 mmol) and aryl-substituted DBM (1.1 equiv). The crude product was purified by column chromatography on silica gel (hexanes/ethyl acetate, gradient from 30:1 to 10:1). Excess pinacol was removed by azeotropic distillation with methanol, affording compound **11a** as a colorless oil in 58% yield (14.8 mg) with a 98:2 Z:E ratio.

**^1^H NMR (600 MHz, CDCl_3_)** δ 7.89 (s, 1H), 7.82 (d, *J* = 8.1 Hz, 1H), 7.70 – 7.54 (m, 3H), 7.51 (t, *J* = 7.8 Hz, 1H), 7.01 – 6.72 (m, 2H), 6.64 – 6.16 (m, 1H), 3.85 (s, 3H).

**^13^C NMR (151 MHz, CDCl_3_)** δ 159.43 (d, *J* = 3.1 Hz), 153.56 (d, *J* = 255.2 Hz), 134.48 (d, *J* = 29.0 Hz), 131.63, 130.67 (d, *J* = 8.1 Hz), 129.49 (d, *J* = 2.1 Hz), 127.82 (d, *J* = 7.2 Hz), 127.40 (d, *J* = 7.8 Hz), 125.44 (d, *J* = 3.2 Hz), 118.45, 114.21, 113.08 (d, *J* = 2.5 Hz), 107.66 (d, *J* = 10.3 Hz), 55.33.

**^19^F NMR (377 MHz, CDCl_3_)** δ -118.89 (d, *J* = 39.5 Hz).

**HRMS (ESI+) [M+H]+** calcd for C_16_H_12_FNO^•+^; 253.0897, found: 253.0898

**(Z)-2-bromo-5-(1-fluoro-2-phenylvinyl)pyridine (11b)**

General procedure A was followed using 2-bromo-5-(trifluoromethyl)pyridine. The product was purified by silica gel column chromatography (hexane/ethyl acetate 30:1 to 10:1). Any excess pinacol was removed by azeotropic distillation with methanol, yielding the compound **11b** as a colorless oil in 61% yield (17 mg) with a 98:2 Z:E.

**^1^H NMR (600 MHz, CDCl_3_)** δ 8.65 (d, *J* = 2.4 Hz, 1H), 7.76 (dd, *J* = 8.4, 2.5 Hz, 1H), 7.64 (d, *J* = 7.5 Hz, 2H), 7.53 (s, 1H), 7.40 (t, *J* = 7.7 Hz, 2H), 7.32 (t, *J* = 7.4 Hz, 1H), 6.36 (d, *J* = 39.4 Hz, 1H).

**^13^C NMR (151 MHz, CDCl_3_)** δ 153.74 (d, *J* = 258.1 Hz), 145.99 (d, *J* = 7.7 Hz), 142.15, 133.78 (d, *J* = 6.7 Hz), 132.60 (d, *J* = 3.2 Hz), 129.22 (d, *J* = 8.0 Hz), 128.78, 128.41, 128.23 (d, *J* = 2.6 Hz), 127.93 (d, *J* = 2.0 Hz), 108.32 (d, *J* = 9.4 Hz).

**^19^F NMR (377 MHz, CDCl_3_**) δ -116.43 (d, *J* = 39.3 Hz).

**HRMS (ESI+) [M+H]+** calc for C_13_H_10_BrFN^+^; 277.9975, found: 277.9963

**(Z)-2-bromo-3-chloro-5-(1-fluoro-2-(4-methoxyphenyl)vinyl)pyridine (11c)**

General procedure A was followed using 2-bromo-3-chloro-5-(trifluoromethyl)pyridine. The product was purified by silica gel column chromatography (hexane/ethyl acetate 30:1 to 10:1). Any excess pinacol was removed by azeotropic distillation with methanol, yielding the compound **11c** as a colorless oil in 90% (31 mg) yield with a 98:2 Z:E.

**^1^H NMR (600 MHz, CDCl_3_)** δ 8.51 (d, *J* = 2.0 Hz, 1H), 7.91 (d, *J* = 2.1 Hz, 1H), 7.60 (d, *J* = 8.8 Hz, 2H), 6.93 (d, *J* = 8.8 Hz, 2H), 6.48 – 6.09 (m, 1H), 3.85 (s, 3H).

**^13^C NMR (151 MHz, CDCl_3_)** δ 159.73 (d, *J* = 3.0 Hz), 151.08 (d, *J* = 255.3 Hz), 142.83 (d, *J* = 7.5 Hz), 140.73, 133.49, 132.78 (d, *J* = 7.2 Hz), 130.85 (d, *J* = 8.1 Hz), 129.87 (d, *J* = 28.8 Hz), 124.98 (d, *J* = 3.2 Hz), 114.29, 109.11 (d, *J* = 9.5 Hz), 55.34.

**^19^F NMR (377 MHz, CDCl_3_)** δ -119.71 (d, *J* = 39.6 Hz).

**HRMS (ESI+) [M+H]+** calcd for C_14_H_11_BrClFNO^+^; 341.9691, found: 341.9704

**(Z)-2-bromo-5-(1-fluoro-2-(thiophen-3-yl)vinyl)pyridine (11d)**

General Procedure A was followed using 3-(trifluoromethyl)benzonitrile (0.10 mmol) and aryl-substituted DBM (1.1 equiv) to avoid secondary *S*_N_Ar addition at the nitrile group. The crude product was purified by column chromatography on silica gel (hexanes/ethyl acetate, gradient from 30:1 to 10:1). Excess pinacol was removed by azeotropic distillation with methanol, affording compound **11d** as a colorless oil in 44% yield (10 mg) with a 98:2 Z:E ratio.

**^1^H NMR (600 MHz, CDCl_3_)** δ 7.91 (d, *J* = 1.5 Hz, 1H), 7.85 (d, *J* = 8.1 Hz, 1H), 7.64 (dt, *J* = 7.7, 1.2 Hz, 1H), 7.60 – 7.57 (m, 1H), 7.54 (t, *J* = 7.9 Hz, 1H), 7.42 (d, *J* = 5.0 Hz, 1H), 7.38 (dd, *J* = 5.0, 2.9 Hz, 1H), 6.52 (d, *J* = 38.7 Hz, 1H).

**^13^C NMR (151 MHz, CDCl_3_)** δ 154.03 (d, *J* = 255.7 Hz), 133.97 (d, *J* = 28.8 Hz), 133.41 (d, *J* = 2.3 Hz), 131.91, 129.55 (d, *J* = 2.1 Hz), 128.24 (d, *J* = 6.5 Hz), 127.88 (d, *J* = 7.0 Hz), 127.47 (d, *J* = 7.7 Hz), 125.85, 125.28 (d, *J* = 8.1 Hz), 118.36, 113.16 (d, *J* = 2.6 Hz), 102.70 (d, *J* = 12.6 Hz).

**^19^F NMR (377 MHz, CDCl_3_)** δ -115.79 (d, *J* = 38.9 Hz).

**HRMS (ESI+) [M+H]** calcd for C_13_H_8_FNS^•+^; 229.0356, found: 229.0356

**(Z)-5-(2-(dibenzo[b,d]furan-4-yl)-1-fluorovinyl)-2-methoxypyridine (11e)**

General procedure A was followed using 2-methoxy-5-(trifluoromethyl)pyridine. The product was purified by silica gel column chromatography (hexane/ethyl acetate 30:1 to 10:1). Any excess pinacol was removed by azeotropic distillation with methanol, yielding the compound **11e** as a colorless oil in 78% yield (25 mg)with a 98:2 Z:E.

**^1^H NMR (400 MHz, CDCl_3_)** δ 8.58 (s, 1H), 8.05 (d, *J* = 7.7 Hz, 1H), 7.95 (t, *J* = 7.8 Hz, 2H), 7.86 (d, *J* = 7.6 Hz, 1H), 7.61 (s, 1H), 7.48 (t, *J* = 7.8 Hz, 1H), 7.37 (q, *J* = 7.3 Hz, 2H), 6.88 (d, *J* = 34.1 Hz, 1H), 6.82 (s, 1H), 4.01 (s, 3H).

**^13^C NMR (101 MHz, CDCl_3_)** δ 164.73, 156.86 (d, *J* = 259.3 Hz), 156.00, 153.64, 143.85 (d, *J* = 8.3 Hz), 135.05 (d, *J* = 6.7 Hz), 127.36, 126.82 (d, *J* = 14.1 Hz), 124.52, 124.11, 123.15 (d, *J* = 25.4 Hz), 122.44 (d, *J* = 27.9 Hz), 120.93, 119.65, 118.47, 111.79, 111.13 (d, *J* = 2.2 Hz), 97.78 (d, *J* = 9.7 Hz), 53.94.

**^19^F NMR (377 MHz, CDCl_3_**) δ -112.99 (d, *J* = 40.1 Hz).

**HRMS (ESI+) [M+H]+** calcd for C_20_H_15_FNO_2_^+^; 320.1081, found: 320.1082

**(Z)-N-(3-(1-fluoro-2-phenylvinyl)-5-(trifluoromethyl)benzyl)-N-(1-(naphthalen-1-yl)ethyl)-3-(3-(trifluoromethyl)phenyl)propan-1-amine (1f)**

General procedure A was followed using cinacalcet derived (1.0 eq), and PMP-diboron (1.5 eq). The product was purified by silica gel column chromatography (hexane/ethyl acetate 30:1 to 10:1). Any excess pinacol was removed by azeotropic distillation with methanol, yielding the compound **11f** as a colorless oil in 20% yield (13 mg)with a 98:2 Z:E.

**^1^H NMR (600 MHz, CDCl_3_)** δ 8.37 (d, *J* = 8.4 Hz, 1H), 7.85 (d, *J* = 8.0 Hz, 1H), 7.75 (d, *J* = 8.1 Hz, 1H), 7.62 (s, 1H), 7.56 (d, *J* = 8.7 Hz, 3H), 7.54 – 7.46 (m, 4H), 7.42 (t, *J* = 7.7 Hz, 2H), 7.37 (d, *J* = 7.6 Hz, 1H), 7.34 (s, 1H), 7.20 (s, 1H), 7.06 (d, *J* = 7.5 Hz, 1H), 6.94 (d, *J* = 8.3 Hz, 2H), 6.07 (d, *J* = 39.6 Hz, 1H), 4.77 (q, *J* = 6.5 Hz, 1H), 3.86 (s, 3H), 3.76 (d, *J* = 14.4 Hz, 1H), 3.60 (d, *J* = 14.4 Hz, 1H), 2.75 (dt, *J* = 13.7, 7.0 Hz, 1H), 2.67 (dt, *J* = 13.6, 7.2 Hz, 1H), 2.54 – 2.32 (m, 3H), 1.73 (tq, *J* = 14.7, 7.0 Hz, 3H), 1.57 (d, *J* = 6.6 Hz, 3H).

**^13^C NMR (151 MHz, CDCl_3_)** δ 159.15, 154.53 (d, *J* = 255.6 Hz), 142.94, 142.75, 139.20, 134.10, 133.63 (d, *J* = 28.3 Hz), 132.04, 131.54, 130.51 (d, *J* = 8.0 Hz), 128.75, 128.62, 128.06, 127.43, 127.00 (d, *J* = 7.1 Hz), 126.20, 125.87, 125.59 (d, *J* = 5.4 Hz), 125.15, 124.99, 124.84, 124.70, 123.31, 122.57, 119.06, 114.10, 106.63 (d, *J* = 10.3 Hz), 56.75, 55.31, 54.90, 51.07, 33.39, 29.19, 13.61.

**^19^F NMR (564 MHz, CDCl_3_)** δ -62.56, -62.75, -117.86. decoupling

**HRMS (ESI+) [M+H]+** calcd for C_39_H_35_F_7_NO^+^: 666.2601, found: 666.2604

**(Z)-N-(3-(1-fluoro-2-phenylvinyl)-5-(trifluoromethyl)benzyl)-N-methyl-3-phenyl-3-(4-(trifluoromethyl)phenoxy)propan-1-amine (11g)**

General procedure A was followed using fluoxetine derived (1.0 eq), and phenyl diboron (1.5 eq). The product was purified by silica gel column chromatography (hexane/ethyl acetate 30:1 to 10:1). Any excess pinacol was removed by azeotropic distillation with methanol, yielding the compound **11g** as a colorless oil in 22% yield(13 mg) with a 98:2 Z:E.

**^1^H NMR (600 MHz, CDCl_3_)** δ 7.73 (s, 1H), 7.67 (s, 1H), 7.65 (d, *J* = 7.5 Hz, 2H), 7.52 (s, 1H), 7.42 – 7.36 (m, 5H), 7.30 (dd, *J* = 6.8, 3.2 Hz, 5H), 6.81 (d, *J* = 8.6 Hz, 2H), 6.35 (d, *J* = 38.7 Hz, 1H), 5.25 (dd, *J* = 8.4, 4.5 Hz, 1H), 3.59 (d, *J* = 13.4 Hz, 1H), 3.53 (d, *J* = 13.4 Hz, 1H), 2.66 (dt, *J* = 14.0, 7.2 Hz, 1H), 2.51 (dt, *J* = 12.5, 6.2 Hz, 1H), 2.27 (s, 3H), 2.18 (td, *J* = 14.5, 7.9 Hz, 3H), 2.01 (ddd, *J* = 14.3, 11.9, 7.2 Hz, 1H).

**^13^C NMR (151 MHz, CDCl_3_)** δ 160.53, 155.69 (d, *J* = 258.0 Hz), 141.14 (d, *J* = 5.0 Hz), 133.70 (d, *J* = 28.6 Hz), 133.04, 131.65, 129.13 (d, *J* = 7.9 Hz), 128.75 (d, *J* = 10.4 Hz), 127.88 (d, *J* = 2.1 Hz), 127.83, 127.61 (d, *J* = 6.8 Hz), 126.74 (d, *J* = 3.7 Hz), 125.88, 125.76, 125.04 (d, *J* = 74.1 Hz), 123.24 (d, *J* = 75.7 Hz), 122.69 (d, *J* = 32.7 Hz), 119.77 (d, *J* = 4.1 Hz), 115.54, 107.35 (d, *J* = 10.1 Hz), 78.09, 61.98, 53.25, 42.51, 36.76..

**^19^F NMR (377 MHz, CDCl_3_)** δ -61.55, -62.64, -114.79 (d, *J* = 39.3 Hz).

**HRMS (ESI+) [M+H]+** calcd for C_33_H_29_F_7_NO^+^; 588.2132, found: 588.2135

- **Synthesis of Alkyne by tris-defluorinative C-C bond coupling**

**Procedure A:** In a nitrogen-filled glovebox, an 8 mL vial equipped with a magnetic stir bar was charged with lithium tetramethylpiperidide (LTMP, 1.5 equiv), Bis [(pinacolato) boryl]methane (DBM, 1.5 equiv), and lithium tert-butoxide (LiOtBu, 1.5 equiv). Anhydrous DME (0.1 M) was added, followed by the trifluoromethyl arene (1.0 equiv, 0.3 mmol), introduced as either a solid or liquid. The vial was sealed being removed from the glovebox. The reaction mixture was allowed to stir at 80 °C for 24 hours. Upon completion, the mixture was allowed to cool to room temperature, quenched with water, and extracted with ethyl acetate. The combined organic layers were dried over Na₂SO₄, filtered, and concentrated under reduced pressure. The crude product was analyzed by ¹H NMR using mesitylene (5 µL) as an internal standard to determine yield. The product was then purified by column chromatography using the appropriate mobile phase.

**Procedure B:** In a nitrogen-filled glovebox, an 8 mL vial equipped with a magnetic stir bar was charged with lithium tetramethylpiperidide (LTMP, 1.5 equiv) and Bis[(pinacolato)boryl]methane (DBM, 1.5 equiv). Anhydrous DMF (0.4 M) was added, followed by the trifluoromethyl arene (1.0 equiv, 0.3 mmol), introduced as either a solid or liquid. The vial was sealed before being removed from the glovebox. The reaction mixture was allowed to stir at 22 °C for 24 hours. After completion, the mixture was quenched with water and extracted with ethyl acetate. The combined organic layers were dried over Na₂SO₄, filtered, and concentrated under reduced pressure. The crude product was analyzed by ¹H NMR using mesitylene (5 µL) as an internal standard to determine the yield. The crude residue was dried under vacuum. In the same reaction vial, lithium tert-butoxide (LiOtBu, 1.5 equiv) was added, followed by anhydrous DME. The mixture was allowed to stir at 80 °C for 24 hours. Upon completion, the reaction was quenched with water and extracted with ethyl acetate. The organic phase was dried over Na₂SO₄, filtered, and concentrated under reduced pressure. The crude product was analyzed by ¹H NMR using mesitylene (5 µL) as an internal standard, and purified by column chromatography using the appropriate mobile phase.

**2-bromo-5-ethynylpyridine (12a)**

General procedure A was followed using 2-bromo-5-trifluoromethyl pyridine. The product was purified by silica gel column chromatography (hexane/ethyl acetate 30:1 to 10:1). Any excess pinacol was removed by azeotropic distillation with methanol, yielding the compound **12a** as a colorless oil with 84% yield (46 mg). The spectra match the literature. ^24^

**^1^H NMR (400 MHz, CDCl_3_)** δ 8.45 (d, *J* = 2.3 Hz, 1H), 7.60 (dd, *J* = 8.2, 2.3 Hz, 1H), 7.49 – 7.40 (m, 1H), 3.27 (s, 1H).

**5-ethynyl-2-methoxypyridine (12b)**

General procedure A was followed using 2-methoxy-5-trifluoromethyl pyridine. The product was purified by silica gel column chromatography (hexane/ethyl acetate 30:1 to 10:1). Any excess pinacol was removed by azeotropic distillation with methanol, yielding the compound **12b** as a colorless oil 50% yield (20 mg). The spectra match the literature.^25^

**^1^H NMR (400 MHz, CDCl_3_)** δ 8.31 (dd, *J* = 2.3, 0.8 Hz, 1H), 7.64 (dd, *J* = 8.6, 2.3 Hz, 1H), 6.70 (dd, *J* = 8.6, 0.8 Hz, 1H), 3.94 (s, 3H), 3.13 (s, 1H).

**5-ethynyl-2-phenylpyridine (12c)**

General procedure A was followed using 2-phenyl-5-trifluoromethyl pyridine. The product was purified by silica gel column chromatography (hexane/ethyl acetate 30:1 to 10:1). Any excess pinacol was removed by azeotropic distillation with methanol, yielding the compound **12c** as a colorless oil 62% yield (33 mg). The spectra match the literature^26^

**^1^H NMR (400 MHz, CDCl_3_)** δ 8.80 (d, *J* = 2.1 Hz, 1H), 8.04 – 7.96 (m, 2H), 7.84 (dd, *J* = 8.2, 2.2 Hz, 1H), 7.71 (dd, *J* = 8.2, 0.9 Hz, 1H), 7.53 – 7.39 (m, 3H), 3.26 (s, 1H).

**5-ethynyl-2-(phenylethynyl)pyridine (12d)**

General procedure A was followed using 2-(phenylethynyl)-5-(trifluoromethyl)pyridine. The product was purified by silica gel column chromatography (hexane/ethyl acetate 30:1 to 10:1). Any excess pinacol was removed by azeotropic distillation with methanol, yielding the compound **12d** as a colorless oil 68% yield (41 mg).

**^1^H NMR (600 MHz, CDCl_3_)** δ 8.71 (dd, *J* = 2.1, 0.9 Hz, 1H), 7.76 (dd, *J* = 8.1, 2.1 Hz, 1H), 7.63 – 7.56 (m, 2H), 7.48 (dd, *J* = 8.1, 0.9 Hz, 1H), 7.42 – 7.34 (m, 3H), 3.31 (s, 1H).

**^13^C NMR (151 MHz, CDCl_3_)** δ 153.02, 142.72, 139.16, 132.13, 129.28, 128.47, 126.36, 121.97, 118.19, 91.37, 88.33, 82.15, 80.26.

**HRMS (ESI+) [M+H]+** calcd for C_15_H_10_N^+^; 204.0808, found: 204.0813

**3-ethynyl-5-(3-(methylthio)phenyl)pyridine (12e)**

General procedure A was followed using 3-(3-(methylthio)phenyl)-5-(trifluoromethyl)pyridine. The product was purified by silica gel column chromatography (hexane/ethyl acetate 30:1 to 10:1). Any excess pinacol was removed by azeotropic distillation with methanol, yielding the compound **12e** as a colorless oil 72% yield (49 mg).

**^1^H NMR (600 MHz, CDCl_3_)** δ 8.77 (d, *J* = 2.3 Hz, 1H), 8.70 (d, *J* = 2.0 Hz, 1H), 7.94 (d, *J* = 2.2 Hz, 1H), 7.43 – 7.36 (m, 2H), 7.29 (ddd, *J* = 9.6, 5.5, 1.9 Hz, 2H), 3.28 (d, *J* = 1.1 Hz, 1H), 2.53 (s, 3H).

**^13^C NMR (151 MHz, CDCl_3_)** δ 151.45, 147.66, 139.88, 137.39, 135.75, 129.55, 126.31, 125.01, 123.81, 119.23, 81.02, 80.30, 24.74, 15.73.

**HRMS (ESI+) [M+H]+** calcd for C_14_H_12_NS^+^; 226.0685, found: 226.0691

**tert-butyl 4-(5-ethynylpyridin-3-yl)piperazine-1-carboxylate (12f)**

General procedure A was followed using tert-butyl 4-(5-(trifluoromethyl)pyridin-3-yl)piperazine-1-carboxylate. The product was purified by silica gel column chromatography (hexane/ethyl acetate 30:1 to 10:1). Any excess pinacol was removed by azeotropic distillation with methanol, yielding the compound **12f** as a colorless oil 84% yield (72 mg). The spectrum matches the literature. ^27^

**^1^H NMR (600 MHz, CDCl_3_)** δ 8.23 (s, 1H), 8.19 (s, 1H), 7.22 (s, 1H), 3.56 (t, *J* = 5.2 Hz, 4H), 3.15 (m, 5H), 1.46 (s, 9H).

**2-bromo-3-chloro-5-ethynylpyridine (12g)**

General procedure A was followed using 2-bromo-3-chloro-5-trifluoromethyl pyridine. The product was purified by silica gel column chromatography (hexane/ethyl acetate 30:1 to 10:1). Any excess pinacol was removed by azeotropic distillation with methanol, yielding the compound **12g** as a colorless oil in 75% yield (50 mg).

**^1^H NMR (600 MHz, CDCl_3_)** δ 8.34 (d, *J* = 2.0 Hz, 1H), 7.79 (d, *J* = 2.0 Hz, 1H), 3.31 (s, 1H).

**^13^C NMR (151 MHz, CDCl_3_)** δ 150.12, 141.56, 140.54, 133.06, 119.54, 83.00, 77.93.

**HRMS (ESI+) [M+H]+** calcd for C_7_H_4_BrClN^+^; 215.9210, found: 215.9213

**3-ethynylbenzonitrile (12h)**

General procedure B was followed using 3-(trifluoromethyl)benzonitrile. The product was purified by silica gel column chromatography (hexane/ethyl acetate 20:1 to 5:1). Any excess pinacol was removed by azeotropic distillation with methanol, yielding the compound **12h** as a colorless oil 91% yield (35 mg). The spectrum matches the literature.^28^

**^1^H NMR (600 MHz, CDCl_3_)** δ 7.73 (s, 1H), 7.67 (d, *J* = 7.8 Hz, 1H), 7.60 (d, *J* = 7.9 Hz, 1H), 7.43 (t, *J* = 7.8 Hz, 1H), 3.20 (s, 1H).

**2-(3-ethynyl-5-(trifluoromethyl)phenyl)pyridine (12l)**

General procedure B was followed using 1,3-bis(trifluoromethyl)benzene. The product was purified by silica gel column chromatography (hexane/ethyl acetate 30:1 to 10:1). Any excess pinacol was removed by azeotropic distillation with methanol, yielding the compound **12l** as a colorless oil 68% yield (50 mg).

**^1^H NMR (600 MHz, CDCl_3_)** δ 8.75 (d, *J* = 5.0 Hz, 1H), 8.31 (d, *J* = 9.0 Hz, 2H), 7.84 – 7.77 (m, 3H), 7.33 (ddd, *J* = 7.4, 4.7, 1.2 Hz, 1H), 3.21 (s, 1H).

**^13^C NMR (151 MHz, CDCl_3_)** δ 154.83, 150.01, 140.48, 137.11, 133.49, 131.60 (q, *J* = 32.8 Hz), 129.02 (d, *J* = 3.8 Hz), 124.53, 124.00 (q, *J* = 3.7 Hz), 123.61, 123.22, 120.60, 82.03, 78.98.

**^19^F NMR (377 MHz, CDCl_3_) δ -62.87.**

**HRMS (ESI+) [M+H]+** calcd for C_14_H_9_F_3_N^+^; 248.0682, found: 248.0681

- **Synthesis of internal alkynes**

**Procedure A:** In a nitrogen-filled glovebox, an 8 mL vial equipped with a magnetic stir bar was charged with lithium tetramethylpiperidide (LTMP, 1.5 equiv), Bis[(pinacolato)boryl]methane (DBM, 1.5 equiv), and lithium tert-butoxide (LiOtBu, 1.5 equiv). Anhydrous DME (0.1 M) was added, followed by the trifluoromethyl arene (1.0 equiv, 0.300 mmol), introduced as a solid or liquid. The vial was sealed before being removed from the glovebox. The reaction mixture was allowed to stir at 80 °C for 24 hours. Upon completion, the reaction was allowed to cool to room temperature, quenched with water, and extracted with ethyl acetate. The combined organic layers were dried over Na₂SO₄, filtered, and concentrated under reduced pressure. The crude product was analyzed by ¹H NMR using as an internal standard to determine yield. The product was purified by column chromatography using the appropriate mobile phase.

**tert-butyl 4-(5-(prop-1-yn-1-yl)pyridin-3-yl)piperazine-1-carboxylate (12j)**

General procedure A was followed using tert-butyl 4-(5-(trifluoromethyl)pyridin-3-yl)piperazine-1-carboxylate. The product was purified by silica gel column chromatography (hexane/ethyl acetate 30:1 to 10:1). Any excess pinacol was removed by azeotropic distillation with methanol, yielding the compound **12j** as a colorless oil 94% yield (85 mg).

**^1^H NMR (600 MHz, CDCl_3_)** δ 8.18 (s, 1H), 8.12 (s, 1H), 7.17 (s, 1H), 3.57 (s, 4H), 3.15 (t, *J* = 5.2 Hz, 4H), 2.06 (t, *J* = 5.2 Hz, 3H), 1.48 (s, 9H).

**^13^C NMR (151 MHz, CDCl_3_)** δ 154.60, 146.19, 143.41, 137.50, 125.11, 120.85, 88.98, 80.17, 77.31, 76.79, 48.42, 28.40, 4.43.

**HRMS (ESI+) [M+H]+** calcd for C_17_H_24_N_3_O_2_^+^; 302.1863, found: 302.1870

**tert-butyl4-(5-(4-((tert-butyldimethylsilyl)oxy)but-1-yn-1-yl)pyridin-3-yl)piperazine-1-carboxylate (12k)**

General procedure A was followed using tert-butyl 4-(5-(trifluoromethyl)pyridin-3-yl)piperazine-1-carboxylate. The product was purified by silica gel column chromatography (hexane/ethyl acetate 30:1 to 10:1). Any excess pinacol was removed by azeotropic distillation with methanol, yielding the compound **12k** as a colorless oil 91% yield (122 mg).

**^1^H NMR (600 MHz, CDCl_3_)** δ 8.18 (s, 1H), 8.12 (s, 1H), 7.16 (s, 1H), 3.81 (t, *J* = 7.0 Hz, 2H), 3.57 (t, *J* = 5.2 Hz, 4H), 3.14 (t, *J* = 5.1 Hz, 4H), 2.62 (t, *J* = 6.9 Hz, 2H), 1.47 (s, 9H), 0.91 (s, 9H), 0.09 (s, 6H).

**^13^C NMR (151 MHz, CDCl_3_)** δ 154.61, 146.16, 143.53, 137.71, 125.12, 120.60, 90.36, 80.15, 78.64, 61.71, 48.45, 28.41, 25.90, 24.87, 23.85, 5.23.

**HRMS (ESI+) [M+H]+** calcd for C_24_H_40_N_3_O_3_Si^+^; 446.2833, found: 446.2844

**tert-butyl 4-(5-(pent-1-yn-1-yl)pyridin-3-yl)piperazine-1-carboxylate (12l)**

General procedure A was followed using tert-butyl 4-(5-(trifluoromethyl)pyridin-3-yl)piperazine-1-carboxylate. The product was purified by silica gel column chromatography (hexane/ethyl acetate 30:1 to 10:1). Any excess pinacol was removed by azeotropic distillation with methanol, yielding the compound **12l** as a colorless oil 90% yield (89 mg).

**^1^H NMR (600 MHz, CDCl_3_)** δ 8.18 (s, 1H), 8.13 (s, 1H), 7.21 – 7.16 (s, 1H), 3.58 (t, *J* = 5.2 Hz, 4H), 3.16 (t, *J* = 5.2 Hz, 4H), 2.40 (t, *J* = 7.1 Hz, 3H), 1.64 (q, *J* = 7.2 Hz, 2H), 1.48 (s, 9H), 1.05 (t, *J* = 7.4 Hz, 2H).

**^13^C NMR (151 MHz, CDCl_3_)** δ 154.61, 146.19, 143.51, 137.47, 125.21, 120.91, 93.37, 80.17, 74.97, 48.47, 28.41, 24.86, 22.05, 21.41, 13.61.

**HRMS (ESI+) [M+H]+** calcd for C_19_H_28_N_3_O_2_^+^; 330.2176, found: 330.2184

- **Synthetic utility of alkenyl fluoride compounds**
  - **Defluoroalkenylation/ oxidation reaction**

**(E)-3-(1-fluoro-2-oxopent-3-en-1-yl)benzonitrile (13):** In a nitrogen-filled glovebox, an 8 mL vial equipped with a magnetic stir bar was charged with cesium fluoride (CsF, 3.0 equiv), 18-crown-6 (40 mol %), and TMS-DBM (3.0 equiv). Anhydrous DMF (0.50 mL, 0.2M) was added, followed by the trifluoromethyl arene (1.0 equiv, 0.100 mmol), introduced as either a solid or liquid.

The vial was sealed before being removed from the glovebox. The reaction mixture was allowed to stir at 22 °C for 24 hours. Upon completion, the reaction was quenched with water and extracted with saturated aqueous LiCl (3×) and ethyl acetate to remove DMF. The mixture was allowed to cool to room temperature, filtered through a Celite plug, and rinsed with ethyl acetate. The solvent was removed under reduced pressure.

The resulting residue was dissolved in a 1:1 mixture of THF and water and cooled to 0 °C, followed by the addition of sodium perborate (NaBO₃, 2.0 equiv). The reaction mixture was allowed to stir at room temperature for 3 hours. The mixture was then quenched at 0 °C with saturated sodium thiosulfate (Na₂S₂O₃) and extracted with ethyl acetate. The organic layers were combined, dried over Na₂SO₄, filtered, and concentrated under reduced pressure. The product was then purified by column chromatography to afford compound **13** in 82% yield (25.8 mg).

**^1^H NMR (600 MHz, CDCl_3_)** δ 7.74 (s, 1H), 7.67 (d, *J* = 8.2 Hz, 2H), 7.52 (t, *J* = 7.8 Hz, 1H), 7.20 – 7.09 (m, 1H), 5.82 (d, *J* = 48.4 Hz, 1H), 1.96 (dd, *J* = 7.0, 1.7 Hz, 3H).

**^13^C NMR (151 MHz, CDCl_3_)** δ 193.44 (d, *J* = 23.4 Hz), 148.16, 136.05 (d, *J* = 20.9 Hz), 132.68, 130.31 (d, *J* = 8.2 Hz), 129.67, 129.48 (d, *J* = 9.4 Hz), 124.45, 118.15, 113.14, 93.97 (d, *J* = 189.8 Hz), 18.85.

**^19^F NMR (377 MHz, CDCl_3_)** δ -187.92 (d, *J* = 48.3 Hz).

**HRMS (ESI+) [M+Na]+** calcd for C_12_H_10_FNNaO_+_; 226.0639, found: 226.0642

**3-(3-(1,3-dioxolan-2-yl)-1-fluoro-2-oxopropyl)benzonitrile (14) :** In a nitrogen-filled glovebox, an 8 mL vial equipped with a magnetic stir bar was charged with cesium fluoride (CsF, 3.0 equiv), 18-crown-6 (40 mol %), and substituted TMS-DBM (3.0 equiv). Anhydrous DMF (0.5 mL, 0.2M) was added, followed by the trifluoromethyl arene (1.0 equiv, 0.100 mmol), introduced as either a solid or liquid.

The vial was sealed with a hard cap before being removed from the glovebox. The reaction mixture was allowed to stir at 22 °C for 24 hours. Upon completion, the reaction was quenched with water and extracted with saturated aqueous LiCl and ethyl acetate to remove DMF. The mixture was allowed to cool to room temperature, filtered through a Celite plug, and rinsed with ethyl acetate. The solvent was removed under reduced pressure.

The resulting residue was dissolved in a 1:1 mixture of THF and water and cooled to 0 °C, followed by the addition of sodium perborate (NaBO₃, 2.0 equiv). The reaction mixture was allowed to stir at room temperature for 3 hours. It was then quenched at 0 °C with saturated sodium thiosulfate (Na₂S₂O₃) and extracted with ethyl acetate. The organic layers were combined, dried over Na₂SO₄, filtered, and concentrated under reduced pressure. The product was then purified by column chromatography to afford compound **14** in 64% yield (16 mg).

**^1^H NMR (600 MHz, CDCl_3_)** δ 7.74 (s, 1H), 7.67 (dd, *J* = 13.3, 7.9 Hz, 2H), 7.53 (t, *J* = 7.8 Hz, 1H), 5.76 (d, *J* = 47.6 Hz, 1H), 5.27 (t, *J* = 4.9 Hz, 1H), 4.04 – 3.92 (m, 2H), 3.90 – 3.81 (m, 2H), 3.05 (dt, *J* = 16.5, 4.1 Hz, 1H), 2.95 (ddd, *J* = 16.5, 5.1, 2.4 Hz, 1H).

**^13^C NMR (151 MHz, CDCl_3_)** δ 202.41 (d, *J* = 26.8 Hz), 135.26 (d, *J* = 21.1 Hz), 132.96, 130.41 (d, *J* = 7.1 Hz), 129.82, 129.68 (d, *J* = 8.2 Hz), 118.22, 113.32, 100.26 (d, *J* = 2.2 Hz), 94.49 (d, *J* = 190.1 Hz), 65.16 (d, *J* = 7.4 Hz), 42.91.

**^19^F NMR (565 MHz, CDCl_3_)** δ -187.94 (d, *J* = 47.8 Hz).

**HRMS (ESI+) [M+Na]+** calcd for C_13_H_12_FNNaO_3_^+^; 272.0693, found: 272.0698

- **Cross-coupling reaction**

**(Z)-5-(1-fluoro-2-phenylvinyl)-2-phenylpyridine (15):** In a nitrogen-filled glovebox, an 8 ml vial equipped with a stir bar was charged with 2-bromo pyridine (2.0 eq), alkenyl fluoride (0.11 mmol) (1.0 eq), Pd(PPh_3_)_4_ (5 mol %), and K_2_CO_3_ (3 eq) were added into the reaction vial. The reaction vial was sealed and brought out of the glove box. Then a dioxane/water (3:1) (0.1M) was added. The reaction was allowed to stir for 2.5 h at 80 °C. The reaction was then allowed to cool to room temperature, and sat. LiCl was added and extracted with ethyl acetate three times. The reaction was then concentrated under reduced pressure. The crude product was purified by column chromatography to give **15** in 84% yield (25 mg) as a colorless oil.

**^1^H NMR (600 MHz, CDCl_3_)** δ 8.99 (s, 1H), 8.05 (d, *J* = 7.6 Hz, 2H), 7.97 (d, *J* = 10.4 Hz, 1H), 7.79 (d, *J* = 8.4 Hz, 1H), 7.68 (d, *J* = 7.7 Hz, 2H), 7.50 (t, *J* = 7.5 Hz, 2H), 7.45 (t, *J* = 7.4 Hz, 1H), 7.41 (t, *J* = 7.7 Hz, 2H), 7.31 (t, *J* = 7.4 Hz, 1H), 6.40 (d, *J* = 39.6 Hz, 1H).

**^13^C NMR (151 MHz, CDCl_3_)** δ 157.35, 154.97 (d, *J* = 258.1 Hz), 145.73 (d, *J* = 7.6 Hz), 138.59, 133.12 (d, *J* = 3.1 Hz), 132.24 (d, *J* = 6.7 Hz), 129.42, 129.15 (d, *J* = 8.0 Hz), 128.89, 128.72, 127.86 (d, *J* = 2.5 Hz), 127.20 (d, *J* = 28.2 Hz), 126.93, 119.97 (d, *J* = 2.1 Hz), 107.28 (d, *J* = 9.4 Hz).

**^19^F NMR (377 MHz, CDCl_3_)** δ -116.10 (d, *J* = 39.5 Hz).

**HRMS (ESI+) [M+H]+** calcd for C_19_H_15_FN^+^; 276.1183, found: 276.1183

- **Cross-coupling reaction of trisubstituted alkenyl fluorides**

**(Z)5(2-fluoro-2-(6-methoxypyridin-3-yl)vinyl)-1-methyl-1H-indole (16):** In a nitrogen-filled glovebox, an 8 ml vial equipped with a stir bar was charged with 5-bromo-1- methylindole (1.5 eq), alkenyl fluoride (0.13 mmol) (1.0 eq), Pd(PPh_3_)_4_ (5 mol %), and K_2_CO_3_ (3 eq) were added into the reaction vial. The reaction vial was sealed and brought out of the glove box. Then, a dioxane/water (3:1) (0.1M) was added. The reaction was allowed to stir for 2.5 h at 80°C. Then, the reaction was allowed to cool down to room temperature, and sat. LiCl was added and extracted with ethyl acetate three times. The reaction was then concentrated under reduced pressure, and the crude product was purified by column chromatography to give **16** in 79% yield (28 mg) as a colorless oil.

**^1^H NMR (600 MHz, CDCl_3_**) δ 8.47 (d, *J* = 2.5 Hz, 1H), 7.90 (s, 1H), 7.81 (dd, *J* = 8.7, 2.5 Hz, 1H), 7.53 (dd, *J* = 8.6, 1.6 Hz, 1H), 7.32 (d, *J* = 8.6 Hz, 1H), 7.06 (d, *J* = 3.1 Hz, 1H), 6.78 (d, *J* = 8.6 Hz, 1H), 6.50 (d, *J* = 3.1 Hz, 1H), 6.29 (d, *J* = 40.4 Hz, 1H), 3.98 (s, 3H), 3.81 (s, 3H).

**^13^C NMR (151 MHz, CDCl_3_)** δ 164.03, 153.48 (d, *J* = 253.8 Hz), 142.94 (d, *J* = 8.1 Hz), 136.04 (d, *J* = 2.3 Hz), 134.46 (d, *J* = 6.5 Hz), 129.47, 128.73, 124.81 (d, *J* = 3.3 Hz), 123.22, 122.96 (d, *J* = 7.6 Hz), 121.61 (d, *J* = 8.0 Hz), 110.78 (d, *J* = 2.0 Hz), 109.31, 106.52 (d, *J* = 9.9 Hz), 101.44, 53.69, 32.93.

**^19^F NMR (377 MHz, CDCl_3_)** δ -118.60 (d, *J* = 40.5 Hz).

**HRMS (ESI+) [M+H]+** calcd for C_17_H_16_FN_2_O^+^; 283.1241, found: 283.1245

- **Cross-coupling reaction of tetrasubstituted alkenyl fluoride**

**(Z)-5-(2-(3,5-difluorophenyl)-1-fluorovinyl)-2-methoxypyridine (17):** In a nitrogen-filled glovebox, an 8 mL vial equipped with a magnetic stir bar was charged with cesium fluoride (CsF, 3.0 equiv), 18-crown-6 (40 mol %), and TMS-DBM (3.0 equiv). Anhydrous DMF (0.2M) was added, followed by trifluoromethyl arene (1.0 equiv, 0.100 mmol) as a liquid.

The vial was sealed with a hard cap and secured with electrical tape before being removed from the glovebox. The reaction mixture was allowed to stir at 22 °C for 24 hours. Upon completion, the reaction was quenched with water and extracted with saturated aqueous LiCl and ethyl acetate to remove DMF. The combined organic layers were dried over Na₂SO₄ and concentrated under reduced pressure. The crude residue was azeotropically dried with benzene and brought back into the glovebox.

In the glovebox, the crude intermediate was charged with Pd(PPh₃)₄ (5 mol %) and potassium carbonate (K₂CO₃, 3.0 equiv). The vial was sealed with a PTFE/silicone-lined septum cap, secured with electrical tape, and removed from the glovebox. 1-Bromo-3,5-difluorobenzene (2.0 equiv) was then added, followed by a 3:1 ethanol/water mixture (0.1 M final concentration). The reaction mixture was stirred at 80 °C for 12 hours. After cooling to room temperature, water was added, and the mixture was extracted with ethyl acetate. The combined organic layers were dried over Na₂SO₄, filtered, and concentrated under reduced pressure. The crude product was purified by column chromatography to afford compound 17 in 40% yield (11.3 mg) over two steps with a 98:2 Z:E.

**^1^H NMR (600 MHz, CDCl_3_)** δ 8.34 (s, 1H), 7.71 (dd, *J* = 8.6, 2.4 Hz, 1H), 7.03 (d, *J* = 7.2 Hz, 2H), 6.82 (d, *J* = 8.6 Hz, 1H), 6.74 (tt, *J* = 8.8, 2.3 Hz, 1H), 3.99 (s, 3H), 2.09 (d, *J* = 3.2 Hz, 3H).

**^13^C NMR (151 MHz, CDCl_3_**) δ 164.30, 162.76 (d, *J* = 246.9 Hz), 152.76, 147.47, 138.50, 121.93 (d, *J* = 29.4 Hz), 111.35 (t, *J* = 5.3 Hz), 111.21 (t, *J* = 4.2 Hz), 110.77, 102.60 (t, *J* = 25.8 Hz), 53.75, 18.07.

**^19^F NMR (564 MHz, CDCl_3_)** δ -99.66, -110.50.

**HRMS (ESI+) [M+H]+** calcd for C_15_H_13_F_3_NO^+^; 280.0944, found: 280.0946

- **Synthesis of indole by alkyne Larock indole synthesis**

**2-(6-phenylpyridin-3-yl)-1H-indole (19):** In an 8 mL vial equipped with a magnetic stir bar, Pd(OAc)_2_ (0.0125 mmol), LiCl (0.5 mmol) , the K_2_CO_3_ (5 eq), the aryl iodide (1.1 eq), the alkyne (0.17 mmol, 1.0 eq), DMF (0.1M), were added. The reaction was allowed to stir for 24 h at 100 °C. After completion, the reaction mixture was diluted with ether and washed with saturated aqueous NH_4_Cl and H_2_O. The organic layers were dried over MgSO_4_. The reaction mixture was then filtered and concentrated, and the product was purified by flash column chromatography using hexanes-ethyl acetate to afford the product as a white solid in 72% yield (33 mg).^29^

**^1^H NMR (600 MHz, Acetone)** δ 9.20 (d, *J* = 2.6 Hz, 1H), 8.29 (dd, *J* = 8.3, 2.4 Hz, 1H), 8.19 (d, *J* = 7.1 Hz, 2H), 8.04 – 8.02 (m, 1H), 7.62 (d, *J* = 7.9 Hz, 1H), 7.51 (t, *J* = 7.5 Hz, 2H), 7.47 – 7.42 (m, 2H), 7.15 (ddd, *J* = 8.1, 6.9, 1.2 Hz, 1H), 7.08 – 7.06 (m, 2H).

**^13^C NMR (151 MHz, Acetone)** δ 155.20, 146.29, 138.82, 132.83, 128.95, 128.67, 126.49, 122.35, 120.45, 119.96, 119.89, 111.27, 111.22, 100.25, 100.20.

**HRMS (ESI+) [M+H]+** calcd for C_19_H_15_N_2_^+^; 271.1230, found: 271.1229

- **Synthesis of Benzofurans**

**tert-butyl 4-(5-(benzofuran-2-yl)pyridin-3-yl)piperazine-1-carboxylate (20):** In an 8 mL vial equipped with a magnetic stir bar, 2-iodophenol (1.1 eq), alkyne (0.17 mmol, 1.0 eq), KOtBu (3 equiv.), trans-1,2- diaminocyclohexane (20 mol %), CuI (10 mol %)  and 1,4- dioxane (0.15 M) were added. The reaction mixture was allowed to stir at 110 °C for 24 hours. After the completion of the reaction (monitored by TLC), the reaction mixture was allowed to cool to room temperature. The cooled mixture was partitioned between ethyl acetate and water. The organic layer was separated, and the aqueous layer was extracted with ethyl acetate twice. The combined organic layers were washed with K_2_CO_3_ solution, dried over Na_2_SO_4_, and concentrated in vacuo. The residual oil was loaded on a silica gel column and eluted with petroleum ether/ethyl acetate to afford the desired product 20 in 90% yield (60 mg).^30^

**^1^H NMR (600 MHz, CDCl_3_)** δ 8.62 (s, 1H), 8.29 (s, 1H), 7.65 (s, 1H), 7.63 (d, *J* = 8.4 Hz, 1H), 7.56 (d, *J* = 7.7 Hz, 1H), 7.38 (s, 1H), 7.34 (t, *J* = 7.1 Hz, 1H), 7.28 (t, *J* = 7.5 Hz, 1H), 7.13 (s, 1H), 3.66 (t, *J* = 5.1 Hz, 4H), 3.29 (t, *J* = 5.1 Hz, 4H), 1.52 (s, 9H).

**^13^C NMR (151 MHz, CDCl_3_)** δ 155.01, 154.63, 153.18, 146.88, 138.44, 137.86, 128.83, 124.87, 123.25, 121.21, 118.38, 111.26, 102.79, 80.18, 48.56, 28.44.

**HRMS (ESI+) [M+H]+** calcd for C_22_H_26_N_3_O_3_^+^; 380.1969, found: 380.1949

- **Click reaction**

**tert-butyl 4-(5-(1-benzyl-1H-1,2,3-triazol-4-yl)pyridin-3-yl)piperazine-1-carboxylate (18) :** In an 8 mL vial equipped with a magnetic stir bar, terminal alkyne (0.14 mmol, 1.0 mmol), benzyl azide (1.1 eq), CuSO_4_·5H_2_O (4 mol %), and sodium ascorbate (11 mol %) were suspended in a mixture of H_2_O–CH_2_Cl_2_ (1:3, 0.25 M). The reaction mixture was allowed to stir at r.t. for 24 h. After completion, the mixture was extracted CH_2_Cl_2_. The combined organic layers were dried over MgSO_4_, filtered, and concentrated under vacuum. Purification of the crude product using flash column chromatography (hexanes–EtOAc) afforded the product **18** as a white solid in 95% yield (55 mg).^31^

**^1^H NMR (600 MHz, CDCl_3_)** δ 8.36 (s, 1H), 8.24 (s, 1H), 7.77 (s, 1H), 7.73 (s, 1H), 7.42 – 7.36 (m, 3H), 7.33 – 7.28 (m, 2H), 5.58 (s, 2H), 3.59 (t, *J* = 5.2 Hz, 4H), 3.23 (t, *J* = 5.1 Hz, 4H), 1.48 (s, 9H).

**^13^C NMR (151 MHz, CDCl_3_)** δ 154.64, 147.02, 145.32, 138.28, 138.07, 134.41, 129.25, 128.95, 128.13, 126.70, 119.91, 119.47, 80.12, 65.86, 54.39, 48.46, 28.42.

**HRMS (ESI+) [M+H]+** calcd for C_23_H_29_N_6_O_2_^+^; 421.2347, found: 421.2357

- **Mechanistic experiments**
- Siliconate vs boronate reaction

**2,2'-(2-phenylethane-1,1-diyl)bis(4,4,5,5-tetramethyl-1,3,2-dioxaborolane) (21):** In a nitrogen-filled glovebox, an 8 mL vial equipped with a magnetic stir bar was charged with cesium fluoride (CsF, 3.0 equiv), 18-crown-6 (40 mol% %), and TMS-DBM (3.0 equiv). Anhydrous DMF (0.5 mL, 0.2M) was added, followed by the benzyl bromide (1.0 equiv, 0.100 mmol). The reaction mixture was allowed to stir for 18 hours at 22 °C. Then, the reaction mixture was quenched with water, extracted with ethyl acetate, and dried. The solvent was removed under reduced pressure, and the crude product was purified by column chromatography to give the product **21** in 66% yield (23.6 mg). All the spectra matched with the literature data.^32^ This data confirms that the reaction proceeds through the silicate activation.

**^1^H NMR (400 MHz, CDCl_3_)** δ 7.25 – 7.18 (m, 4H), 7.14 – 7.08 (m, 1H), 2.88 (d, J = 8.4 Hz, 2H), 1.19 – 1.16 (m, 25H).

- In Situ ¹⁹F NMR Observation of Fluoride and Silicate Formation:

In a nitrogen-filled glovebox, an 8 mL vial equipped with a magnetic stir bar was charged with cesium fluoride (CsF, 1.0 equiv), 18-crown-6 (10 mol %), and a substituted TMS-DBM (1.0 equiv). Anhydrous MeCN-D_3_ was added to achieve a final concentration of 0.1 M. The reaction mixture was stirred at 22 °C for 30 minutes.

After stirring, the reaction mixture was transferred to an NMR tube, sealed, and removed from the glovebox. ¹⁹F NMR analysis was performed immediately. Three distinct peaks were observed: one at–122.4 ppm, which are attributed to the formation of a possible siliconate intermediate **(26)**. A peak at –127.3 ppm appeared as a quartet, which we assign to a borate intermediate (**27**). Additionally, a singlet at –157.6 ppm was observed, corresponding to (CH₃)₃Si–F (TMS-F) **(28)**.

After 4 hours, a second ¹⁹F NMR analysis under identical conditions showed the disappearance of all peaks except that of TMS-F, indicating full consumption of the intermediates.

As shown in Figure S6, the observed splitting pattern for TMS-F **(28)** is consistent with literature values.²⁸

**^19^F NMR (565 MHz, CD_3_CN)** δ -122.38, -127.26 (q, *J* = 37.3 Hz), -157.59.

Figure S5: ^19^F NMR: Substituted Silyl DB + CsF + 18-crown-6 after 30 min in MeCN-D_3_

Figure S6: ^19^F NMR: Substituted Silyl DB + CsF + 18-crown-6 after 4 hours in MeCN-D_3_

- **Tempo additive:**

In a nitrogen-filled glovebox, an 8 mL vial equipped with a magnetic stir bar was charged with cesium fluoride (CsF, 3.0 equiv), 18-crown-6 (40 mol % ), Tempo (3 eq), and TMS-DBM (3.0 equiv). DMF (0.4 M) was then added, followed by the addition of the trifluoromethyl arene (1.0 equiv, 0.100 mmol; actual amount varied as noted for specific substrates), either as a solid or liquid. The vial was sealed with a hard cap and secured with electrical tape before being removed from the glovebox. The reaction mixture was allowed to stir at 22 °C for 24 h. Upon completion, the reaction was quenched with water and extracted with ethyl acetate. The organic layers were then washed with saturated aqueous LiCl to remove DMF. The combined organic layers were dried over Na₂SO₄, filtered, and concentrated under reduced pressure. The crude product was analyzed by NMR using mesitylene (5 µL) as an internal standard to determine yield. The NMR yield for the reaction using TEMPO was 10% of **8a.**

- **Gram Scale Reaction**

**(Z)-5-(1-fluoro-2-(4,4,5,5-tetramethyl-1,3,2-dioxaborolan-2-yl)vinyl)-2-phenylpyridine (8k):** Following procedure A: In a nitrogen-filled glovebox, a round-bottom flask equipped with a magnetic stir bar was charged with cesium fluoride (2.0 g, 3.0 equiv), 18-crown-6 (0.24 g, 40 mol % ), and TMS-DBM (4.6 g, 3.0 equiv). DMF (11 mL, 0.4M) was then added, followed by the addition of the trifluoromethyl arene (1.0 g, 1.0 equiv). The flask was sealed and secured with electrical tape before being removed from the glovebox. The reaction mixture was allowed to stir at 22 °C for 24 h. Upon completion, the reaction was quenched with water and extracted with ethyl acetate. The organic layers were then washed with saturated aqueous LiCl to remove DMF. The combined organic layers were dried over Na₂SO₄, filtered, and concentrated under reduced pressure. The product was purified by silica gel column chromatography (hexane/ethyl acetate 30:1 to 3:1), yielding the compound **8k** as a colorless oil (1.2 g, 79% yield) with a 98:2 Z:E.

**References**

(1)	Joannou, M. V.; Moyer, B. S.; Goldfogel, M. J.; Meek, S. J. Silver(I)‐Catalyzed Diastereoselective Synthesis of *Anti* ‐1,2‐Hydroxyboronates. *Angew Chem Int Ed* **2015**, *54* (47), 14141–14145. https://doi.org/10.1002/anie.201507171.

(2)	Hazra, S.; Panda, S. Stereoselective Synthesis of Silylated Vinylboronates by a Boron‐Wittig Reaction and Their Application to Tetrasubstituted Olefins. *Chemistry A European J* **2024**, *30* (9), e202303056. https://doi.org/10.1002/chem.202303056.

(3)	Huang, M.; Sun, H.; Seufert, F.; Friedrich, A.; Marder, T. B.; Hu, J. Photoredox/Cu‐Catalyzed Decarboxylative C(Sp^3^ )−C(Sp^3^ ) Coupling to Access C(Sp^3^ )‐Rich *Gem* ‐Diborylalkanes. *Angew Chem Int Ed* **2024**, *63* (33), e202401782. https://doi.org/10.1002/anie.202401782.

(4)	Liu, C.; Lv, X.; Xing, Y.; Qiu, J. Trifluoromethyl-Substituted Cyclometalated Iridium^III^ Emitters with High Photostability for Continuous Oxygen Sensing. *J. Mater. Chem. C* **2015**, *3* (31), 8010–8017. https://doi.org/10.1039/C5TC01599E.

(5)	Liang, M. Z.; Meek, S. J. Synthesis of Quaternary Carbon Stereogenic Centers by Diastereoselective Conjugate Addition of Boron-Stabilized Allylic Nucleophiles to Enones. *J. Am. Chem. Soc.* **2020**, *142* (22), 9925–9931. https://doi.org/10.1021/jacs.0c03900.

(6)	Luo, C.; Bandar, J. S. Selective Defluoroallylation of Trifluoromethylarenes. *J. Am. Chem. Soc.* **2019**, *141* (36), 14120–14125. https://doi.org/10.1021/jacs.9b07766.

(7)	Gyorfi, N.; Farkas, E.; Nemet, N.; Weber, C.; Novak, Z.; Kotschy, A. Copper-Catalyzed Trifluoromethylation of Alkoxypyridine Derivatives. *Molecules* **2020**, *25* (20), 4766. https://doi.org/10.3390/molecules25204766.

(8)	Margariti, A.; Papakonstantinou, V. D.; Stamatakis, G. M.; Demopoulos, C. A.; Schnakenburg, G.; Andreopoulou, A. K.; Giannopoulos, P.; Kallitsis, J. K.; Philippopoulos, A. I. Substituted Pyridine-Quinoline Ligands as Building Blocks for Neutral Rhodium(III) Complexes. Synthesis, Structural Characterization Studies and Anti-Platelet Activity towards the Platelet-Activating Factor (PAF). *Polyhedron* **2020**, *178*, 114336. https://doi.org/10.1016/j.poly.2019.114336.

(9)	Wang, D.; Chen, H.-G.; Tian, X.-C.; Liang, X.-X.; Chen, F.-Z.; Gao, F. Room-Temperature Palladium-Catalysed Suzuki–Miyaura Coupling of Arylboric Acid with Aryl Chlorides. *RSC Adv.* **2015**, *5* (129), 107119–107122. https://doi.org/10.1039/C5RA19790B.

(10)	Clarke, J. J.; Maekawa, Y.; Nambo, M.; Crudden, C. M. Borenium-Catalyzed Reduction of Pyridines through the Combined Action of Hydrogen and Hydrosilane. *Org. Lett.* **2021**, *23* (17), 6617–6621. https://doi.org/10.1021/acs.orglett.1c01892.

(11)	Zhu, M.; Wang, Q.-L.; Huang, H.; Mao, G.; Deng, G.-J. General Defluoroalkylation of Trifluoromethylarenes with Both Electron-Donating and -Withdrawing Alkenes. *J. Org. Chem.* **2024**, *89* (17), 12591–12609. https://doi.org/10.1021/acs.joc.4c01531.

(12)	Chang, F.; Liu, Y. Palladium-Catalyzed Deacetonative Coupling of Aryl Propargylic Alcohols with Aryl Chlorides in Water. *Synthetic Communications* **2017**, *47* (10), 961–967. https://doi.org/10.1080/00397911.2017.1297457.

(13)	Kuriyama, M.; Hanazawa, N.; Abe, Y.; Katagiri, K.; Ono, S.; Yamamoto, K.; Onomura, O. *N* - and *O* -Arylation of Pyridin-2-Ones with Diaryliodonium Salts: Base-Dependent Orthogonal Selectivity under Metal-Free Conditions. *Chem. Sci.* **2020**, *11* (31), 8295–8300. https://doi.org/10.1039/D0SC02516J.

(14)	Bodé, N. E.; McGuire, R. T.; Stradiotto, M. Bisphosphine/Nickel-Catalyzed C–O Cross-Coupling of Phenols with Chloropyridine and Related Electrophiles. *Org. Lett.* **2022**, *24* (49), 8986–8989. https://doi.org/10.1021/acs.orglett.2c03587.

(15)	Muta, R.; Torigoe, T.; Kuninobu, Y. 3-Position-Selective C–H Trifluoromethylation of Pyridine Rings Based on Nucleophilic Activation. *Org. Lett.* **2022**, *24* (44), 8218–8222. https://doi.org/10.1021/acs.orglett.2c03327.

(16)	Lanni, E. L.; Bosscher, M. A.; Ooms, B. D.; Shandro, C. A.; Ellsworth, B. A.; Anderson, C. E. Synthesis of Substituted *N* -Benzyl Pyridones via an *O* - to *N* -Alkyl Migration. *J. Org. Chem.* **2008**, *73* (16), 6425–6428. https://doi.org/10.1021/jo800866w.

(17)	Sośnicki, J. G.; Struk, Ł.; Idzik, T.; Maciejewska, G. Scope and Limitations of the Synthesis of Functionalized Quinolizidinones and Related Compounds by a Simple Precursor Approach via Addition of Lithium Allylmagnesates to 2-Pyridones and RCM as Key Steps. *Tetrahedron* **2014**, *70* (45), 8624–8635. https://doi.org/10.1016/j.tet.2014.09.043.

(18)	Murugesan, T.; Moulana Mahal, S. H.; Arayil Vennoli, K.; Karthikeyan, D.; Kaliyamoorthy, A. Copper-Catalyzed Regioselective Imidation of 2-Pyridones. *Org. Lett.* **2024**, *26* (15), 3048–3053. https://doi.org/10.1021/acs.orglett.4c00681.

(19)	Lapierre, R.; Le, T. M. T.; Schiavi, B.; Thevenet, D.; Bazin, M.; Buzdygon, R.; Jubault, P.; Poisson, T. Photocatalytic and Photoinduced Phosphonylation of Aryl Iodides: A Batch and Flow Study. *Org. Process Res. Dev.* **2024**, *28* (5), 1436–1446. https://doi.org/10.1021/acs.oprd.2c00379.

(20)	Yue, W.; Martin, R. α‐Difluoroalkylation of Benzyl Amines with Trifluoromethylarenes. *Angew Chem Int Ed* **2023**, *62* (40), e202310304. https://doi.org/10.1002/anie.202310304.

(21)	Angew Chem Int Ed - 2023 - Yue - ‐Difluoroalkylation of Benzyl Amines with Trifluoromethylarenes.Pdf.

(22)	Lee, H.; Lee, Y.; Cho, S. H. Palladium-Catalyzed Chemoselective Negishi Cross-Coupling of Bis[(Pinacolato)Boryl]Methylzinc Halides with Aryl (Pseudo)Halides. *Org. Lett.* **2019**, *21* (15), 5912–5916. https://doi.org/10.1021/acs.orglett.9b02050.

(23)	Green, J. C.; Zanghi, J. M.; Meek, S. J. Diastereo- and Enantioselective Synthesis of Homoallylic Amines Bearing Quaternary Carbon Centers. *J. Am. Chem. Soc.* **2020**, *142* (4), 1704–1709. https://doi.org/10.1021/jacs.9b11529.

(24)	Grave, C.; Lentz, D.; Schäfer, A.; Samorì, P.; Rabe, J. P.; Franke, P.; Schlüter, A. D. Shape-Persistant Macrocycles with Terpyridine Units: Synthesis, Characterization, and Structure in the Crystal. *J. Am. Chem. Soc.* **2003**, *125* (23), 6907–6918. https://doi.org/10.1021/ja034029p.

(25)	Ouach, A.; Pin, F.; Bertrand, E.; Vercouillie, J.; Gulhan, Z.; Mothes, C.; Deloye, J.-B.; Guilloteau, D.; Suzenet, F.; Chalon, S.; Routier, S. Design of Α7 Nicotinic Acetylcholine Receptor Ligands Using the (Het)Aryl-1,2,3-Triazole Core: Synthesis, in Vitro Evaluation and SAR Studies. *European Journal of Medicinal Chemistry* **2016**, *107*, 153–164. https://doi.org/10.1016/j.ejmech.2015.11.001.

(26)	St-Pierre, G.; Ladouceur, S.; Fortin, D.; Zysman-Colman, E. Fraternal Twin Iridium Hemicage Chelates. *Dalton Trans.* **2011**, *40* (44), 11726. https://doi.org/10.1039/c1dt11236h.

(27)	Audouze, K.; Nielsen, E. Ø.; Olsen, G. M.; Ahring, P.; Jørgensen, T. D.; Peters, D.; Liljefors, T.; Balle, T. New Ligands with Affinity for the α_4_ β_2_ Subtype of Nicotinic Acetylcholine Receptors. Synthesis, Receptor Binding, and 3D-QSAR Modeling. *J. Med. Chem.* **2006**, *49* (11), 3159–3171. https://doi.org/10.1021/jm058058h.

(28)	Liu, S.; Liang, Y.; Xu, L.; Dong, J. Base‐Regulated Synthesis of the Bestmann‐Ohira or Seyferth‐Gilbert Reagent Utilizing FSO_2_ N_3_. *Eur J Org Chem* **2024**, *27* (17), e202301282. https://doi.org/10.1002/ejoc.202301282.

(29)	Larock, R. C.; Yum, E. K.; Refvik, M. D. Synthesis of 2,3-Disubstituted Indoles via Palladium-Catalyzed Annulation of Internal Alkynes. *J. Org. Chem.* **1998**, *63* (22), 7652–7662. https://doi.org/10.1021/jo9803277.

(30)	Thomas, A. M.; Asha, S.; Menon, R.; Anilkumar, G. One‐Pot Synthesis of Benzofurans via Cu–Catalyzed Tandem Sonogashira Coupling‐Cyclization Reactions. *ChemistrySelect* **2019**, *4* (19), 5544–5547. https://doi.org/10.1002/slct.201900519.

(31)	Farooq, T.; Sydnes, L. K. pDapeerbenzylation of Functionalized 4- and 5-Substituted 1,2,3-Triazoles. *New York* **2012**.

(32)	Yao, T.; Zhao, C.; Li, C.; Song, Q. Photoinduced Assembly of Diverse Homoallylic Boronates from *Gem*-Diborons. *Org. Lett.* **2025**, *27* (11), 2597–2601. https://doi.org/10.1021/acs.orglett.5c00259.

(33)	Li, H.; Shangguan, X.; Zhang, Z.; Huang, S.; Zhang, Y.; Wang, J. Formal Carbon Insertion of N- Tosylhydrazone into B–B and B–Si Bonds: gem-Diborylation and gem-Silylborylation of sp3 Carbon. Org. Lett. 2014, 16, 2, 448–451

- **^dff1^H, ^13^C, and ^19^F NMR Spectra Characterization**

**^1^H NMR (600 MHz, CDCl_3_) (S6)**

**^13^C NMR (151 MHz, CDCl_3_) (S6)**

**^1^H NMR (600 MHz, CDCl_3_) (S7)**

**^13^C NMR (151 MHz, CDCl_3_) (S7)**

**^1^H NMR (600 MHz, CDCl_3_) (S9)**

**^13^C NMR (151 MHz, CDCl_3_) (S9)**

**^1^H NMR (600 MHz, CDCl_3_) (S10)**

**^13^C NMR (151 MHz, CDCl_3_) (S10)**

**^1^H NMR (600 MHz, CDCl_3_) (S11)**

**^13^C NMR (151 MHz, CDCl_3_) (S11)**

**^1^H NMR (600 MHz, CDCl_3_) (S12)**

**^13^C NMR (151 MHz, CDCl_3_) (S12)**

**^1^H NMR (600 MHz, CDCl_3_)**

**^13^C NMR (151 MHz, CDCl_3_)**

**^11^B NMR ( 128 MHz, CDCl_3_)**

**^1^H NMR ( 600 MHz, CDCl_3_) (S13)**

**^13^C NMR (151 MHz, CDCl_3_)** **(S13)**

**^19^F NMR (377 MHz, CDCl_3_)** **(S13)**

**^1^H NMR (600 MHz, CDCl_3_)** **(S14)**

**^13^C NMR (151 MHz, CDCl_3_)** **(S14)**

**^19^F NMR (377 MHz, CDCl_3_)** **(S13)**

**^1^H NMR (600 MHz, CDCl_3_)** **(S15)**

**^13^C NMR (151 MHz, CDCl_3_) (S15)**

**^19^F NMR (377 MHz, CDCl_3_) (S15)**

**^1^H NMR ( 600 MHz, CDCl_3_) (S16)**

**^13^C NMR (151 MHz, CDCl_3_)** **(S16)**

**^19^F NMR (377 MHz, CDCl_3_) (S16)**

**^1^H NMR ( 600 MHz, CDCl_3_) (S17)**

**^13^C NMR (151 MHz, CDCl_3_)** **(S17)**

**^19^F NMR (377 MHz, CDCl_3_) (S17)**

**^1^H NMR ( 600 MHz, CDCl_3_) (S18)**

**^13^C NMR (151 MHz, CDCl_3_)** **(S18)**

**^19^F NMR (377 MHz, CDCl_3_) (S18)**

**^1^H NMR (600 MHz, CDCl_3_) (8a)**

**^13^C NMR (151 MHz, CDCl_3_) (8a)**

**^19^F NMR (377 MHz, CDCl_3_) (8a)**

**^1^H NMR (600 MHz, CDCl_3_) (8b)**

**^13^C NMR (151 MHz, CDCl_3_) (8b)**

**^19^F NMR (377 MHz, CDCl_3_) (8b)**

**^11^B NMR (128 MHz, CDCl_3_) (8b)**

**^1^H NMR (600 MHz, CDCl_3_) (8c)**

**^13^C NMR (151 MHz, CDCl_3_) (8c)**

**^19^F NMR (377 MHz, CDCl_3_) (8c)**

**^1^H NMR (600 MHz, CDCl_3_) (8d)**

**^13^C NMR (151 MHz, CDCl_3_) (8d)**

**^19^F NMR (377 MHz, CDCl_3_) (8d)**

**^1^H NMR (600 MHz, CDCl_3_) (8e)**

**^13^C NMR (151 MHz, CDCl_3_) (8e)**

**^19^F NMR (377 MHz, CDCl_3_) (8e)**

**^1^H NMR (600 MHz, CDCl_3_) (8f)**

**^13^C NMR (151 MHz, CDCl_3_) (8f)**

**^19^F NMR (377 MHz, CDCl_3_) (8f)**

**^1^H NMR (600 MHz, CDCl_3_) (8g)**

**^13^C NMR (151 MHz, CDCl_3_) (8g)**

**^19^F NMR (377 MHz, CDCl_3_) (8g)**

**^1^H NMR (600 MHz, CDCl_3_) (8h)**

**^13^C NMR (151 MHz, CDCl_3_) (8h)**

**^19^F NMR (377 MHz, CDCl_3_) (8h)**

**^1^H NMR (600 MHz, CDCl_3_) (8i)**

**^13^C NMR (151 MHz, CDCl_3_) (8i)**

**^19^F NMR (377 MHz, CDCl_3_) (8i)**

**^1^H NMR (600 MHz, CDCl_3_) (8j)**

**^13^C NMR (151 MHz, CDCl_3_) (8j)**

**^19^F NMR (377 MHz, CDCl_3_) (8j)**

**^1^H NMR (600 MHz, CDCl_3_) (8k)**

**^13^C NMR (151 MHz, CDCl_3_) (8k)**

**^19^F NMR (377 MHz, CDCl_3_) (8k)**

**^1^H NMR (600 MHz, CDCl_3_) (8n)**

**^13^C NMR (151 MHz, CDCl_3_) (8n)**

**^19^F NMR (377 MHz, CDCl_3_) (8n)**

**^1^H NMR (600 MHz, CDCl_3_) (8m)**

**^13^C NMR (151 MHz, CDCl_3_) (8m)**

**^19^F NMR (377 MHz, CDCl_3_) (8m)**

**^1^H NMR (400 MHz, CDCl_3_) (8n)**

**^13^C NMR (101 MHz, CDCl_3_) (8n)**

**^19^F NMR (377 MHz, CDCl_3_) (8n)**

**^1^H NMR (600 MHz, CDCl_3_) (8o)**

**^13^C NMR (151 MHz, CDCl_3_) (8o)**

**^19^F NMR (377 MHz, CDCl_3_) (8o)**

**^1^H NMR (600 MHz, CDCl_3_) (8p)**

**^13^C NMR (151 MHz, CDCl_3_) (8p)**

**^19^F NMR (377 MHz, CDCl_3_) (8p)**

**^1^H NMR (600 MHz, CDCl_3_) (8q)**

**^13^C NMR (151 MHz, CDCl_3_) (8q)**

**^19^F NMR (377 MHz, CDCl_3_) (8q)**

**^1^H NMR (600 MHz, CDCl_3_) (8r)**

**^13^C NMR (151 MHz, CDCl_3_) (8r)**

**^19^F NMR (377 MHz, CDCl_3_) (8r)**

**^1^H NMR (600 MHz, CDCl_3_) (8s)**

**^13^C NMR (151 MHz, CDCl_3_) (8s)**

**^19^F NMR (377 MHz, CDCl_3_) (8s)**

**^1^H NMR ( 600 MHz, CDCl_3_) (8t)**

**^13^C NMR (151 MHz, CDCl_3_) (8t)**

**^19^F NMR (377 MHz, CDCl_3_) (8t)**

**^1^H NMR ( 600 MHz, CDCl_3_) (8u)**

**^13^C NMR (151 MHz, CDCl_3_) (8u)**

**^19^F NMR (377 MHz, CDCl_3_) (8u)**

**^1^H NMR ( 600 MHz, CDCl_3_) (8v)**

**^13^C NMR (151 MHz, CDCl_3_) (8v)**

**^19^F NMR (377 MHz, CDCl_3_) (8v)**

**^1^H NMR (400 MHz, CDCl_3_) (8w)**

**^13^C NMR (101 MHz, CDCl_3_) (8w)**

**^19^F NMR (377 MHz, CDCl_3_) (8w)**

**^1^H NMR ( 600 MHz, CDCl_3_) (8x)**

**^13^C NMR (101 MHz, CDCl_3_) (8x)**

**^19^F NMR (377 MHz, CDCl_3_) (8x)**

**^1^H NMR (600 MHz, CDCl_3_) (9a)**

**^13^C NMR (151 MHz, CDCl_3_) (9a)**

**^19^F NMR (377 MHz, CDCl_3_) (9a)**

**^1^H NMR (400 MHz, CDCl_3_) (9b)**

**^13^C NMR (101 MHz, CDCl_3_) (9b)**

**^19^F NMR (377 MHz, CDCl_3_) (9b)**

**^1^H NMR (600 MHz, CDCl_3_) (9c)**

**^13^C NMR (151 MHz, CDCl_3_) (9c)**

**^19^F NMR (377 MHz, CDCl_3_) (9c)**

**^1^H NMR ( 600 MHz, CDCl3) (9d)**

**^13^C NMR (151 MHz, CDCl_3_) (9d)**

**^19^F NMR (377 MHz, CDCl_3_) (9d)**

**^1^H NMR ( 600 MHz, CDCl3) (9e)**

**^13^C NMR (151 MHz, CDCl_3_) (9e)**

**^19^F NMR (377 MHz, CDCl_3_) (9e)**

**^1^H NMR ( 600 MHz, CDCl_3_) (9f)**

**^13^C NMR (151 MHz, CDCl_3_) (9f)**

**^19^F NMR (377 MHz, CDCl_3_) (9f)**

**^1^H NMR ( 600 MHz, CDCl3) (9g)**

**^13^C NMR (151 MHz, CDCl_3_) (9g)**

**^19^F NMR (377 MHz, CDCl_3_) (9g)**

**^1^H NMR ( 600 MHz, CDCl3) (9h)**

**^13^C NMR (151 MHz, CDCl_3_) (9h)**

**^19^F NMR (377 MHz, CDCl_3_) (9h)**

**^1^H NMR ( 600 MHz, CDCl3) (9i)**

**^13^C NMR (151 MHz, CDCl_3_) (9i)**

**^19^F NMR (377 MHz, CDCl_3_) (9i)**

**^1^H NMR 1D-NOESY (600 MHz, CDCl_3_)**

**^1^H NMR ( 600 MHz, CDCl_3_) (9j)**

**^13^C NMR (151 MHz, CDCl_3_) (9j)**

**^19^F NMR (377 MHz, CDCl_3_) (9j)**

**^1^H NMR ( 600 MHz, CDCl_3_) (9k)**

**^13^C NMR (151 MHz, CDCl_3_) (9k)**

**^19^F NMR (377 MHz, CDCl_3_ (9k)**

**^1^H NMR (600 MHz, CDCl_3_) (9l)**

**^13^C NMR (151 MHz, CDCl_3_) (9l)**

**^19^F NMR (377 MHz, CDCl_3_) (9l)**

**^1^H NMR ( 600 MHz, CDCl3) (11a)**

**^13^C NMR (151 MHz, CDCl_3_) (11a)**

**^19^F NMR (377 MHz, CDCl_3_) (11a)**

**^1^H NMR ( 600 MHz, CDCl3) (11b)**

**^13^C NMR (151 MHz, CDCl_3_) (11b)**

**^19^F NMR (377 MHz, CDCl_3_) (11b)**

**^1^H NMR ( 600 MHz, CDCl3) (11c)**

**^13^C NMR (151 MHz, CDCl_3_) (11c)**

**^19^F NMR (377 MHz, CDCl_3_) (11c)**

**^1^H NMR (600 MHz, CDCl_3_) (11d)**

**^13^C NMR (151 MHz, CDCl_3_) (11d)**

**^19^F NMR (377 MHz, CDCl_3_) (11d)**

**^1^H NMR ( 600 MHz, CDCl3) (11e)**

**^13^C NMR (151 MHz, CDCl_3_) (11e)**

**^19^F NMR (377 MHz, CDCl_3_) (11e)**

**^1^H NMR (600 MHz, CDCl_3_) (11f)**

**^13^C NMR (151 MHz, CDCl_3_) (11f)**

**^19^F NMR (377 MHz, CDCl_3_) (11f)** Decoupling

**^1^H NMR (600 MHz, CDCl_3_) (11g)**

**^13^C NMR (151 MHz, CDCl_3_) (11g)**

**^19^F NMR (377 MHz, CDCl_3_) (11g)**

**^1^H NMR (600 MHz, CDCl_3_) (12a)**

**^1^H NMR (600 MHz, CDCl_3_) (12b)**

**^1^H NMR (600 MHz, CDCl_3_) (12c)**

**^1^H NMR (600 MHz, CDCl_3_) (12d)**

**^13^C NMR (151 MHz, CDCl_3_) (12d)**

**^1^H NMR (600 MHz, CDCl_3_) (12e)**

**^13^C NMR (151 MHz, CDCl_3_) (12e)**

**^1^H NMR (600 MHz, CDCl_3_) (12f)**

**^1^H NMR (600 MHz, CDCl_3_) (12g)**

**^13^C NMR (151 MHz, CDCl_3_) (12g)**

**^1^H NMR ( 600 MHz, CDCl_3_) (12l)**

**^13^C NMR (151 MHz, CDCl_3_) (12l)**

**^19^F NMR (377 MHz, CDCl_3_) (12l)**

**^1^H NMR (600 MHz, CDCl_3_) (12j)**

**^13^C NMR (151 MHz, CDCl_3_) (12j)**

**^1^H NMR (600 MHz, CDCl_3_) (12k)**

**^13^C NMR (151 MHz, CDCl_3_) (12k)**

**^1^H NMR (600 MHz, CDCl_3_) (12l)**

**^13^C NMR (151 MHz, CDCl_3_) (12l)**

**^1^H NMR (600 MHz, CDCl_3_) (13)**

**^13^C NMR (151 MHz, CDCl_3_) (13)**

**^19^F NMR (377 MHz, CDCl_3_) (13)**

**^1^H NMR (600 MHz, CDCl_3_) (14)**

**^13^C NMR (151 MHz, CDCl_3_) (14)**

**^19^F NMR (377 MHz, CDCl_3_) (14)**

**^1^H NMR (600 MHz, CDCl_3_) (15)**

**^13^C NMR (151 MHz, CDCl_3_) (15)**

**^19^F NMR (377 MHz, CDCl_3_) (15)**

**^1^H NMR (600 MHz, CDCl_3_) (16)**

**^13^C NMR (151 MHz, CDCl_3_) (16)**

**^19^F NMR (377 MHz, CDCl_3_) (16)**

**^1^H NMR (600 MHz, CDCl_3_) (17)**

**^13^C NMR (151 MHz, CDCl_3_) (17)**

**^19^F NMR (377 MHz, CDCl_3_) (17)**

**^1^H NMR (600 MHz, CDCl_3_) (18)**

**^13^C NMR (151 MHz, CDCl_3_) (18)**

**^1^H NMR ( 600 MHz, Acetone) (19)**

**^13^C NMR (151 MHz, Acetone) (19)**

**^1^H NMR ( 600 MHz, CDCl_3_) (20)**

**^13^C NMR (151 MHz, CDCl_3_) (20)**
